# Rare variant analysis in eczema identifies exonic variants in *DUSP1*, *NOTCH4* and *SLC9A4*

**DOI:** 10.1038/s41467-021-26783-x

**Published:** 2021-11-16

**Authors:** Sarah Grosche, Ingo Marenholz, Jorge Esparza-Gordillo, Aleix Arnau-Soler, Erola Pairo-Castineira, Franz Rüschendorf, Tarunveer S. Ahluwalia, Catarina Almqvist, Andreas Arnold, Australian Asthma Genetics Consortium (AAGC), Hansjörg Baurecht, Hans Bisgaard, Klaus Bønnelykke, Sara J. Brown, Mariona Bustamante, John A. Curtin, Adnan Custovic, Shyamali C. Dharmage, Ana Esplugues, Mario Falchi, Dietmar Fernandez-Orth, Manuel A. R. Ferreira, Andre Franke, Sascha Gerdes, Christian Gieger, Hakon Hakonarson, Patrick G. Holt, Georg Homuth, Norbert Hubner, Pirro G. Hysi, Marjo-Riitta Jarvelin, Robert Karlsson, Gerard H. Koppelman, Susanne Lau, Manuel Lutz, Patrik K. E. Magnusson, Guy B. Marks, Martina Müller-Nurasyid, Markus M. Nöthen, Lavinia Paternoster, Craig E. Pennell, Annette Peters, Konrad Rawlik, Colin F. Robertson, Elke Rodriguez, Sylvain Sebert, Angela Simpson, Patrick M. A. Sleiman, Marie Standl, Dora Stölzl, Konstantin Strauch, Agnieszka Szwajda, Albert Tenesa, Philip J. Thompson, Vilhelmina Ullemar, Alessia Visconti, Judith M. Vonk, Carol A. Wang, Stephan Weidinger, Matthias Wielscher, Catherine L. Worth, Chen-Jian Xu, Young-Ae Lee

**Affiliations:** 1 Max-Delbrück-Center (MDC) for Molecular Medicine, Berlin, Germany; 2 Clinic for Pediatric Allergy, Experimental and Clinical Research Center, Charité University Medical Center, Berlin, Germany; 3 Roslin Institute, University of Edinburgh, Edinburgh, UK; 4 MRC Human Genetics Unit, Institute of Genetics and Cancer, University of Edinburgh, Western General Hospital, Edinburgh, UK; 5 COPSAC, Copenhagen Prospective Studies on Asthma in Childhood, Herlev and Gentofte Hospital, University of Copenhagen, Copenhagen, Denmark; 6 Steno Diabetes Center Copenhagen, Gentofte, Denmark; 7 Department of Medical Epidemiology and Biostatistics, Karolinska Institute, Stockholm, Sweden; 8 Astrid Lindgren Children’s Hospital, Karolinska University Hospital, Stockholm, Sweden; 9 Clinic and Polyclinic of Dermatology, University Medicine Greifswald, Greifswald, Germany; 10 Department of Dermatology, Allergology and Venereology, University Hospital Schleswig-Holstein, Kiel, Germany; 11 Institute of Genetics and Molecular Medicine, The University of Edinburgh, Edinburgh, UK; 12 ISGlobal, Centre for Research in Environmental Epidemiology (CREAL), Barcelona, Spain; 13 Division of Infection Immunity and Respiratory Medicine, School of Biological Sciences, The University of Manchester, Manchester Academic Health Science Centre and Manchester University NHS Foundation Trust, Manchester, UK; 14 National Lung and Heart Institute, Imperial College London, London, UK; 15 Centre for Epidemiology and Biostatistics, Melbourne School of Population and Global Health, The University of Melbourne, Melbourne, Australia; 16 Nursing School, University of Valencia, FISABIO-University Jaume I-University of Valencia Joint Research Unit of Epidemiology and Environmental Health, CIBERESP, Valencia, Spain; 17 Department of Twins Research and Genetic Epidemiology, King’s College London, London, UK; 18 Genetics and Computational Biology, QIMR Berghofer Medical Research Institute, Brisbane, Australia; 19 Institute of Clinical Molecular Biology, Christian-Albrechts-University of Kiel, Kiel, Germany; 20 Research Unit Molecular Epidemiology, Helmholtz Center Munich – German Research Center for Environmental Health, Neuherberg, Germany; 21 Center for Applied Genomics, Children’s Hospital of Philadelphia, and Division of Human Genetics, Department of Pediatrics, The Perelman School of Medicine, University of Pennsylvania, Philadelphia, PA USA; 22 Telethon Kids Institute, The University of Western Australia, Perth, Australia; 23 Department of Functional Genomics, Interfaculty Institute for Genetics and Functional Genomics, University Medicine Greifswald, Greifswald, Germany; 24 Department of Epidemiology and Biostatistics, MRC-PHE Centre for Environment & Health, School of Public Health, Imperial College London, London, UK; 25 Center for Life Course Health Research, Faculty of Medicine, University of Oulu, Oulu, Finland; 26 Biocenter Oulu, University of Oulu, Oulu, Finland; 27 Department of Pediatric Pulmonology and Pediatric Allergology, University of Groningen, University Medical Center Groningen, GRIAC Research Institute, Groningen, the Netherlands; 28 Department of Pediatric Pulmonology, Immunology and Intensive Care Medicine, Charité University Medical Center, Berlin, Germany; 29 Institute of Genetic Epidemiology, Helmholtz Center Munich–German Research Center for Environmental Health, Neuherberg, Germany; 30 Woolcock Institute of Medical Research, University of Sydney, Sydney, Australia; 31 Institute for Medical Information Processing, Biometry, and Epidemiology (IBE), Faculty of Medicine, LMU Munich, Munich, Germany; 32 Institute of Medical Biostatistics, Epidemiology and Informatics (IMBEI), University Medical Center, Johannes Gutenberg University, Mainz, Germany; 33 Institute of Human Genetics, University of Bonn, School of Medicine & University Hospital Bonn, Bonn, Germany; 34 MRC Integrative Epidemiology Unit, Population Health Sciences, Bristol Medical School, University of Bristol, Bristol, UK; 35 School of Medicine and Public Health, Faculty of Medicine and Health, The University of Newcastle, Newcastle, Australia; 36 Institute of Epidemiology, Helmholtz Center Munich–German Research Center for Environmental Health, Neuherberg, Germany; 37 Respiratory Research, Murdoch Children’s Research Institute, Melbourne, Australia; 38 Centre for Global Health Research, Usher Institute of Population Health Sciences and Informatics, Edinburgh, UK; 39 Institute for Respiratory Health and Centre for Respiratory Health, School of Biomedical Sciences, University of Western Australia, Nedlands, Australia; 40 Department of Epidemiology, University of Groningen, University Medical Center Groningen, GRIAC Research Institute, Groningen, the Netherlands; 41 Department of Gastroenterology, Hepatology and Endocrinology, Centre for individualized infection medicine (CIIM), Hannover Medical School, Hannover, Germany; 42 Present Address: CeMM Research Center for Molecular Medicine of the Austrian Academy of Sciences, Vienna, Austria; 43 Present Address: GlaxoSmithKline, Stevenage, UK; 44 Present Address: Department of Epidemiology and Preventive Medicine, University Regensburg, Regensburg, Germany

**Keywords:** Genome-wide association studies, Atopic dermatitis, Skin diseases, Genetic predisposition to disease, Rare variants

## Abstract

Previous genome-wide association studies revealed multiple common variants involved in eczema but the role of rare variants remains to be elucidated. Here, we investigate the role of rare variants in eczema susceptibility. We meta-analyze 21 study populations including 20,016 eczema cases and 380,433 controls. Rare variants are imputed with high accuracy using large population-based reference panels. We identify rare exonic variants in *DUSP1*, *NOTCH4*, and *SLC9A4* to be associated with eczema. In *DUSP1* and *NOTCH4* missense variants are predicted to impact conserved functional domains. In addition, five novel common variants at *SATB1-AS1*/*KCNH8*, *TRIB1*/*LINC00861*, *ZBTB1*, *TBX21*/*OSBPL7*, and *CSF2RB* are discovered. While genes prioritized based on rare variants are significantly up-regulated in the skin, common variants point to immune cell function. Over 20% of the single nucleotide variant-based heritability is attributable to rare and low-frequency variants. The identified rare/low-frequency variants located in functional protein domains point to promising targets for novel therapeutic approaches to eczema.

## Abstract (web summary)

Genetic studies of eczema to date have mostly explored common genetic variation. Here, the authors perform a large meta-analysis for common and rare variants and discover 8 loci associated with eczema. Over 20% of the heritability of the condition is attributable to rare variants.

## Introduction

Eczema is a chronic inflammatory skin disease that is often associated with immunoglobulin E (IgE)-mediated allergies. Like in other complex human diseases, there is a substantial heritable contribution to the disease risk. In recent years, several large genome-wide association studies (GWAS) identified a total of 32 susceptibility loci for eczema^1–9^. However, despite increasing sample sizes, the reported associations of single nucleotide polymorphisms (SNPs) have explained only a small fraction of the estimated heritability of eczema^6,10^.

It has been hypothesized that missing heritability may originate from rare risk variants that are not well captured on SNP arrays designed to study common variants with a minor allele frequency (MAF) > 0.05^11^. Thus, the majority of low-frequency SNPs (0.01 ≤ MAF < 0.05) and rare variants (MAF < 0.01) were out of reach in this approach. Furthermore, imputation of SNPs with low allele frequency at high quality was unattainable since until recently, a reference panel of sufficient size was not available.

There is increasing evidence that rare variants contribute to disease risk not only in Mendelian diseases but also in complex diseases. Recent studies suggested a role for low-frequency and rare variants in a number of complex disorders including inflammatory bowel disease, asthma, and cancer^12–14^. With the Haplotype Reference Consortium panel (HRC), a new resource has now become available including 32,488 sequenced individuals covering almost 65,000 haplotypes^15^. This allows for the first time the reliable imputation of rare variants.

In our meta-GWAS on rare variants in eczema, we uncover disease-associated exonic variants in the genes encoding dual specificity phosphatase 1 (*DUSP1*), neurogenic locus notch homolog protein 4 (*NOTCH4*), and solute carrier family 9 member A4 (*SLC9A4*). In *DUSP1* and *NOTCH4*, the identified missense variants are located in functional domains. By extending the GWAS to common variants, another five new eczema-associated loci are detected. We estimate a contribution of over 20% for rare and low frequencies to eczema heritability.

## Results

### Study design and quality control

**Fig. 1:**
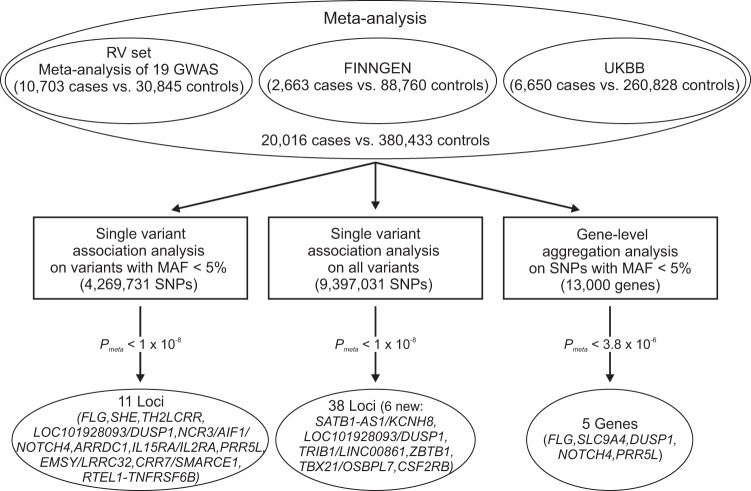
Study design of the meta-analysis of genome-wide association studies (meta-GWAS) on rare variants in eczema. Workflow of the meta-GWAS on rare variants in eczema is shown. Analyzing only rare/low-frequency variants, 11 loci were detected. In the complete data set, 38 loci were detected of which 6 were not reported to be associated with eczema or allergic disease previously. The significance threshold for the gene-level association analysis corresponds to 13,000 genes under study (carrying at least two variants with MAF < 0.05 and a CADD score ≥ 12.37). SNP single nucleotide polymorphism, MAF minor allele frequency.

In total 21 study populations comprising 20,016 cases and 380,433 controls were included in our GWAS on rare variants in eczema (Supplementary Data 1). Of them, 19 study populations with available genotypes including 10,703 cases and 30,845 controls were analyzed with RVTESTS and combined in the rare variant set (RV set). The other two data sets were the publicly available FINNGEN study comprising 2,663 cases and 88,760 controls and UK Biobank (UKBB) contributing 6,650 cases and 260,828 controls (Fig. 1). Our study had 80% power to detect risk alleles with an allele frequency of 0.004 and a risk ratio of 1.6 using an allelic model with 10% prevalence, alpha < 1 × 10^−8^, and unselected controls (Supplementary Fig. 1)^16^. Taking into account that rare alleles usually have a higher impact on disease, our study was well-powered to detect rare variants associated with eczema. The genome-wide significance threshold was set at 1 × 10^−8^, as suggested previously for GWAS analyzing variants with a MAF of 0.001^17^.

A total of 9,397,031 variants passed quality control in all three data sets. There was no evidence of inflation of the test statistics (Supplementary Fig. 2).

The combined summary statistics of 4,269,731 rare and low-frequency variants were additionally used to perform the gene-level association test which combines the effects of multiple variants within a gene thus increasing power of revealing disease-relevant genetic variants in the presence of locus heterogeneity.

### Loci associated with eczema at the single SNP level

**Fig. 2:**
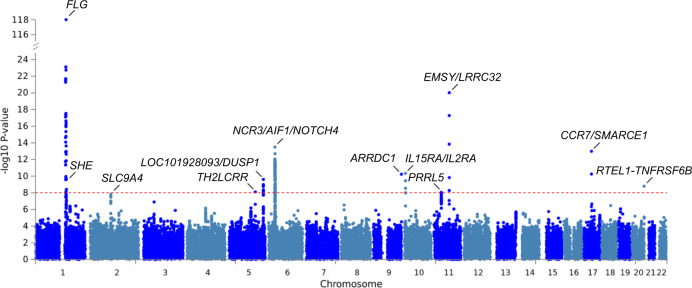
Manhattan plot of the meta-GWAS results on rare variants in eczema at the single SNP level. Meta-analysis results for all variants with a minor allele frequency < 5% are shown. For each SNP (dots), the genomic location (*x*-axis) and the association *P* value (*y*-axis) are indicated. The red line corresponds to the genome-wide significance threshold of 1 × 10^−8^.

Performing serial conditional analyses, we identified 48 independent SNPs at 38 loci to be associated with eczema at genome-wide significance (Supplementary Data 2). Focusing only on 4,269,731 rare and low-frequency variants, a total of 273 SNPs at 11 loci were associated with eczema at genome-wide significance (Fig. 2, Supplementary Data 3). The best associated rare/low-frequency SNP at each locus is presented in Table 1. Of those, four loci were exclusively associated with rare/low-frequency variants; at profilaggrin (*FLG*), *LOC101928093*/*DUSP1*, arrestin domain containing 1 (*ARRDC1*), and C-C motif chemokine receptor 7 (*CCR7*)/SWI/SNF related, matrix associated, actin-dependent regulator of chromatin, subfamily E, member 1 (*SMARCE1*), rare/low-frequency variants were identified as independently associated lead SNPs (Table 1, Supplementary Data 2). *LOC101928093*/*DUSP1* has not been reported to be associated with any allergic disease previously. Variants at *ARRCD1* and *CCR7*/*SMARCE1* were previously identified in a study on the broader phenotype “dermatitis or eczema” in UKBB^18^. Both SNPs revealed the same risk alleles and similar effect sizes in RV set, FINNGEN, and UKBB verifying association of the two loci with eczema. Notably, serial conditional analysis on chromosome 1 identified the three most frequent *FLG* null mutations at genome-wide significance with no residual association in the epidermal differentiation complex (EDC) at 1q21.3 (Supplementary Data 2).

**Table 1:** Rare and low-frequency variants associated with eczema at *P* < 1 × 10^-8^ in the single variant or in the gene-level analysis.

				RV SET			FINNGEN			UKBB			META							
Lead SNP	Chr	Pos	Ref/Alt	AF_Alt_	OR_Alt_	*P* value	AF_Alt_	OR_Alt_	*P* value	AF_Alt_	OR_Alt_	*P* value	Het *p*	OR_Alt_	*P* value	Nearest gene	Annotation	AA change	CADD	Functional impact
rs61816761	1	152285861	G/A	0.018	2.40	1.6E-36	0.003	4.05	1.2E-05	0.023	2.46	1.9E-93	0.277	2.62	3.7E-118	*FLG*	Exonic, stopgain	R501X	35	Loss-of-function
rs138726443* (*D’* = 1, *r*^*2*^ = 0)	1	152280023	G/A	0.004	2.41	7.3E-09	0.008	2.23	3.4E-06	0.005	1.91	1.4E-11	0.381	2.18	1.8E-23	*FLG*	Exonic, stopgain	R2447X	39	Loss-of-function
rs41269913	1	154461480	C/T	0.033	1.14	7.4E-03	0.023	1.44	2.3E-04	0.038	1.23	6.4E-07	0.096	1.22	2.3E-10	*SHE*	Intronic		0.07	
rs61731289*	2	103141583	G/A	0.041	1.19	4.2E-05	0.014	1.38	9.1E-03	0.048	1.12	2.9E-03	0.224	1.19	1.5E-08	*SLC9A4*	Exonic, nonsynonymous	R640K	15.24	TOPO_DOM cytoplasmic domain
rs60153262	5	131984835	C/T	0.041	1.13	3.8E-03	0.038	1.24	4.7E-03	0.039	1.21	1.3E-05	0.429	1.18	7.8E-09	*TH2LCRR*	ncRNA_intronic		0.08	
rs114503346	5	172192350	C/T	0.044	0.81	1.3E-07	0.031	0.82	1.9E-02	0.049	0.88	3.4E-03	0.348	0.84	2.5E-10	*LOC101928093/DUSP1*	Intergenic		7.67	
rs2431663* (*D’* = 0.90, *r*^*2*^ = 0.77)	5	172196711	G/T	0.951	1.21	6.8E-07	0.951	1.16	2.2E-02	0.950	1.11	1.7E-02	0.293	1.16	8.0E-09	*DUSP1*	Exonic		12.79	
rs34471628* (*D’* = 0.95, *r*^*2*^ = 0.70)	5	172196752	A/G	0.038	0.78	1.4E-08	0.028	0.84	4.2E-02	0.040	0.90	2.0E-02	0.097	0.83	1.0E-09	*DUSP1*	Exonic, nonsynonymous	Y187H	23.2	Catalytic domain
rs35084382* (*D’* = 0.90, *r*^*2*^ = 0.79)	5	172197039	T/C	0.048	0.83	1.1E-06	0.049	0.86	2.3E-02	0.049	0.90	1.4E-02	0.342	0.86	9.5E-09	*DUSP1*	Intronic		13.81	
rs34013988* (*D’* = 0.95, *r*^*2*^ = 0.70)	5	172197790	C/T	0.038	0.78	1.5E-08	0.028	0.84	5.1E-02	0.040	0.90	2.0E-02	0.097	0.83	1.4E-09	*DUSP1*	Exonic, nonsynonymous	A56T	25.1	Rhodanese-like domain
rs28732147	6	31572634	G/A	0.039	0.83	3.0E-05	0.015	0.88	2.9E-01	0.050	0.72	3.1E-12	0.059	0.79	3.2E-14	*NCR3/AIF1*	Intergenic		1.62	
rs8192591* (*D’* = 0.92, *r*^*2*^ = 0.53)	6	32185796	C/T	0.029	0.81	1.4E-05	0.013	0.80	7.6E-02	0.040	0.73	2.3E-09	0.380	0.78	2.0E-13	*NOTCH4*	Exonic, nonsynonymous	G534S	15.8	EGF-like13, calcium- binding domain
rs526945* (*D’* = 1, *r*^*2*^ = 0.005)	6	32194982	T/A	0.050	0.86	3.1E-05	0.008	0.66	9.1E-03	0.077	0.94	8.0E-02	0.026	0.85	7.6E-07	*NOTCH4/LOC101929163*	Intergenic		15.19	
rs117137535	9	140500443	G/A	0.034	1.43	5.3E-04	0.040	1.31	3.9E-04	0.025	1.27	1.2E-05	0.613	1.35	5.9E-11	*ARRDC1*	Intronic		9.97	
rs61833743	10	6050787	A/T	0.032	1.16	2.2E-03	0.026	1.23	2.3E-02	0.034	1.30	5.2E-09	0.232	1.22	4.6E-11	*IL15RA/IL2RA*	Intergenic		2.48	
rs12292945* (*D’* = 0.97, *r*^*2*^ = 0.90)	11	36426640	T/C	0.031	1.14	5.3E-03	0.058	1.18	7.2E-03	0.029	1.20	1.8E-04	0.778	1.17	1.3E-07	*PRR5L*	Intronic		14	
rs11602467* (*D’* = 0.97, *r*^*2*^ = 0.87)	11	36432830	T/G	0.033	1.11	2.1E-02	0.075	1.15	1.3E-02	0.030	1.21	1.0E-04	0.481	1.15	6.9E-07	*PRR5L*	Intronic		12.52	
rs1365121	11	36437868	T/G	0.032	1.15	3.0E-03	0.058	1.18	7.9E-03	0.030	1.23	1.4E-05	0.577	1.18	8.9E-09	*PRR5L*	Intronic		3.64	
rs55646091	11	76299431	G/A	0.046	1.19	1.4E-05	0.037	1.43	4.6E-06	0.050	1.31	1.0E-13	0.057	1.27	9.5E-21	*EMSY/LRRC32*	Intergenic		4.72	
rs112401631	17	38764524	T/A	0.015	1.13	1.1E-01	0.006	1.59	1.7E-02	0.022	1.57	7.2E-18	0.002	1.35	1.0E-13	*CCR7/SMARCE1*	Intergenic		6.58	
rs41298344	20	62294045	C/A	0.044	0.88	2.5E-03	0.043	0.83	1.1E-02	0.053	0.81	1.4E-06	0.378	0.85	1.6E-09	*RTEL1-TNFRSF6B*	ncRNA_intronic		0.18	

*SNP* single nucleotide polymorphism; *Chr* chromosome; *Pos* genomic positions (GRCh37.p13); *Ref* reference allele; *Alt* alternative allele; *AF* allele frequency; *OR* odds ratio, *Het* *p* heterogeneity *P* value; *AA* amino acid; *CADD* Combined Annotation Dependent Depletion.

^*^Significantly associated low-frequency variants from the gene-based analysis; LD (*D’*; *r*^2^) with the lead variant is indicated; rs526945_A and rs8192591_T were located on different haplotypes and were associated independently from each other (*r*^*2*^ = 0.00).

At seven loci, Src homology 2 domain containing E (*SHE*), T helper type 2 locus control region associated RNA (*TH2LCRR*), natural cytotoxicity triggering receptor 3 (*NCR3*)/allograft inflammatory factor 1 (*AIF1*), interleukin-15 receptor subunit alpha (*IL15RA*)/interleukin 2 receptor subunit alpha (*IL2RA*), proline rich 5 like (*PRR5L*), *EMSY*/leucine rich repeat containing 32 (*LRRC32*), and regulator of telomere elongation helicase 1 (*RTEL1*) common variants were more significantly associated with eczema than the respective rare/low-frequency variant although the effect sizes were smaller (Supplementary Data 4). Conditioning on the common variants abolished genome-wide significance of rare/low-frequency variants at all loci except for *TH2LCRR* and *EMSY*/*LRRC32*. Conditioning on rare/low-frequency variants often revealed a residual effect of the common variant, which may point to a larger or exclusive impact of the common variant. Alternatively, unidentified rare variants in linkage disequilibrium with a common SNP may underlie the association signal.

Genome-wide investigation of the complete data set identified 48 independent SNPs at 38 loci (Supplementary Data 2). Of those SATB1 antisense RNA 1 (*SATB1-AS1*)/potassium voltage-gated channel subfamily H member 8 (*KCNH8*), tribbles pseudokinase 1 (*TRIB1*)/*LINC00861*, zinc finger and BTB domain containing 1 (*ZBTB1*), T-box transcription factor 21 (*TBX21*)/oxysterol binding protein like 7 (*OSBPL7*), and colony stimulating factor 2 receptor subunit beta (*CSF2RB*) contain common eczema-associated SNPs which were previously not identified. All loci showed consistent effects in RV set, FINNGEN, and UKBB (Supplementary Data 2, Supplementary Fig. 24–28). Common variants in or near the genes encoding RUNX family transcription factor 3 (*RUNX3*), TNF superfamily member 4 (*TNFSF4*), D-2-hydroxyglutarate dehydrogenase (*D2HGDH*), DEAD-box helicase 6 (*DDX6)*/C-X-C motif chemokine receptor 5 (*CXCR5*), TNF receptor associated factor 3 (*TRAF3*), and mitogen-activated protein kinase kinase kinase 14 (*MAP3K14*)/Rho GTPase activating protein 27 (*ARHGAP27*) were previously associated in UKBB with a broad “dermatitis or eczema” phenotype^18,19^. We demonstrate association of these loci with the stricter definition of eczema (Supplementary Data 2).

### Replication of eczema-susceptibility loci from previous GWAS

In order to verify the eczema-susceptibility loci identified in previous GWAS^1–9^, we looked-up in our study the best-associated SNP reported at each locus (Supplementary Data 5). Using a significance threshold of *P* < 0.0015 (=0.05/32 loci), we replicated 24 out of 32 loci. An independent locus near *FLG* on chromosome 1 (rs7512552)^6^ could not be confirmed, it was eliminated after adjusting for the three identified *FLG* variants (Supplementary Data 2, Supplementary Data 5). Six SNPs previously identified in GWAS on Asian populations^2,3^ did not replicate. However at three of these loci, on chromosomes 3, 5, and 20, SNPs in close proximity which were not in LD with the previously reported SNP passed the genome-wide significance threshold (Supplementary Data 5).

### Gene-level association with eczema

Limited power is the main drawback when studying the association of individual rare variants in complex diseases. Gene-level association tests which combine the effects of multiple variants within a gene may overcome this problem in the presence of locus heterogeneity. In an explorative approach we used RV set to test different variant selection strategies and two different gene-level association tests, GRANVIL and SKAT which are implemented in the RVTESTS software package^20^ (Supplementary Data 6). Selecting variants based on the deleteriousness score (CADD) in combination with the variance-component test (SKAT) performed best for the different scenarios and identified *FLG*, *DUSP1*, and *NOTCH4* to be significantly associated with eczema in RV set.

We then performed a gene-level association test for the meta-analysis of RV set, FINNGEN, and UKBB. For the analysis, we used the SNP-wise Mean model of MAGMA^21^ which is equivalent to the SKAT model with the advantage of allowing the usage of summary statistics. We included all variants with a MAF < 0.05 and a CADD score threshold of ≥ 12.37 for deleterious variants as suggested by Kircher et al (2014)^22^ capturing approximately the 5% most deleterious mutations. 13,000 genes with at least two deleterious variants were tested for association with eczema. Genes at five loci reached genome-wide significance at *P* < 3.8 × 10^−6^ (= 0.05/13,000; Supplementary Data 7). All variants contributing significantly to the gene-level association were also included in Table 1. All rare/low-frequency variants from Table 1 revealed consistent effects in the study populations of RV set, FINNGEN, and UKBB (Supplementary Fig. 3–23). As identified in RV set, MAGMA confirmed *FLG*, *DUSP1*, and *NOTCH4* to be significantly associated with eczema in the complete data set (Supplementary Data 7). In addition, *SLC9A4* and *PRR5L* passed the genome-wide significance threshold.

Since within the EDC on chromosome 1 and the MHC on chromosome 6, SNPs in multiple genes reached genome-wide significance (Supplementary Fig. 29), we conditioned the results on the three identified *FLG* variants and the two significantly associated *NOTCH4* variants from MAGMA, rs8192591 and rs526945, and repeated the gene-level association test. All significantly associated genes within the EDC and within the MHC disappeared (Supplementary Fig. 29) suggesting that the best-associated genes *FLG* and *NOTCH4* represented the main eczema-susceptibility genes in these regions.

### Functional assessment of the rare/low-frequency eczema-associated variants

We evaluated the potential impact of the identified rare/low-frequency variants on protein function by analyzing their sequence context and reviewing their annotations in databases using the GTEx Portal^23^, MutationTaster^24^, and HaploReg v4.1^25^. We included the lead SNPs from the single variant and gene-based analysis (Table 1). In addition, all SNPs in high LD with the associated variants (*r*^*2*^ > 0.8) were identified and functionally assessed (Supplementary Data 8).

**Fig. 3:**
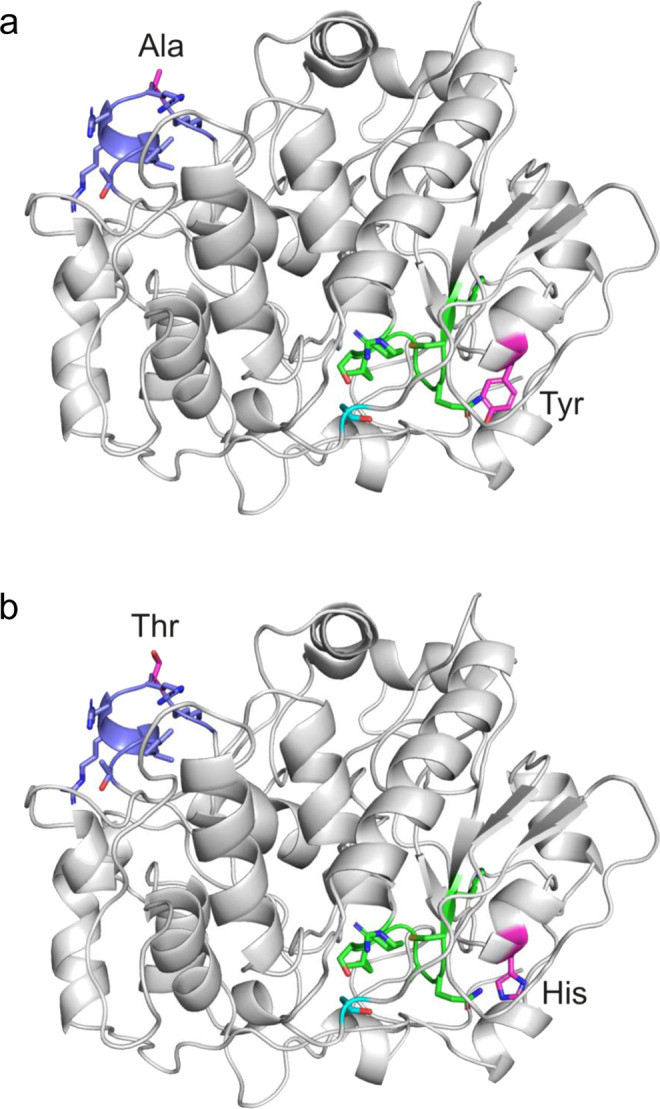
DUSP1 homology model showing (a) the wild-type protein, (b) the mutated protein. Eczema-associated variants change the amino acids (in pink) at positions 56 (alanine, Ala, to threonine, Thr) and 187 (tyrosine, Tyr, to histidine, His). The kinase-interacting motif (blue), the phosphotyrosine-binding loop (green), and Asp227 (light blue) which belongs to the catalytic site are indicated. Acid and base groups in the functional sites are marked red and blue respectively.

New exonic variants were identified in *DUSP1*, *NOTCH4*, and *SLC9A4*. In the dual specificity phosphatase 1 gene (*DUSP1*) at 5q35.1, the best associated low-frequency variant rs114503346 was in high LD (*D’* = 0.95; *r*^*2*^ = 0.70) with two low-frequency missense variants, rs34471628 and rs34013988 (Table 1). Both missense variants were in complete LD (*D’* = 1, *r*^*2*^ = 1). Accordingly, their minor alleles, rs34471628_G and rs34013988_T, are located on the same haplotype which had a protective effect on eczema. Conditioning on rs114503346 did not yield any further association signal in that region (Supplementary Data 2). *DUSP1* encodes a phosphatase which specifically dephosphorylates serine and threonine residues. *DUSP1* is ubiquitously expressed with high mRNA levels in human skin (GTEx Portal on 12/04/20)^23^. The eczema-associated variants were located within regulatory regions in a wide range of tissues including skin and blood (Supplementary Data 8). Two functional motifs were previously identified to be important for DUSP1 function, the kinase interacting motif which binds the respective kinases and the phosphotyrosine-binding loop which is the active site of DUSP1^26,27^. We used molecular modelling to investigate whether the variants identified in our study would affect these motifs and consequently impact protein function (Fig. 3).

Variant rs34013988_T leads to a substitution of alanine by threonine at residue 56 (p.Ala56Thr) and rs34471628_G changes tyrosine to histidine at residue 187 (p.Tyr187His). Both wild-type amino acids, Ala56 and Tyr187, are highly conserved among vertebrates (phastCons^28^ scores of 1). Ala56 is located within the kinase interacting motif of DUSP1 (Fig. 3a) which is found to be identical in other vertebrates and in human DUSP4 which is another member of this protein family^26^. Substitution of non-polar alanine by polar threonine may alter the kinase-binding capacity and thus affect the activity or specificity of DUSP1. Within the amino acid sequence, Tyr187 is located distant from the functional motifs. In the predicted 3D-structure of DUSP1, however, it is shifted close to the phosphotyrosine-binding loop (Fig. 3b) which is the catalytic site of DUSP1. Accordingly, replacement of Tyr187 with histidine could affect the catalytic activity of the protein. Histidine is frequently found in the active site of proteins because of its distinctive property to function as base or as acid in catalysis. Notably, His187 is located at a similar distance from the catalytic site as Asp227 which has been shown to contribute to the catalytic activity of DUSP1 as a general acid/base^27^. In addition, we found *DUSP1* expression to be dysregulated in lesional skin of eczema patients further supporting a role in eczema (Supplementary Fig. 30).

While serial conditional analysis on chromosome 6 identified three independent common SNPs (rs2844594, rs6913664 and rs13208697) within the major histocompatibility complex (MHC) (Supplementary Data 2), the gene-level analysis on rare variants identified *NOTCH4* as the best-associated gene in this region (Supplementary Data 7). Two variants with high CADD score contributed to the association with *NOTCH4*; rs8192591 and rs526945 (Table 1) which were in LD with the common variants rs6913664 and rs13208697 (Supplementary Data 4). Conditioning on the two *NOTCH4* variants eliminated genome-wide significance of rs6913664 and rs13208697 suggesting that low-frequency, functional variants may underlie association of common variants at this locus. The association signal of the third common SNP, rs2844594, remained thus representing an independent signal.

The best-associated low-frequency variant from the single SNP analysis in the MHC region, the intergenic variant rs28732147, also pointed to the functional variant rs8192591 since both SNPs were in high LD (*D’* = 0.92, *r*^*2*^ = 0.53) and revealed almost identical effect sizes (Table 1). rs8192591 is located in exon 9 of *NOTCH4* (NM_004557.4). It is a missense variant causing a substitution of nonpolar glycine to polar serine at residue 534 within the highly conserved (phastCons score of 0.97) EGF-like domain 13 (Table 1, Supplementary Data 8) which may affect the domain’s calcium-binding capacity. The second variant rs526945 is located 4.5 kb upstream of *NOTCH4* in a predicted promoter/enhancer region (CADD score of 15.2, Supplementary Data 8). Highest expression of *NOTCH4* has been reported in lung, adipose, and breast tissue, as well as in monocytes^23,29^.

On chromosome 2, the common SNP rs3755274 in *IL18R1* which revealed the strongest association signal (Supplementary Data 2) was in LD (*D’* = 0.85, *r*^*2*^ = 0.09) with the low-frequency missense variant rs61731289 in *SLC9A4* which was identified in the gene-level analysis (Supplementary Data 4, Supplementary Data 7). We propose the coding variant rs61731289 (CADD score of 15.2) changing arginine to lysine in position 640 of SLC9A4 as potential causal allele and effector transcript at this locus. *SLC9A4* is mainly expressed in stomach, at lower levels in kidney and esophageal mucosa (GTEx Portal on 12/04/20)^23^.

### Pathway enrichment analysis and tissue-specific gene expression

We performed a pathway enrichment analysis for GO terms and curated gene-sets^30^ and a tissue expression analysis for the 54 tissues included in GTEx^23^ with MAGMA^21^. In the tissue-specific gene expression analysis, we observed a significant correlation between the distribution of *P* values of SNPs and gene expression in small intestine, spleen, lung, whole blood, visceral omentum, and colon (Supplementary Fig. 31–33).

Using all GWAS SNPs, we observed a significant enrichment of immune-related pathways involved in cytokine signaling, T-cell differentiation and activation as well as enrichment of genes related to the cornified cell envelope in keratinocytes (Supplementary Data 9). Stratifying the results according to allele frequency revealed that rare/low-frequency SNPs were associated with the cornified cell envelope whereas common SNPs were associated with immune functions (Supplementary Data 10).

Moreover, we extended the analysis to candidate genes mapped from genome-wide significant variants, and evaluated their enrichment among differentially expressed gene (DEG) sets as defined for 54 different tissues. Prioritized genes from all genome-wide significant variants were enriched in sets of up-regulated DEGs in whole blood and skin (Supplementary Fig. 34a). Prioritized genes from only common variants were also enriched in sets of up-regulated DEGs in whole blood, as well as in small intestine (Supplementary Fig. 34b). In contrast, prioritized genes from rare/low-frequency variants were overrepresented in sets of up-regulated DEGs in skin and esophagus mucosa (Supplementary Fig. 34c). To exclude that the results for the rare/low-frequency variants were exclusively driven by *FLG* mutations, we repeated the analysis after conditioning on the three known *FLG* variants identified. This eliminated all prioritized genes from the epidermal differentiation complex and still identified a significant enrichment of the remaining genes in up-regulated DEGs from skin (Supplementary Fig. 34d).

### Heritability explained by rare variants

In order to quantify the contribution of rare and low-frequency variants to the heritability of eczema, we used an in house data set comprising 7,092 unrelated individuals (20.1% cases) with HRC-imputed genotypes. GREML-LDMS was used to estimate heritability for 3 frequency bins including rare variants (MAF < 0.01), low-frequency variants (0.01 < MAF < 0.05), and common variants (MAF > 0.05) respectively. In total 5,575,243 high-quality SNPs (imputed at *r*^*2*^ > 0.9) were included in the analysis. The heritability explained by rare and low-frequency variants was 4.5% and 9.1% respectively (Supplementary Data 11 and 12). They accounted for 23.0% of the SNP-based heritability supporting a substantial role for rare and low-frequency in eczema susceptibility.

## Discussion

We performed a meta-GWAS of 21 study populations including 20,016 cases and 380,433 controls to determine the role of rare variants in the development of eczema. Single SNP and gene-level analyses identified 11 loci carrying rare/low frequency variants at genome-wide significance. Exonic variants were detected in *DUSP1, NOTCH4* and *SLC9A4*. In *DUSP1* and *NOTCH4* the identified missense variants were predicted to impact conserved functional domains. The analysis of common variants yielded another five eczema-susceptibility loci not reported previously. Over 20% of the SNP-based heritability was explained by rare and low-frequency variants, emphasizing the importance of studying rare variants in eczema.

In recent years, a number of studies investigated the role of rare variants in complex diseases. They discovered single novel loci with rare or low-frequency variants associated with ulcerative colitis^12^, age-related macular degeneration^31^ or schizophrenia^32^. For eczema, an exome-chip based analysis in a discovery set of 2,000 cases and 15,000 controls identified a single new eczema susceptibility locus containing low-frequency variants in the docking protein 2 gene (*DOK2*)^33^. Utilizing HRC-imputed data allowed us to significantly increase sample size and thus the power in our study on rare variants. Accordingly, we identified rare/low-frequency variants at 11 loci including three genes which contained amino acid-changing variants not reported previously^33^. However, it is clear that much larger data sets will be required to dissect the contribution of rare variants to complex diseases. Current studies on quantitative rather than binary phenotypes identified 22 and 512 rare or low-frequency variants underlying blood pressure^34^ and various blood cell traits, respectively^35^. They investigated up to 1.3 million study participants.

We discovered eczema-associated missense variants in *DUSP1*, *NOTCH4*, and *SLC9A4*. *DUSP1* variants had a protective effect in the single variant and in the gene-level analysis. The two missense variants rs34471628 and rs34013988 were located near active sites. Both may alter the functional activity or specificity of DUSP1. Target proteins which are dephosphorylated by DUSP1 are extracellular signal-regulated kinases (ERK) 1 and 2, c-Jun N-terminal protein kinase (JNK) 1, and a kinase negative form of mitogen-activated protein kinase (MAPK) p38α^36^. Dysregulated *DUSP1* expression was reported in cultured keratinocytes of eczema patients; association of a common candidate SNP near *DUSP1* however was weak^37^. Interestingly, DUSP1 has been reported to mediate the anti-inflammatory effect of corticosteroids which are a mainstay of eczema therapy^38^. Dexamethasone-induced*DUSP1* expression repressed the ERK, JNK and p38α MAPK pathways and reduced the expression of inflammatory genes in human pulmonary epithelial cells^39^. Hence, novel therapies directed at modifying DUSP1 function may be beneficial in eczema treatment.

In the gene-level analysis focusing on rare variants with high functional impact, *NOTCH4* was the best-associated gene in the MHC region. The identified *NOTCH4* missense mutation rs8192591 was located in the EGF-like domain 13. While the number of EGF-like domains vary between the NOTCH1 and NOTCH4 receptors, the first 13 EGF-like domains are highly conserved^40^, with repeats 11–13 involved in ligand binding^41^. NOTCH4 belongs to a family of four highly conserved transmembrane receptors involved in proliferation, apoptosis, and cell fate decisions^42^. Expression of all NOTCH receptors and their ligands has been demonstrated by immunostaining in human skin sections^43^; NOTCH4 was expressed in the suprabasal layers of the epidermis and it was the major NOTCH receptor in the stratum corneum. NOTCH4 has recently been identified as a master molecular switch in a new pathway that decreases regulatory T (T_reg_) cell function in chronic inflammatory conditions^44,45^. Upregulation of NOTCH4 receptor T_reg_ cells was necessary for allergen induced airway inflammation. NOTCH4 and its downstream effector pathways were upregulated on T_reg_ cells of individuals with asthma and correlated with disease severity^44^.

Due to the high LD across the MHC region on chromosome 6, association signals are notoriously difficult to dissect. Conditioning on the two functional *NOTCH4* SNPs eliminated genome-wide significance of two of the three independently associated common SNPs, suggesting that rare functional variants may underlie the common association signals at this locus. However, an independent association signal persists in the MHC (rs2844594), indicating that additional eczema risk variants remain to be discovered in that region. Functional studies will be required to confirm a role of NOTCH4 in eczema.

On chromosome 2, the identified missense variant rs61731289 pointed to *SLC9A4* as the risk gene at this locus. However, conditioning on the low-frequency variant yielded strong residual association of the common variant rs3755274 demonstrating that other genetic risk factors exist at this locus. SLC9A4 belongs to the SLC9 family of sodium/hydrogen exchangers and is localizing to the plasma membrane in renal, intestinal, and other epithelia^46^. The eczema associated variant rs61731289 is located within the cytoplasmic tail of SLC9A4. While this region of SLC9A4 has not been functionally characterized, truncation studies of this domain in SLC9A3 have revealed its involvement in regulating ion transporter activity^47^. An *SLC9A4* knockout mouse revealed major histological changes in the gastric mucosa consistent with impaired maturation and/or differentiation of gastric epithelial cells, as well as an inflammatory infiltrate dominated by eosinophils^48^. Thus SLC9A4 may play a role in epithelial integrity and eosinophilic inflammatory responses which are also characteristic of eczema^49^.

Of the 5 new eczema-susceptibility loci captured by common variants, *SATB1-AS1*/*KCNH8*, *TRIB1*/*LINC00861*, and *TBX21*/*OSBPL7* were previously identified in a GWAS combining diverse inflammatory diseases excluding eczema^50^. Involvement of these loci in immune functions suggests that the same mechanisms also play a role in eczema. *SATB1-AS* is expressed in a wide range of tissues including the spleen, whole blood, and gut, whereas *KCNH8* encodes a potassium channel that is primarily expressed in the nervous system^23^. *SATB1-AS* expression is modified by mutations in *SATB1*^51^. *SATB1* in turn is predominantly expressed in CD4 + CD8 + lymphocytes exerting both activating and repressive regulatory functions implicated in autoimmunity and cancer^52^. The pseudokinase TRIB1 is a key regulator of eosinophil differentiation that are important effector cells in allergic inflammation^53^. Moreover, it has been shown recently that TRIB1 restrains antiviral effector T cell function^54^. Little is known about *LINC00861* function. Highest expression is found in the spleen and whole blood. Variants in *TBX21* were associated with asthma in a candidate gene study^55^. *TBX21* encodes the Th_1_-specific transcription factor T-bet which is involved in Th_1_/Th_2_ balance and suppressed atopic dermatitis-like skin inflammation in mice^56^.

*ZBTB1* which was previously identified in a GWAS as a psoriasis susceptibility locus^57^ is a critical determinant of T cell development. *ZBTB1* knockout mice completely lacked T cells^58^. The differentiation of common lymphoid progenitors of B and NK cell lineages was also affected^59^.

The *CSF2RB* locus was associated with asthma in a previous GWAS^60^. *CSF2RB* encodes the common beta subunit of the interleukin 5 (IL-5), interleukin 3 (IL-3), and granulocyte-macrophage colony-stimulating factor (GM-CSF) receptors. It plays a critical role in the regulation of Th_2_ immunity and allergic inflammation. In *Csf2rb*-deficient mice, allergen-induced expansion and accumulation of eosinophils in the lung was abolished and proliferation and activity of Th_2_ cells was reduced^61^.

Tissue-specific gene expression analysis and pathway enrichment analysis identified tissues and gene-sets related to immune functions and to skin development. Interestingly rare/low frequency variants and their target genes exerted their effects predominantly in skin which was still evident after adjusting for the strong genetic effect of the low-frequency variants in *FLG*. In contrast, common GWAS variants pointed to pathways and tissues of the immune system as supported by the reported functions of the new candidate genes from the common SNP analysis. Focusing on cutaneously expressed genes could therefore be a promising strategy for rare variant studies in eczema.

In order to avoid cost-intensive whole-exome or whole-genome sequencing we performed our study on HRC-imputed data. Before this dataset was published, imputation quality of rare variants was generally low and many rare variants were not even detected due to the low number of sequenced haplotypes of a population. HRC comprises the phased haplotypes of more than 32,000 sequenced individuals and allows imputation of rare variants with high confidence^15^. Accordingly, the majority of genome-wide significant low-frequency variants was imputed at high quality (*r*^*2*^ > 0.8) (Supplementary Data 13). We additionally determined the non-reference sensitivity (NRS) and non-reference discordancy (NRD)^62^ for the identified coding variants in an in house data set of 892 individuals whose HRC-imputed genotypes (after genotyping with Illumina’s HumanOmniExpressExome-8 SNP array) and whole exome sequences were available. Genotypes correlated very well for all coding variants (Supplementary Data 14). Even variants with an imputation quality *r*^*2*^ < 0.8 (Supplementary Data 13) confirmed association results at known eczema loci (*FLG*, *ARRCD1*, *CCR7*/*SMARCE1*). In addition, imputed loss-of-function mutations in *FLG* almost perfectly matched the previously reported allele frequencies and effect sizes^63^. Finally, effects of the identified exonic variants in *DUSP1*, *NOTCH4* and *SLC9A4* were homogeneous over all study populations, supporting the validity of the HRC-imputed approach.

Our analysis of the association between *FLG* and eczema has illustrated potential pitfalls of strategies for analyzing rare variants on the gene-level. In contrast to the single SNP analysis, gene-level tests have to take into account the genetic architecture of a locus including different effect sizes and effect directions of variants within a gene. Both, the SNP selection strategy and the gene-level association test had an impact on the results (Supplementary Data 6). SNP selection based on the deleteriousness score (CADD) in combination with the variance component test (SKAT) was most powerful for the different scenarios. Likewise a previous study on rare and low-frequency variants in eczema focused only on missense variants in the gene-level association test since they are more likely to have a deleterious effect^33^.

We restricted our analysis to variants with a MAF > 0.001. It has been suggested that rarer mutations could play a significant role in complex diseases. However, our power calculation indicated that larger populations are required to study those variants. In addition, sequence data would be essential for individual mutations which are unlikely to be included in reference data sets for imputation. Although the number of patients affected by a specific rare mutation may be low, the pinpointed genes and effects on the protein level could give important clues for unraveling pathways involved in eczema susceptibility. Our heritability estimation for rare and low-frequency variants yielded a contribution of 13.6% to the overall heritability on the observed scale (22.2% on the liability scale). For rare variants (MAF < 0.01) a heritability estimate of 4.5% on the observed scale (7.4% on the liability scale) was calculated. Mucha et al. reported a heritability estimate of 12.56% on the liability scale for rare variants included in the exome chip which is likely due to the large proportion (97%) of missense and other deleterious rare variants on the chip^33^. Interestingly, the heritability explained by the known loci was mainly attributable to common variants (Supplementary Data 11 and 12) indicating that the majority of eczema-associated rare variants remains to be discovered.

In summary, our study on rare variants in eczema identified novel exonic variants in 3 genes and intronic or intergenic variants at another 8 loci supporting the involvement of rare and low-frequency variants in the development of eczema. The localization of the missense variants and their potential functional impact on the newly-identified eczema-associated genes *DUSP1* and *NOTCH4* suggest promising targets for future therapies.

## Methods

### Study populations and phenotype definition

Characteristics of the study populations are summarized in Supplementary Data 1. The majority of them had participated in the previous genome-wide association study (GWAS) on eczema performed by the EArly Genetics and Lifecourse Epidemiology (EAGLE) Consortium^6^. Two additional studies from Sweden, CATTS, and SALTY, as well as two large population-based studies, FINNGEN and UKBB, were included. Eczema was defined based on a physician’s diagnosis according to standard diagnostic criteria^64,65^, on a doctor’s diagnosis reported in a questionnaire, or on self-reported disease. The phenotype characteristics of the individual study populations are indicated in detail in Supplementary Data 1 and Supplementary Note 1. All study populations were of European ancestry. All relevant ethical regulations were followed and informed consent was obtained from all participants or their legal guardians. All studies were approved by the local ethics committees (Supplementary Note 1).

### Genotyping and imputation

In total, 19 study populations with individual genotype data were included in “RV set”. Genotyping, quality control (QC), genotype imputation, and association analysis were performed in each study population separately and are described in detail in the Supplementary Note 1. In brief, after genotyping and appropriate QC, phasing of the genotype data into haplotypes was carried out using SHAPEIT v2^66^. Imputation of rare variants was performed using the Haplotype Reference Consortium Panel on imputation servers at the Michigan University or at the Wellcome Trust Sanger Institute^15^. In each data set, SNPs with poor imputation quality (r² or info score < 0.5), a MAF less than 0.001 or deviation from Hardy-Weinberg equilibrium in controls (*P* < 1 × 10^−12^) were excluded.

Details on genotyping, QC, genotype imputation, and association analysis in UKBB and FINNGEN are described in Supplementary Note 1.

Imputation quality of the identified exonic variants was confirmed by comparing imputed and sequenced genotypes in an in house data set of 892 individuals whose HRC-imputed genotypes (after genotyping with Illumina’s HumanOmniExpressExome-8 SNP array) and whole exome sequences were available. Non-reference sensitivity (NRS) and non-reference discordancy (NRD) were calculated (Supplementary Data 14)^62^.

### Association analysis at the single SNP level

Individual genotype data available in the 19 study populations of RV set allowed us to analyze them with RVTESTS^20^, a tool which implements a broad set of rare variant association statistics and which was specifically developed to study rare variants at the genome-wide scale. GWA analysis of the imputed SNPs with eczema was carried out per study population using a score test as implemented in the RVTESTS software^20^. A covariance matrix describing the covariance between SNPs in the population was thereby generated. After association analysis, SNPs with a minor allele count < 1 in cases or controls were excluded in each data set. To be included in the single SNP meta-analysis, variants had to be present in at least 3 study populations. Single-SNP meta-analysis was performed using summary statistics of the individual study populations and a fixed-effects model as implemented in RareMETALS2 (https://genome.sph.umich.edu/wiki/RareMETALS2), an add-on R-package to RAREMETAL^67^ for the meta-analysis of rare variant association results from binary traits. Covariance matrices were included as covariates. Subsequently, summary data on eczema from UKBB and from FINNGEN were meta-analyzed with the summary data of RV set by using the sample size method of METAL^68^ which uses *P* values and effect directions, weighted according to sample size. All SNPs with data in RV set, UKBB, and FINNGEN were included. To identify all independent association signals at a locus, we performed serial conditional analyses with GCTA^69^. A ± 1 Mb-window around each lead SNP was defined and association signals within the window were conditioned on the lead SNP. This procedure was repeated until all genome-wide signals at a locus were eliminated (Supplementary Data 3).

If a rare and a common variant were associated with eczema at genome-wide significance at a specific locus, we mutually conditioned the results using GCTA^69^. *P* values before and after conditional analysis were included in Supplementary Data 4.

### Association analysis at the gene level

In RV set different SNP selection strategies and gene-level association tests were explored. For aggregating SNPs into gene units, 3 different selection strategies were applied. First, SNPs predicted to severely impact protein levels (start lost, stop lost, stop gained, splice acceptor, splice donor) by the Ensembl Variant Effect Predictor^70^ were combined. Secondly, SNPs predicted to be missense SNPs were included on top of the first selection. Thirdly, to include potentially functional SNPs located in intronic or promotor regions of genes and to exclude coding SNPs with a predicted minor impact on protein function, a deleteriousness score for SNPs was used. For the third strategy, only SNPs with a PHRED score > 15 calculated by the Combined Annotation Dependent Depletion (CADD) tool^22^ were combined.

Two different tests implemented in RareMETALS were applied to perform association tests on the gene level by combining score statistics of rare variants per gene; the Gene- or Region-based ANalysis of Variants of Intermediate and Low-frequency (GRANVIL)^71^ and the SNP-set Kernel Association Test (SKAT)^72^. GRANVIL averages the score statistics of individual variants per gene and has most power if all variants have the same effect direction. SKAT is a variance component test and remains powerful if variants with opposite effect directions are combined. Finally, gene-level association analysis of the combined data set including FINNGEN and UKBB was conducted with MAGMA^21^ which is equivalent to SKAT with the advantage of allowing the usage of summary statistics.

### Functional annotation

Linkage disequilibrium (LD) between SNPs was calculated using the single-nucleotide polymorphism annotator with LD information from the European population of the 1000 Genomes project phase 3v5^73^. eQTLs for the lead SNPs were identified using the single-nucleotide polymorphism annotator^73^. eQTLs were only reported, if the queried SNP was the most significant eQTL SNP for a gene in a relevant tissue per LD group. Information about eQTL SNPs in relevant tissues was collected from the GTEx Consortium database version 6p^23^. SNP functional annotations were predicted using the Ensembl Variant Effect Predictor^70^ and HaploReg v4.1^25^. Pathogenicity prediction was performed using the MutationTaster^24^.

### Homology modeling

The 3D structures of both wild-type and mutant DUSP1 were modeled using a locally installed version of I-TASSER 5.1 using standard settings. Figures were prepared using PyMOL 2.4 (The PyMOL Molecular Graphics System, Version 2.4, Schrödinger, LLC).

### Pathway enrichment analysis and tissue expression analysis

Pathway enrichment analysis and tissue expression analysis both were performed with MAGMA^21^ via the FUMA webpage (https://fuma.ctglab.nl)^74^. Pathway enrichment analysis included GO terms and curated gene-sets from the Molecular Signatures Database (MSigDB)^30^. Expression profiles were from 54 tissue types from GTEx^23^. To identify a potential difference between rare and common variants, we stratified our results according to the minor allele frequency. Candidate genes were prioritized from genome-wide significant SNPs based on a combination of three SNP-to-gene mapping strategies: positional mapping (physical position on the genome within 10 kb), eQTL mapping (eQTL associations from any tissue type) and chromatin interaction mapping (3D chromatin interactions based on any built-in chromatin interaction data available) using default parameters in FUMA^74^, except for maximum *P* value of lead SNPs which was set at 1 × 10^−8^. Candidate genes were prioritized from: all variants, common variants (MAF ≤ 0.05) and rare variants (MAF < 0.05%; before and after conditioning on the 3 identified *FLG*loss-of-function variants), respectively. We tested for tissue specificity of eczema prioritized genes using hypergeometric tests to evaluate its overrepresentation in sets of differentially expressed genes (DEG; sets of genes which are more or less expressed in a specific tissue compared to others) in 54 tissue types based on GTEx v8 RNA-seq data.

### SNP-based heritability analysis

We built an in-house sample of 8,134 individuals with available genotypes and HRC-imputed data. Using GCTA^69^, we identified and removed related individuals (—grm-cutoff 0.05). SNP variants with MAF < 0.001, Hardy–Weinberg exact test < 1 × 10^−12^ and imputation quality *r*^*2*^ < 0.9 were excluded using PLINK^75^. The final dataset included 7,092 unrelated individuals (20.1% eczema cases) and 5,575,243 high-quality SNPs. Principal Components (PCs) were calculated in GCTA. LD score was calculated for all SNPs as the sum of LD *r*^2^ between a SNP and all the rest of SNPs within a region of 200 kb. All 5,575,242 SNPs were stratified in 4 bins based on the segment-based LD score of the SNPs and each bin further divided in 3 bins based on allele frequency (MAF < 0.01, 0.01 < MAF < 0.05, MAF > 0.05). Genomic Relationship Matrix (GRM; 12 random genetic effects) were constructed from SNPs annotated to the 12 different bins. To estimate SNP-based heritability (*h*^*2*^_SNP_) we implemented a GREML-LDMS model in GCTA that fit all 12 GRM together while adjusting by sex and the first 10 PCs^76^. This method is unbiased regardless of the properties of the underlying causal SNPs, therefore allowing us to robustly estimate *h*^*2*^_SNP_ explained by bins of SNPs at different MAF while correcting for the region-specific LD heterogeneity across the genome^76^. Then, 2,663 significant variants (*P* < 1 × 10^−8^) from the Meta-Analysis as well as all SNPs within a window of ±500 kb were selected as significant loci and removed from the 12 bins annotated previously. 12 new GRM were constructed from these 12 bins of which a total of 82,621 SNPs were excluded. Estimations of *h*^*2*^_SNP_ fitting components with and without the significant loci were compared to assess the *h*^*2*^_SNP_ explained by significant loci identified in our meta-analysis. The estimates of variance explained on the observed scale were transformed to that on the underlying scale considering a population prevalence of 10%.

### Reporting summary

Further information on research design is available in the Nature Research Reporting Summary linked to this article.

## Supplementary information

Supplementary Information

Description of Additional Supplementary Files

Supplementary Data 1–14

Reporting Summary

## Data Availability

Summary statistics of the meta-analysis which were generated in this study have been deposited in the GWAS Catalog (https://www.ebi.ac.uk/gwas/home) under study accession GCST90044763.
