# Supplementary material for: Rare variant analysis in eczema identifies exonic variants in DUSP1, NOTCH4 and SLC9A4
Source: Nat Commun. 2021 Nov 16;12:6618. doi: 10.1038/s41467-021-26783-x (PMC8595373; doi:10.1038/s41467-021-26783-x)
Supplement: Supplementary file 1 — Supplementary Information [file 41467_2021_26783_MOESM1_ESM.pdf]

Supplementary Information

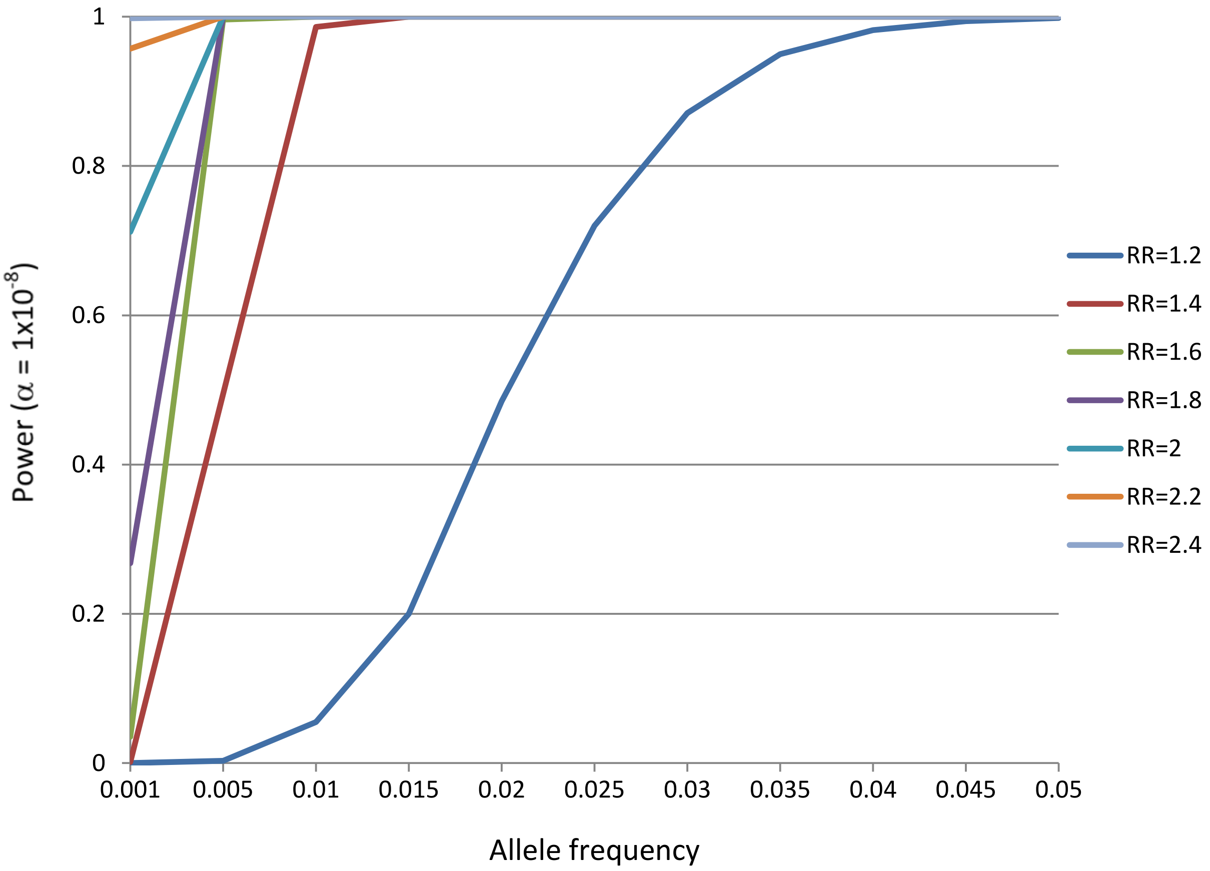

Supplementary Figure 1. Study power for different allele frequencies and effect sizes.
The power to find variants at genome-wide significance dependent on the allele frequency is shown for a data set of 20,016 cases and 380,433 controls. Colors indicate the correlation between power and effect size (relative risk, RR). Power was calculated with the genetic power calculator (https://zzz.bwh.harvard.edu/gpc/).[1]

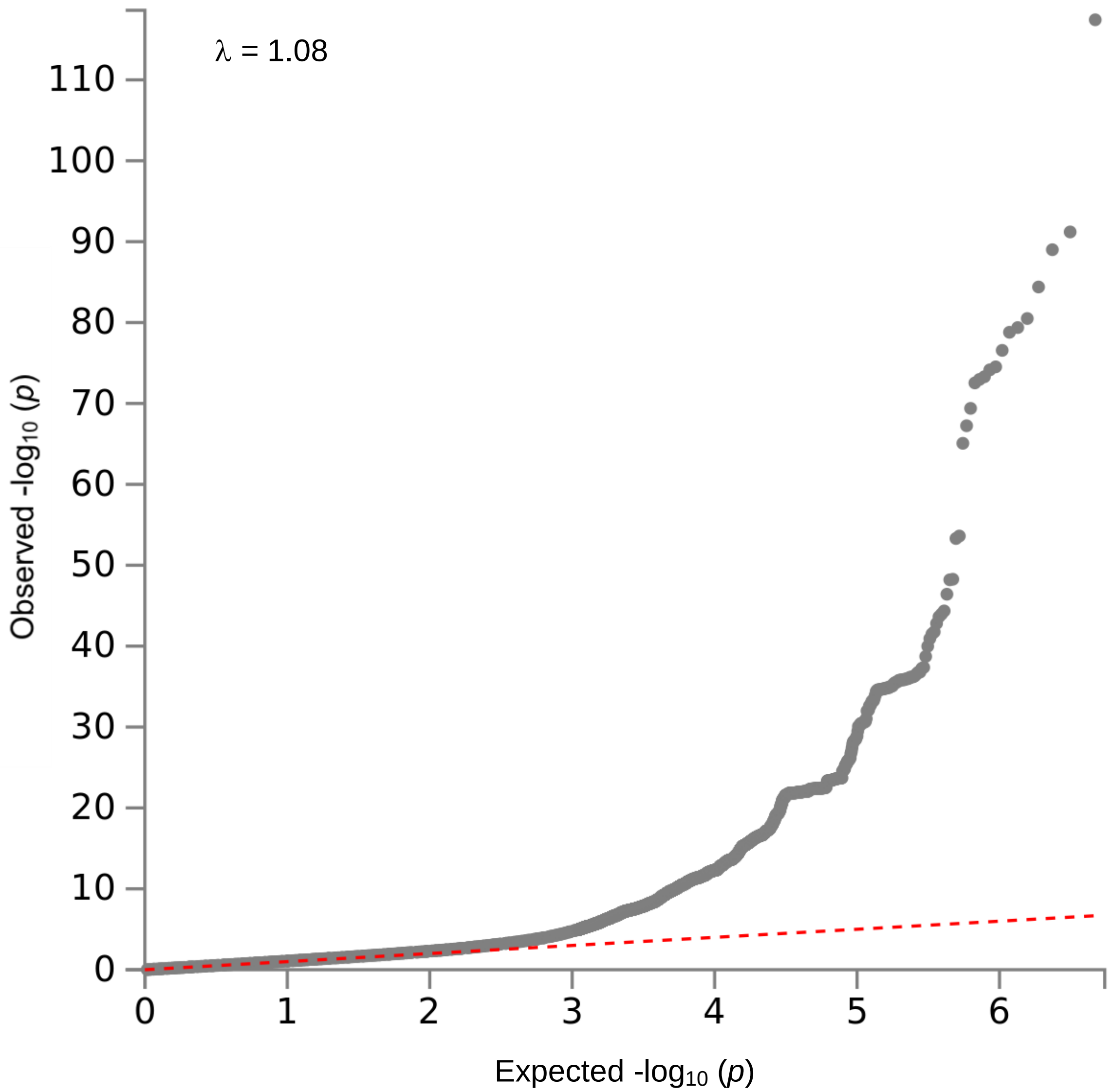

Supplementary Figure 2. Quantile-quantile plot showing the distribution of observed *P* values (*x*-axis) and expected *P* values (*y*-axis) for the meta-GWAS on rare variants in eczema. The expected -$\log_{10}$ *P* values are depicted on the x-axis. The red line corresponds to a correlation of 1 (no inflation of the test statistics). The genomic inflation factor lambda is indicated.

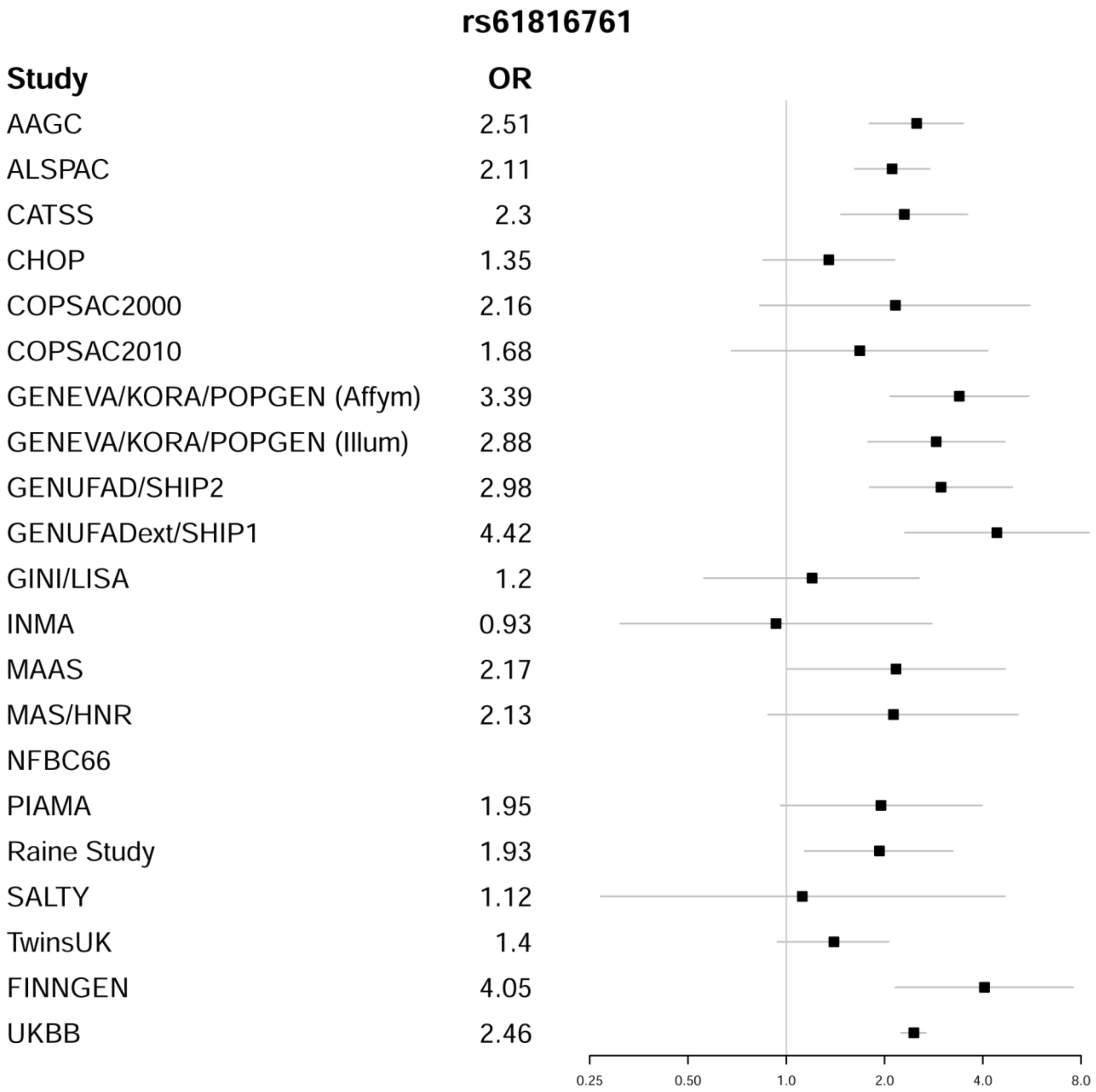

Supplementary Figure 3. Risk estimates of the *FLG* loss-of-function variant rs61816761 in the study populations of RV set, FINNGEN, and UKBB.

Odds ratios (OR, squares) and 95% confidence intervals (error bars) are shown. rs61816761 was not available in NFBC66. Numbers of cases/controls for each study; AAGC, 934/2101 ; ALSPAC, 1633/3600; CATSS, 873/5306; CHOP, 624/1774; COPSAC2000, 73/263; COPSAC2010, 177/441; GENEVA/KORA F4/POPGEN (Affym), 517/1304; GENEVA/KORA F4/POPGEN (Illum), 529/1247; GENUFADext/SHIP1, 417/1667; GENUFAD/SHIP2, 259/1792; GINI/LISA, 442/865; INMA, 404/440; MAAS, 257/355; MAS/HNR, 104/379; PIAMA, 808/895; Raine Study, 404/972; SALTY, 103/2254; TWINS UK, 831/2044; FINNGEN, 2663/88760; UKBB, 6650/260828.

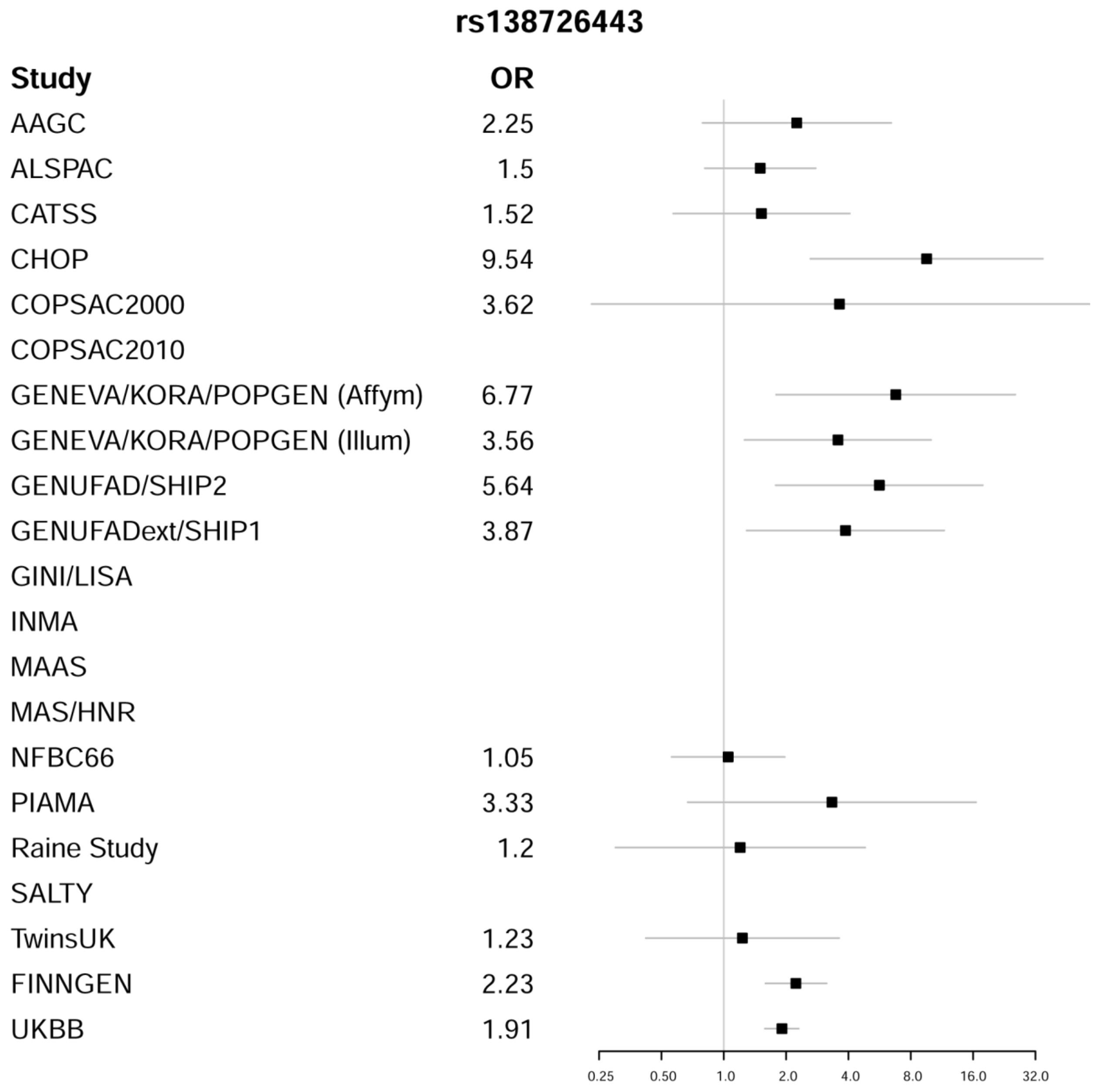

Supplementary Figure 4. Risk estimates of the *FLG* loss-of-function variant rs138726443 in the study populations of RV set, FINNGEN, and UKBB.

Odds ratios (OR, squares) and 95% confidence intervals (error bars) are shown. rs138726443 was not available in COPSAC2010, GINI/LISA, INMA, MAAS, MAS/HNR, and SALTY. Numbers of cases/controls for each study; AAGC, 934/2101 ; ALSPAC, 1633/3600; CATSS, 873/5306; CHOP, 624/1774; COPSAC2000, 73/263; GENEVA/KORA F4/POPGEN (Affym), 517/1304; GENEVA/KORA F4/POPGEN (Illum), 529/1247; GENUFADext/SHIP1, 417/1667; GENUFAD/SHIP2, 259/1792; NFBC66, 1314/3146; PIAMA, 808/895; Raine Study, 404/972; TWINS UK, 831/2044; FINNGEN, 2663/88760; UKBB, 6650/260828.

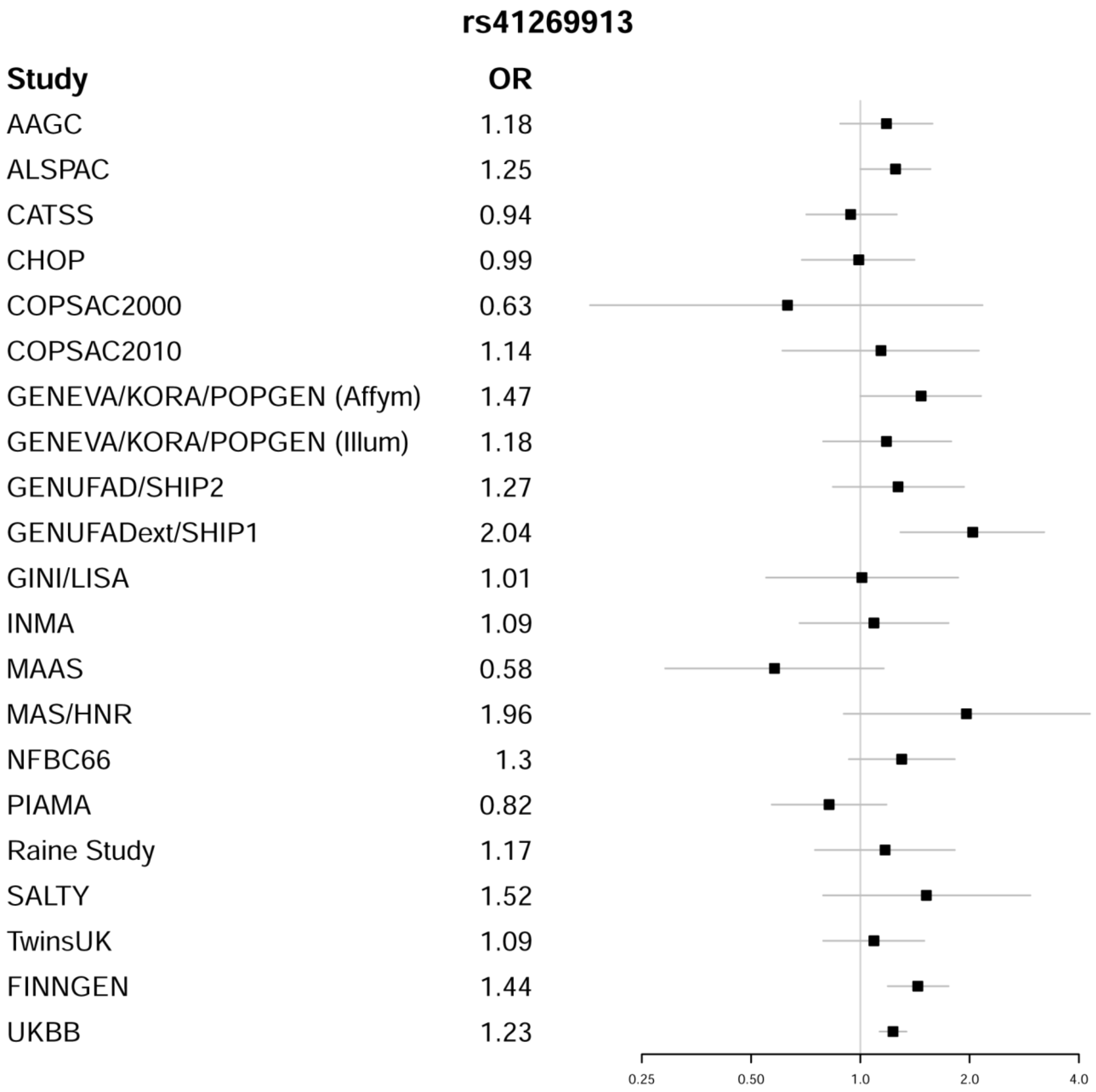

Supplementary Figure 5. Risk estimates of rs41269913 in *SHE* in the study populations of RV set, FINNGEN, and UKBB.

Odds ratios (OR, squares) and 95% confidence intervals (error bars) are shown. Numbers of cases/controls for each study; AAGC, 934/2101 ; ALSPAC, 1633/3600; CATSS, 873/5306; CHOP, 624/1774; COPSAC2000, 73/263; COPSAC2010, 177/441; GENEVA/KORA F4/POPGEN (Affym), 517/1304; GENEVA/KORA F4/POPGEN (Illum), 529/1247; GENUFADext/SHIP1, 417/1667; GENUFAD/SHIP2, 259/1792; GINI/LISA, 442/865; INMA, 404/440; MAAS, 257/355; MAS/HNR, 104/379; NFBC66, 1314/3146; PIAMA, 808/895; Raine Study, 404/972; SALTY, 103/2254; TWINS UK, 831/2044; FINNGEN, 2663/88760; UKBB, 6650/260828.

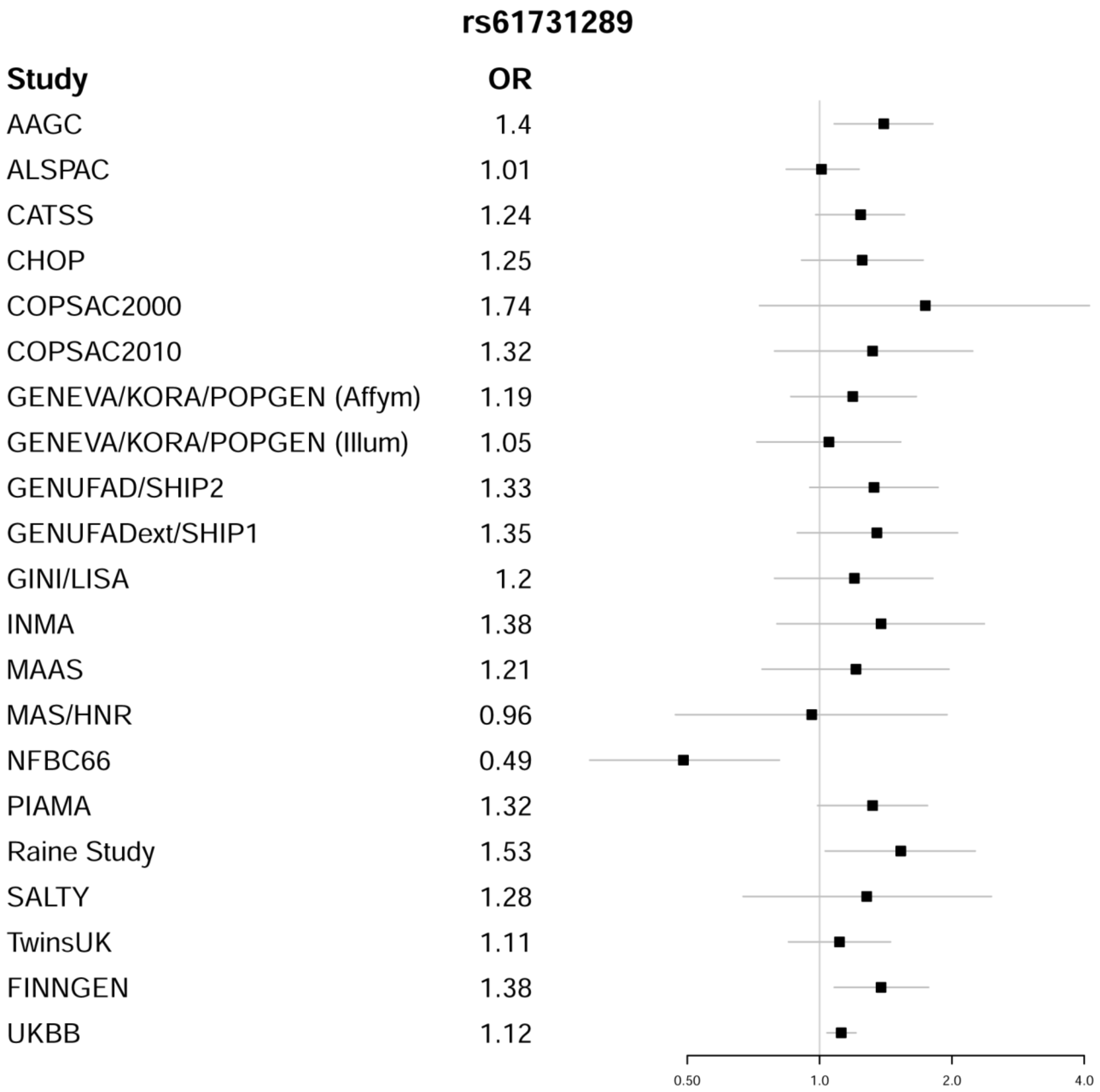

Supplementary Figure 6. Risk estimates of rs61731289 in *SLC9A4* in the study populations of RV set, FINNGEN, and UKBB.

Odds ratios (OR, squares) and 95% confidence intervals (error bars) are shown. Numbers of cases/controls for each study; AAGC, 934/2101 ; ALSPAC, 1633/3600; CATSS, 873/5306; CHOP, 624/1774; COPSAC2000, 73/263; COPSAC2010, 177/441; GENEVA/KORA F4/POPGEN (Affym), 517/1304; GENEVA/KORA F4/POPGEN (Illum), 529/1247; GENUFADext/SHIP1, 417/1667; GENUFAD/SHIP2, 259/1792; GINI/LISA, 442/865; INMA, 404/440; MAAS, 257/355; MAS/HNR, 104/379; NFBC66, 1314/3146; PIAMA, 808/895; Raine Study, 404/972; SALTY, 103/2254; TWINS UK, 831/2044; FINNGEN, 2663/88760; UKBB, 6650/260828.

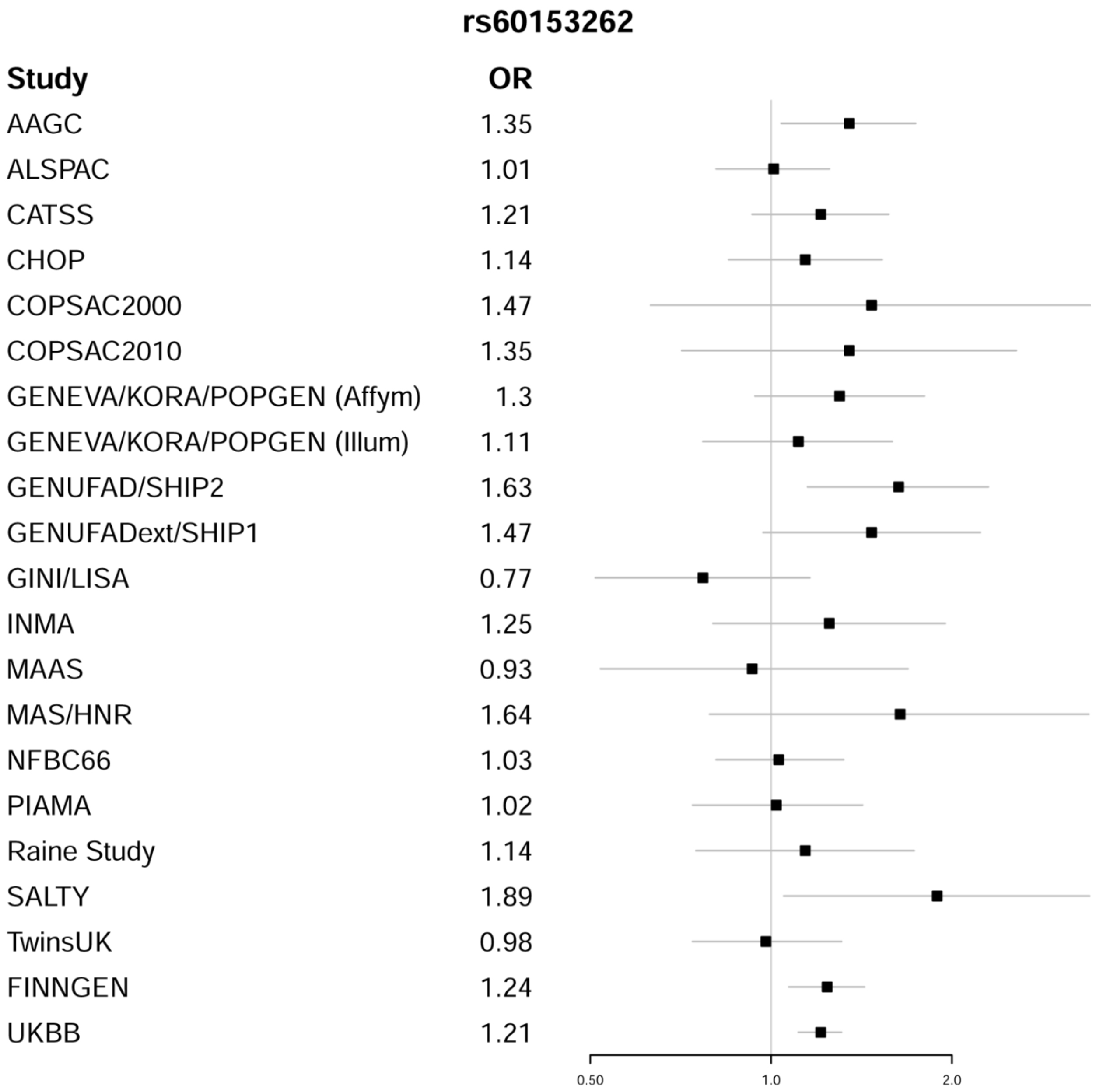

Supplementary Figure 7. Risk estimates of rs60153262 in *TH2LCRR* in the study populations of RV set, FINNGEN, and UKBB.

Odds ratios (OR, squares) and 95% confidence intervals (error bars) are shown. Numbers of cases/controls for each study; AAGC, 934/2101 ; ALSPAC, 1633/3600; CATSS, 873/5306; CHOP, 624/1774; COPSAC2000, 73/263; COPSAC2010, 177/441; GENEVA/KORA F4/POPGEN (Affym), 517/1304; GENEVA/KORA F4/POPGEN (Illum), 529/1247; GENUFADext/SHIP1, 417/1667; GENUFAD/SHIP2, 259/1792; GINI/LISA, 442/865; INMA, 404/440; MAAS, 257/355; MAS/HNR, 104/379; NFBC66, 1314/3146; PIAMA, 808/895; Raine Study, 404/972; SALTY, 103/2254; TWINS UK, 831/2044; FINNGEN, 2663/88760; UKBB, 6650/260828.

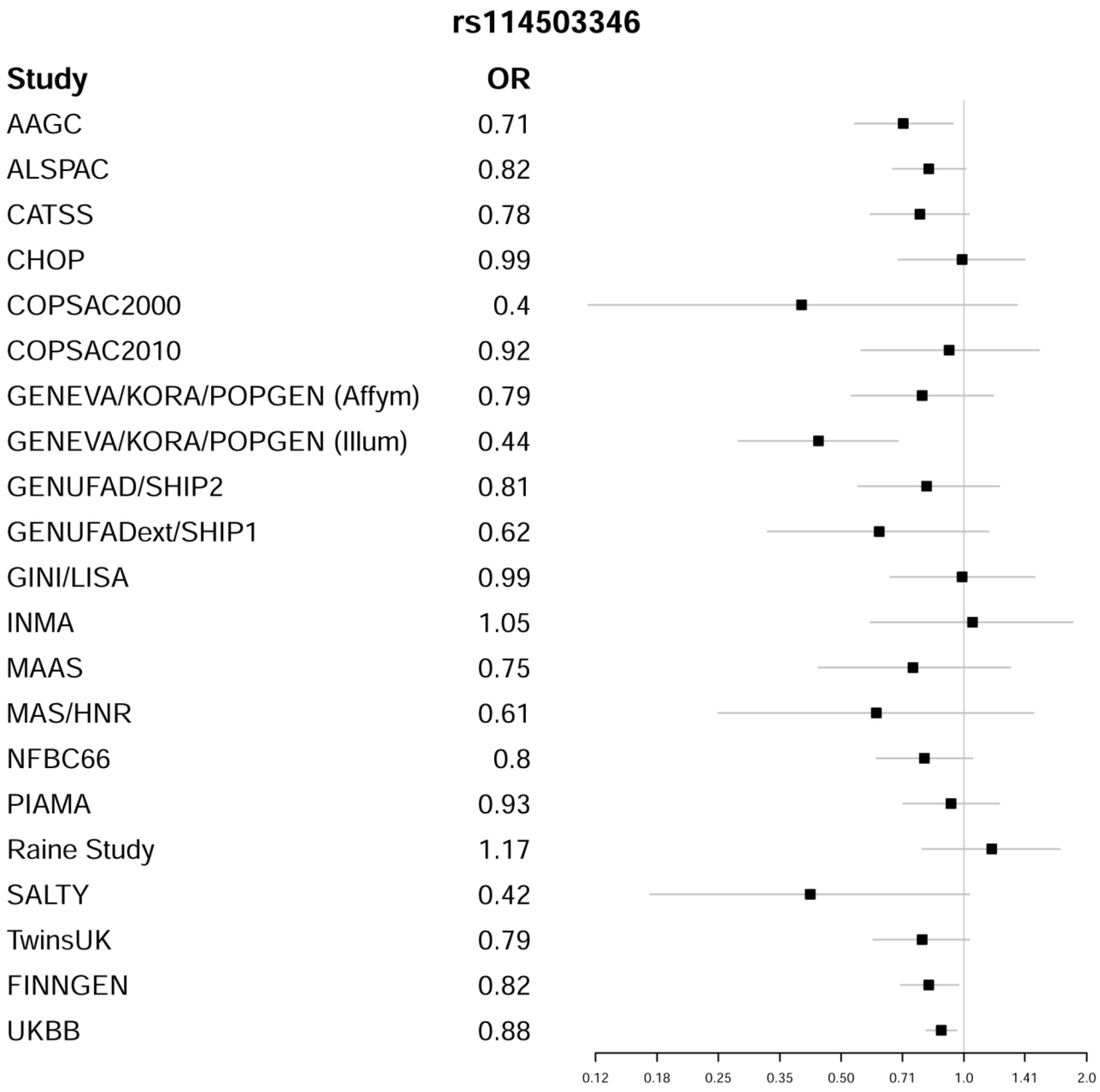

Supplementary Figure 8. Risk estimates of rs114503346 near *DUSP1* in the study populations of RV set, FINNGEN, and UKBB.
Odds ratios (OR, squares) and 95% confidence intervals (error bars) are shown. Numbers of cases/controls for each study; AAGC, 934/2101 ; ALSPAC, 1633/3600; CATSS, 873/5306; CHOP, 624/1774; COPSAC2000, 73/263; COPSAC2010, 177/441; GENEVA/KORA F4/POPGEN (Affym), 517/1304; GENEVA/KORA F4/POPGEN (Illum), 529/1247; GENUFADext/SHIP1, 417/1667; GENUFAD/SHIP2, 259/1792; GINI/LISA, 442/865; INMA, 404/440; MAAS, 257/355; MAS/HNR, 104/379; NFBC66, 1314/3146; PIAMA, 808/895; Raine Study, 404/972; SALTY, 103/2254; TWINS UK, 831/2044; FINNGEN, 2663/88760; UKBB, 6650/260828.

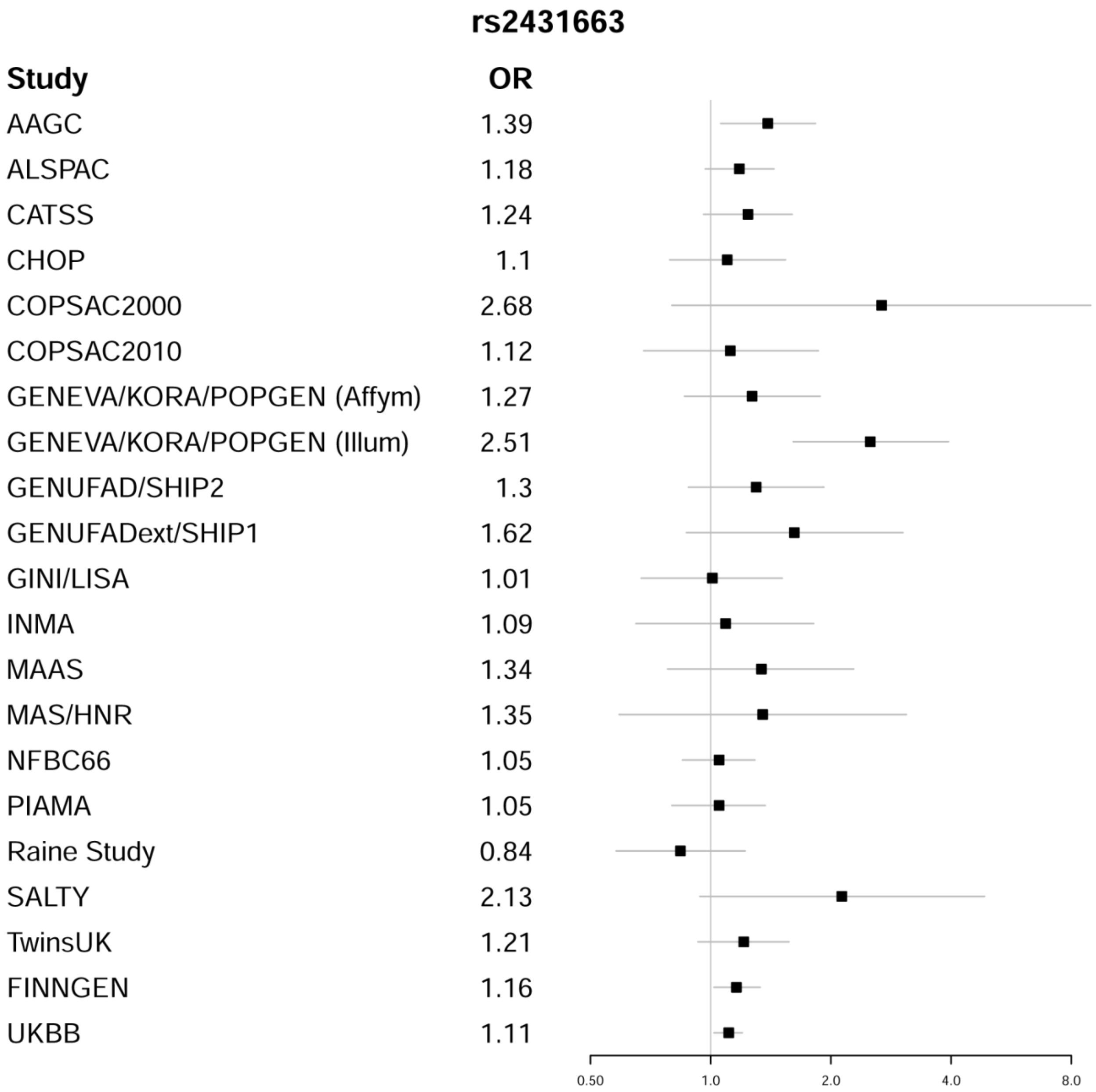

Supplementary Figure 9. Risk estimates of rs2431663 in *DUSP1* in the study populations of RV set, FINNGEN, and UKBB.

Odds ratios (OR, squares) and 95% confidence intervals (error bars) are shown. Numbers of cases/controls for each study; AAGC, 934/2101 ; ALSPAC, 1633/3600; CATSS, 873/5306; CHOP, 624/1774; COPSAC2000, 73/263; COPSAC2010, 177/441; GENEVA/KORA F4/POPGEN (Affym), 517/1304; GENEVA/KORA F4/POPGEN (Illum), 529/1247; GENUFADext/SHIP1, 417/1667; GENUFAD/SHIP2, 259/1792; GINI/LISA, 442/865; INMA, 404/440; MAAS, 257/355; MAS/HNR, 104/379; NFBC66, 1314/3146; PIAMA, 808/895; Raine Study, 404/972; SALTY, 103/2254; TWINS UK, 831/2044; FINNGEN, 2663/88760; UKBB, 6650/260828.

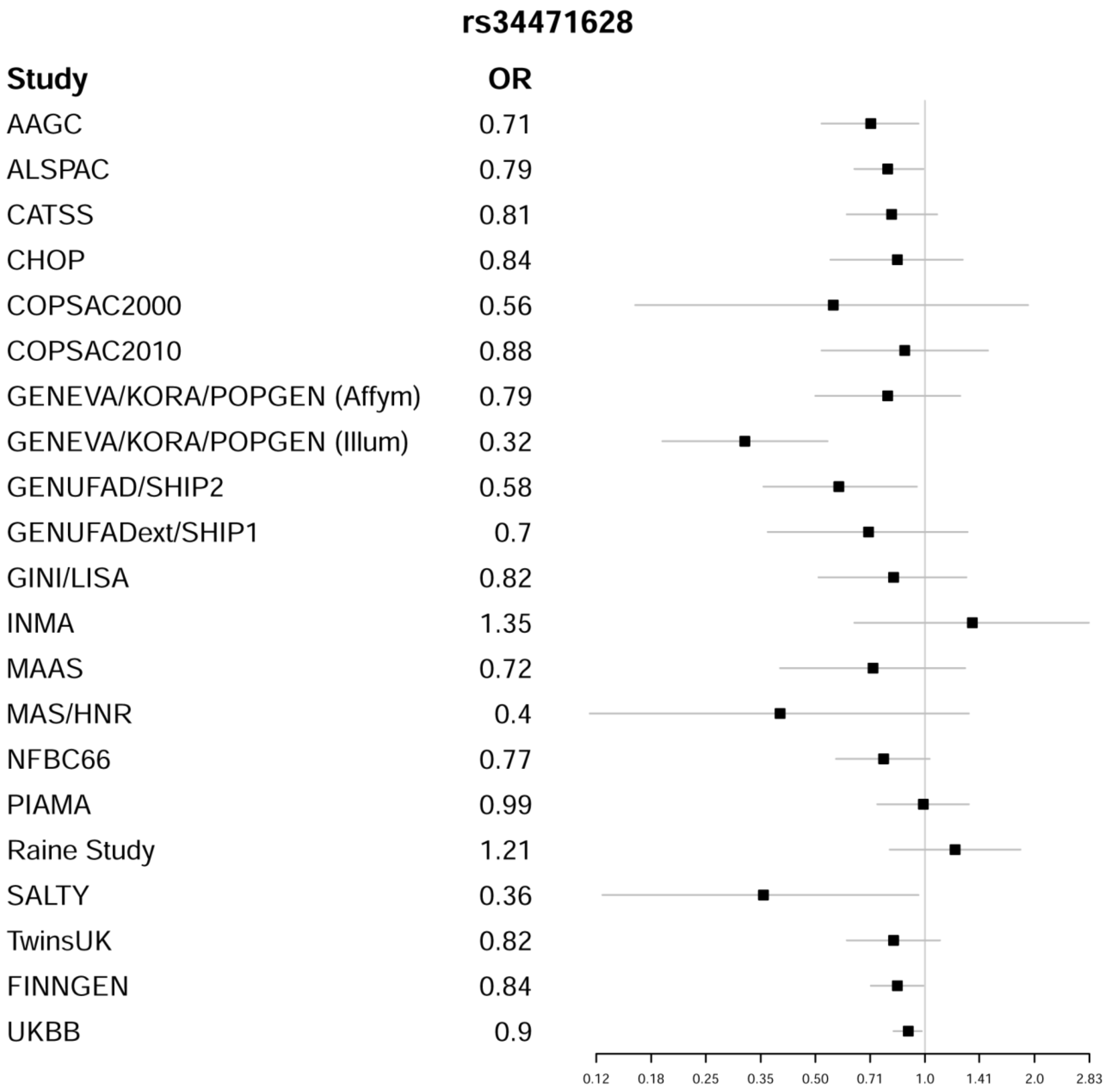

Supplementary Figure 10. Risk estimates of rs34471628 in *DUSP1* in the study populations of RV set, FINNGEN, and UKBB.

Odds ratios (OR, squares) and 95% confidence intervals (error bars) are shown. Numbers of cases/controls for each study; AAGC, 934/2101 ; ALSPAC, 1633/3600; CATSS, 873/5306; CHOP, 624/1774; COPSAC2000, 73/263; COPSAC2010, 177/441; GENEVA/KORA F4/POPGEN (Affym), 517/1304; GENEVA/KORA F4/POPGEN (Illum), 529/1247; GENUFADext/SHIP1, 417/1667; GENUFAD/SHIP2, 259/1792; GINI/LISA, 442/865; INMA, 404/440; MAAS, 257/355; MAS/HNR, 104/379; NFBC66, 1314/3146; PIAMA, 808/895; Raine Study, 404/972; SALTY, 103/2254; TWINS UK, 831/2044; FINNGEN, 2663/88760; UKBB, 6650/260828.

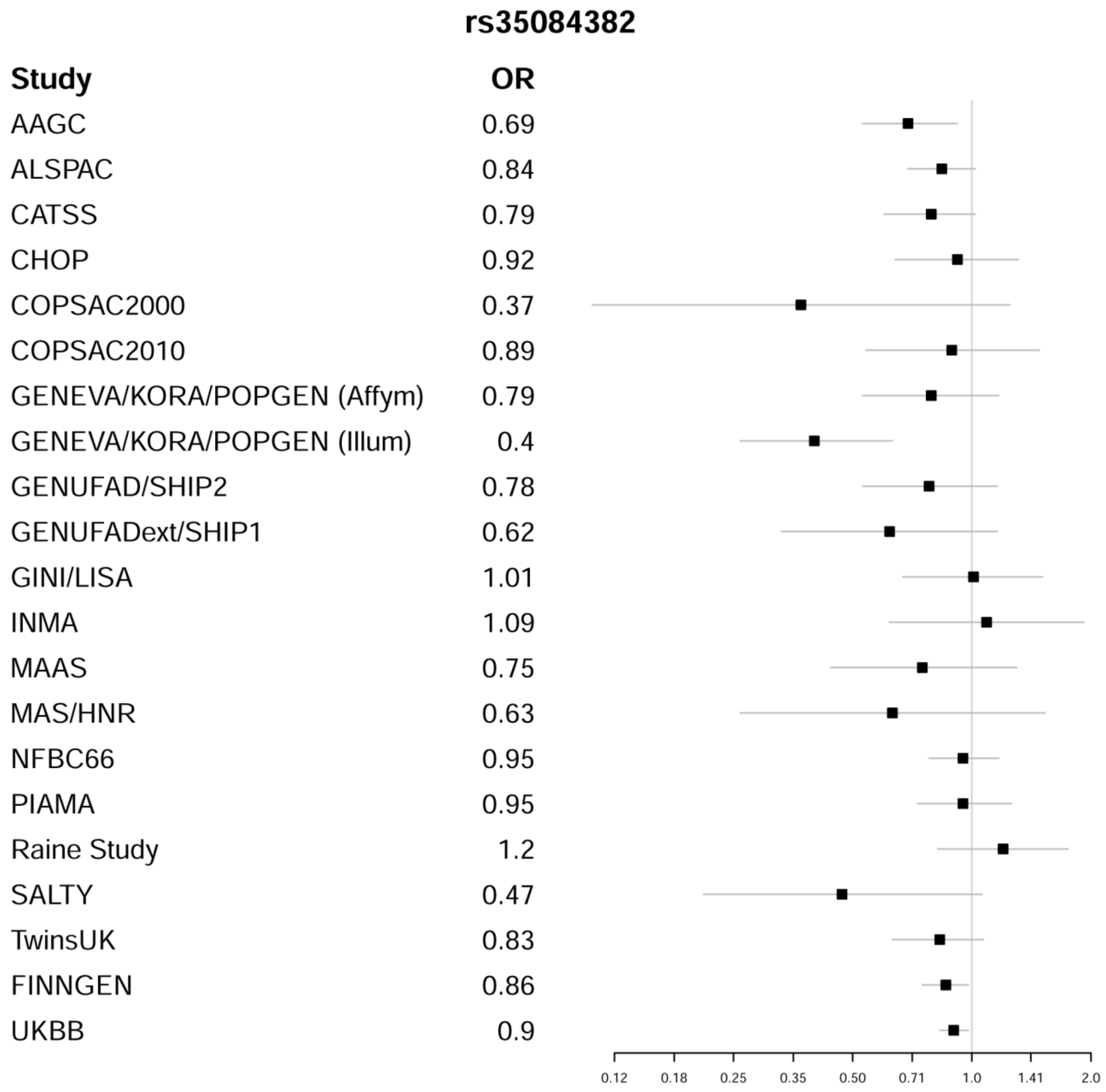

Supplementary Figure 11. Risk estimates of rs35084382 in *DUSP1* in the study populations of RV set, FINNGEN, and UKBB.
Odds ratios (OR, squares) and 95% confidence intervals (error bars) are shown. Numbers of cases/controls for each study; AAGC, 934/2101 ; ALSPAC, 1633/3600; CATSS, 873/5306; CHOP, 624/1774; COPSAC2000, 73/263; COPSAC2010, 177/441; GENEVA/KORA F4/POPGEN (Affym), 517/1304; GENEVA/KORA F4/POPGEN (Illum), 529/1247; GENUFADext/SHIP1, 417/1667; GENUFAD/SHIP2, 259/1792; GINI/LISA, 442/865; INMA, 404/440; MAAS, 257/355; MAS/HNR, 104/379; NFBC66, 1314/3146; PIAMA, 808/895; Raine Study, 404/972; SALTY, 103/2254; TWINS UK, 831/2044; FINNGEN, 2663/88760; UKBB, 6650/260828.

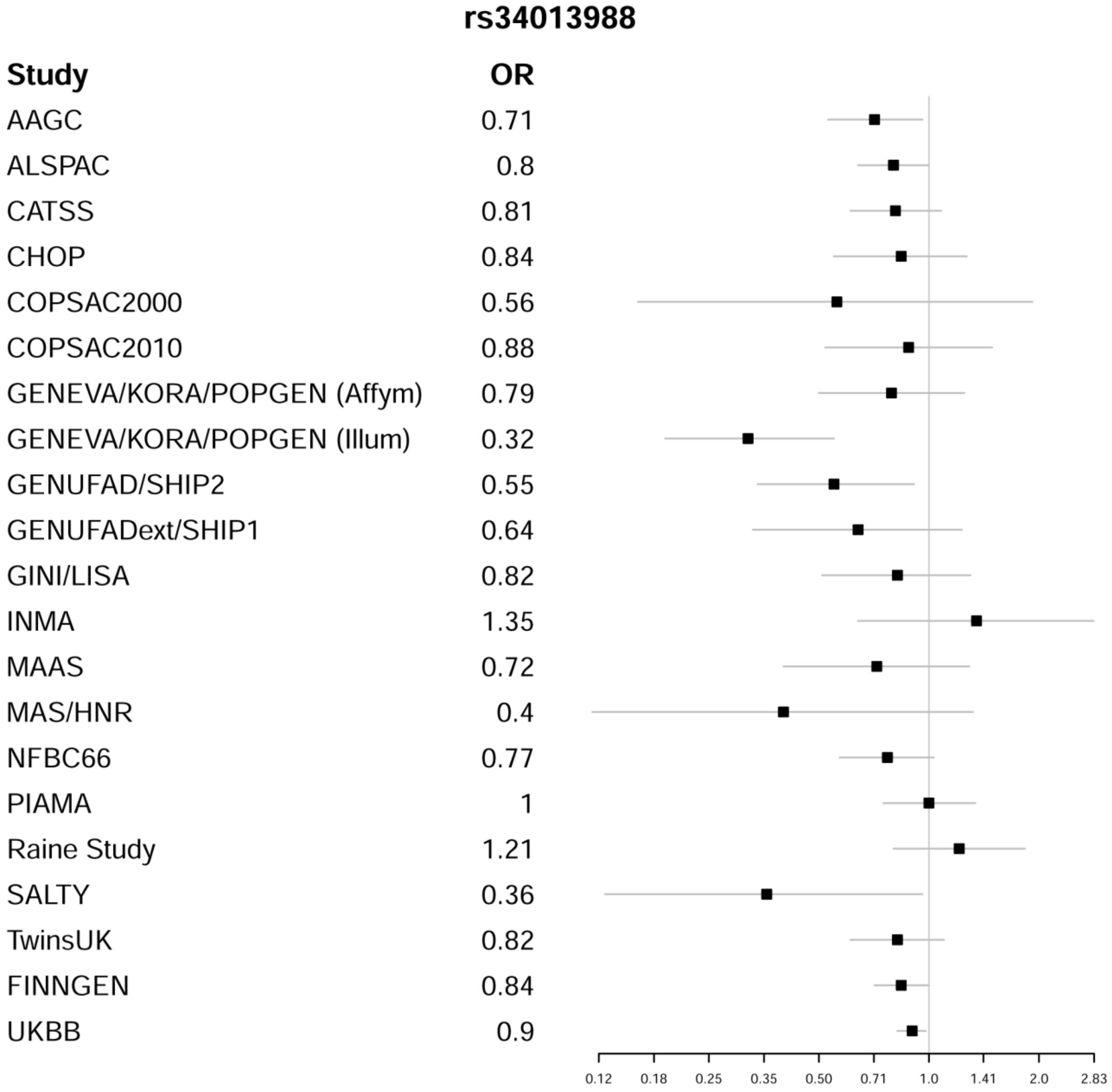

Supplementary Figure 12. Risk estimates of rs34013988 in *DUSP1* in the study populations of RV set, FINNGEN, and UKBB.
Odds ratios (OR, squares) and 95% confidence intervals (error bars) are shown. Numbers of cases/controls for each study; AAGC, 934/2101 ; ALSPAC, 1633/3600; CATSS, 873/5306; CHOP, 624/1774; COPSAC2000, 73/263; COPSAC2010, 177/441; GENEVA/KORA F4/POPGEN (Affym), 517/1304; GENEVA/KORA F4/POPGEN (Illum), 529/1247; GENUFADext/SHIP1, 417/1667; GENUFAD/SHIP2, 259/1792; GINI/LISA, 442/865; INMA, 404/440; MAAS, 257/355; MAS/HNR, 104/379; NFBC66, 1314/3146; PIAMA, 808/895; Raine Study, 404/972; SALTY, 103/2254; TWINS UK, 831/2044; FINNGEN, 2663/88760; UKBB, 6650/260828.

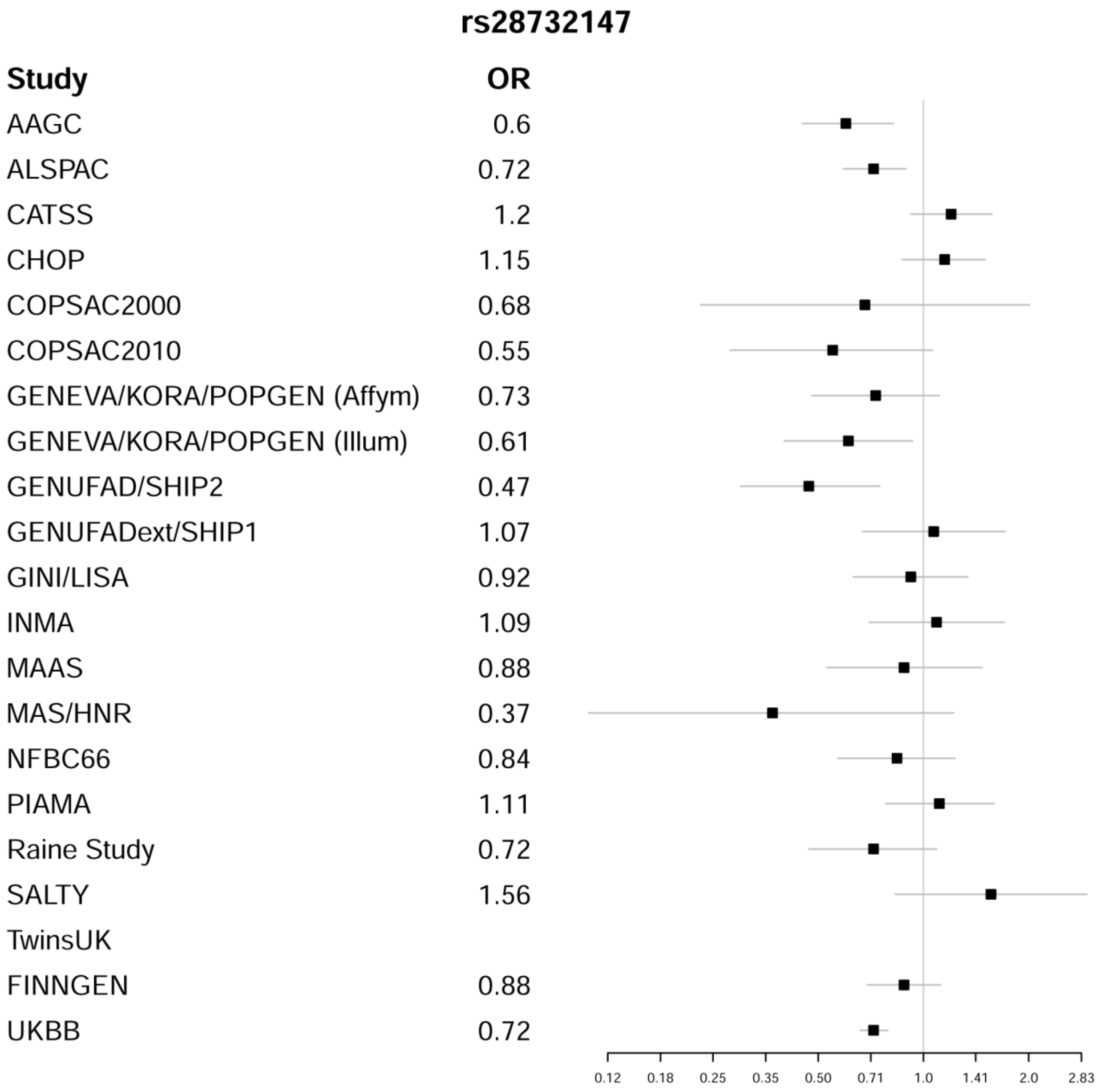

Supplementary Figure 13. Risk estimates of rs28732147 near*NCR3/AIF1* in the study populations of RV set, FINNGEN, and UKBB.
Odds ratios (OR, squares) and 95% confidence intervals (error bars) are shown. rs28732147 was not available in TwinsUK. Numbers of cases/controls for each study; AAGC, 934/2101 ; ALSPAC, 1633/3600; CATSS, 873/5306; CHOP, 624/1774; COPSAC2000, 73/263; COPSAC2010, 177/441; GENEVA/KORA F4/POPGEN (Affym), 517/1304; GENEVA/KORA F4/POPGEN (Illum), 529/1247; GENUFADext/SHIP1, 417/1667; GENUFAD/SHIP2, 259/1792; GINI/LISA, 442/865; INMA, 404/440; MAAS, 257/355; MAS/HNR, 104/379; NFBC66, 1314/3146; PIAMA, 808/895; Raine Study, 404/972; SALTY, 103/2254; FINNGEN, 2663/88760; UKBB, 6650/260828.

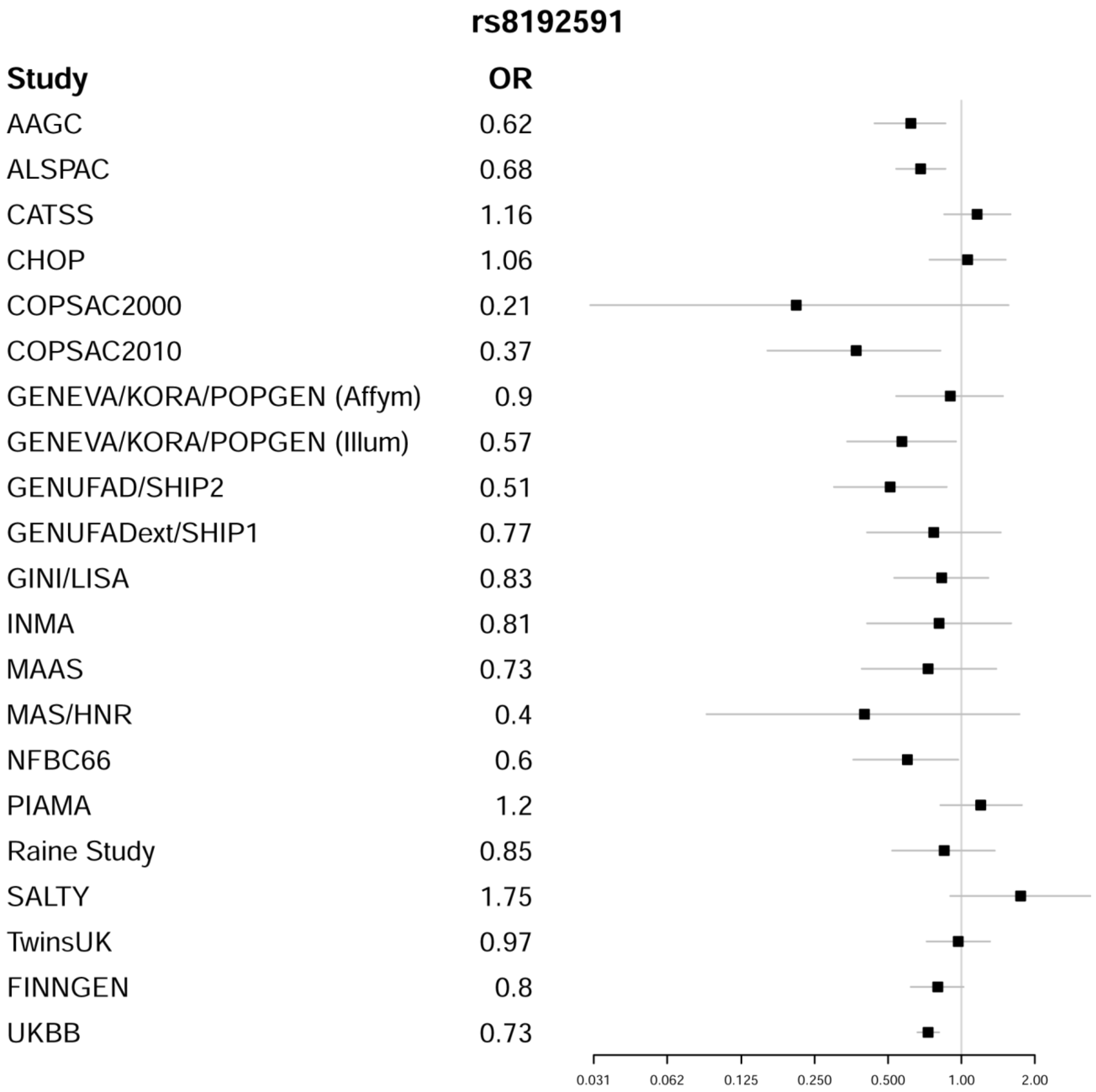

Supplementary Figure 14. Risk estimates of the *NOTCH4* missense variant rs8192591 in the study populations of RV set, FINNGEN, and UKBB.

Odds ratios (OR, squares) and 95% confidence intervals (error bars) are shown. Numbers of cases/controls for each study; AAGC, 934/2101 ; ALSPAC, 1633/3600; CATSS, 873/5306; CHOP, 624/1774; COPSAC2000, 73/263; COPSAC2010, 177/441; GENEVA/KORA F4/POPGEN (Affym), 517/1304; GENEVA/KORA F4/POPGEN (Illum), 529/1247; GENUFADext/SHIP1, 417/1667; GENUFAD/SHIP2, 259/1792; GINI/LISA, 442/865; INMA, 404/440; MAAS, 257/355; MAS/HNR, 104/379; NFBC66, 1314/3146; PIAMA, 808/895; Raine Study, 404/972; SALTY, 103/2254; TWINS UK, 831/2044; FINNGEN, 2663/88760; UKBB, 6650/260828.

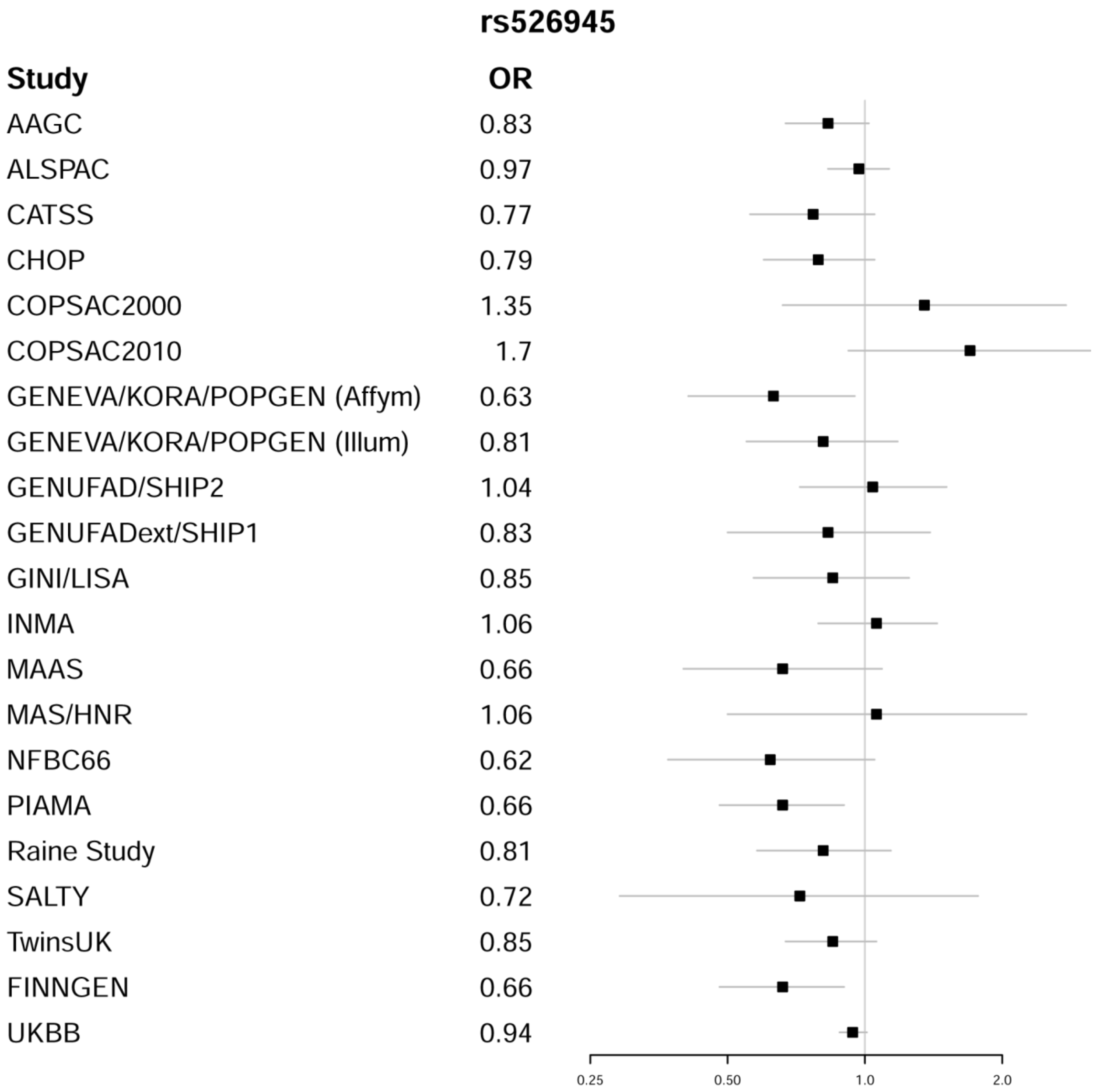

Supplementary Figure 15. Risk estimates of rs526945 in the 5' region of *NOTCH4* in the study populations of RV set, FINNGEN, and UKBB.
Odds ratios (OR, squares) and 95% confidence intervals (error bars) are shown. Numbers of cases/controls for each study; AAGC, 934/2101 ; ALSPAC, 1633/3600; CATSS, 873/5306; CHOP, 624/1774; COPSAC2000, 73/263; COPSAC2010, 177/441; GENEVA/KORA F4/POPGEN (Affym), 517/1304; GENEVA/KORA F4/POPGEN (Illum), 529/1247; GENUFADext/SHIP1, 417/1667; GENUFAD/SHIP2, 259/1792; GINI/LISA, 442/865; INMA, 404/440; MAAS, 257/355; MAS/HNR, 104/379; NFBC66, 1314/3146; PIAMA, 808/895; Raine Study, 404/972; SALTY, 103/2254; TWINS UK, 831/2044; FINNGEN, 2663/88760; UKBB, 6650/260828.

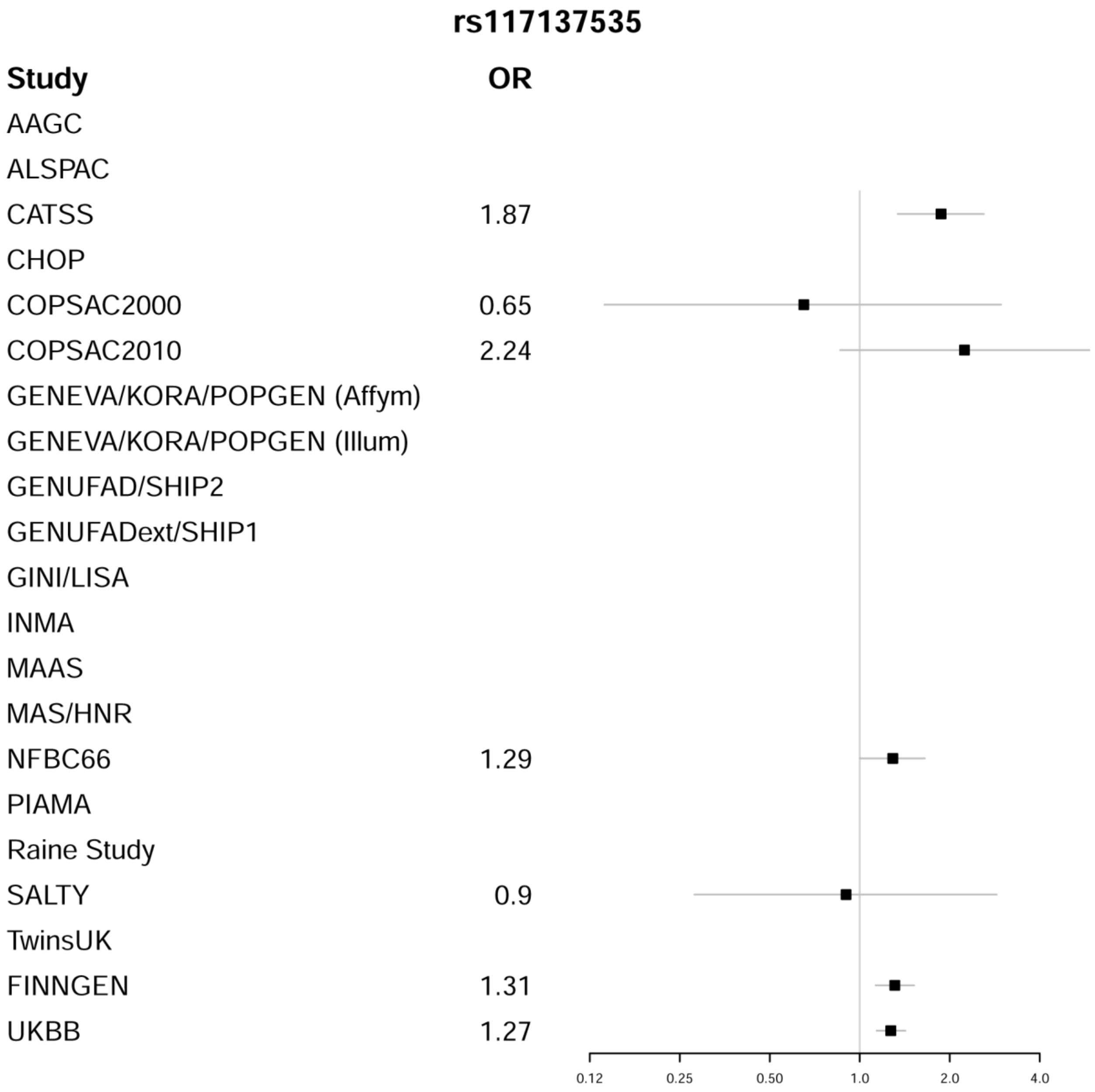

Supplementary Figure 16. Risk estimates of rs117137515 in *ARRDC1* in the study populations of RV set, FINNGEN, and UKBB.

Odds ratios (OR, squares) and 95% confidence intervals (error bars) are shown. rs117137515 was available in CATSS, COPSAC2000, COPSAC2010, NFBC66, SALTY, FINNGEN, and UKBB. Numbers of cases/controls for each study; CATSS, 873/5306; COPSAC2000, 73/263; COPSAC2010, 177/441; NFBC66, 1314/3146; SALTY, 103/2254; FINNGEN, 2663/88760; UKBB, 6650/260828.

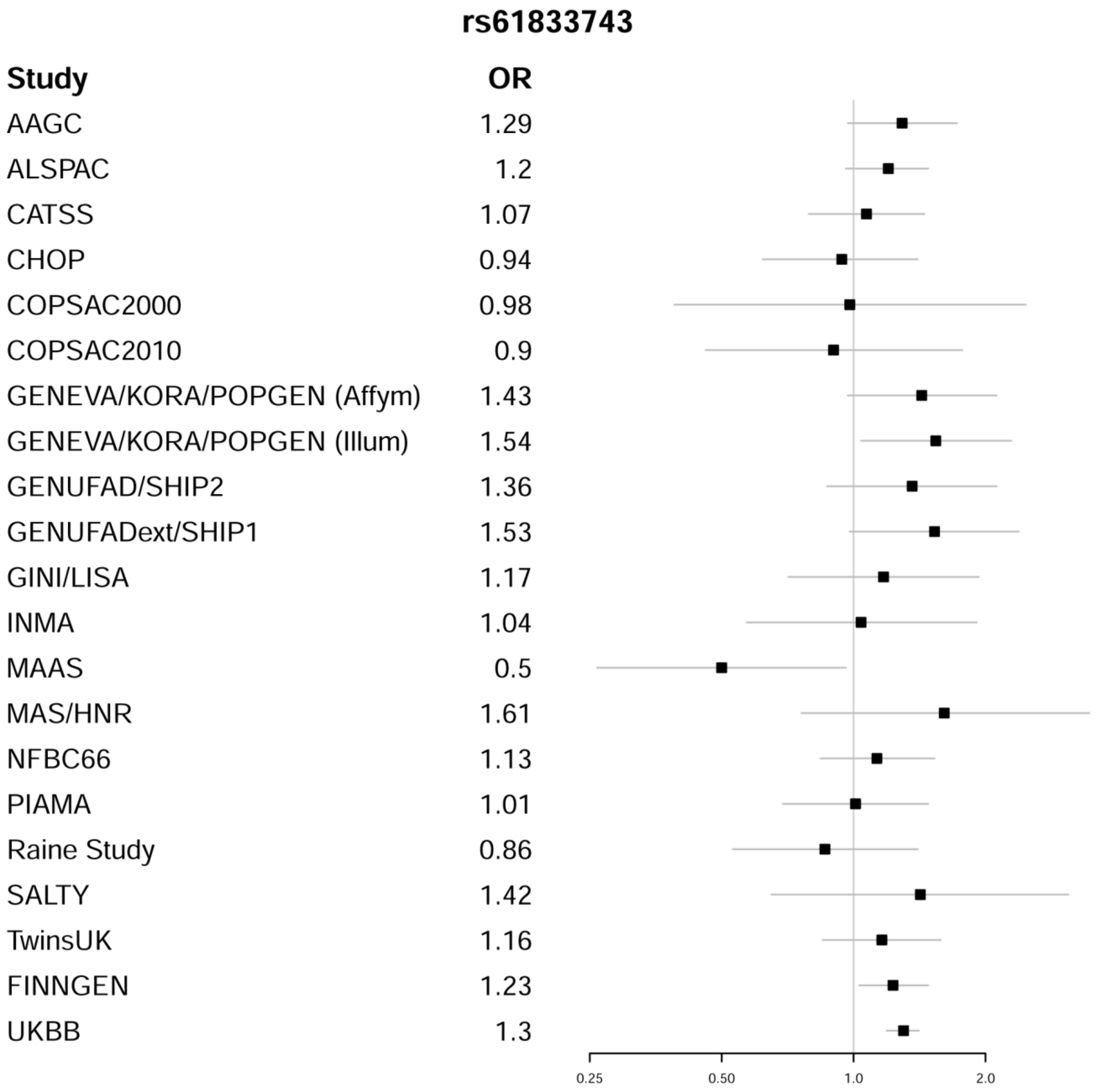

Supplementary Figure 17. Risk estimates of rs61833743 near *IL2RA* in the study populations of RV set, FINNGEN, and UKBB.
Odds ratios (OR, squares) and 95% confidence intervals (error bars) are shown. Numbers of cases/controls for each study; AAGC, 934/2101 ; ALSPAC, 1633/3600; CATSS, 873/5306; CHOP, 624/1774; COPSAC2000, 73/263; COPSAC2010, 177/441; GENEVA/KORA F4/POPGEN (Affym), 517/1304; GENEVA/KORA F4/POPGEN (Illum), 529/1247; GENUFADext/SHIP1, 417/1667; GENUFAD/SHIP2, 259/1792; GINI/LISA, 442/865; INMA, 404/440; MAAS, 257/355; MAS/HNR, 104/379; NFBC66, 1314/3146; PIAMA, 808/895; Raine Study, 404/972; SALTY, 103/2254; TWINS UK, 831/2044; FINNGEN, 2663/88760; UKBB, 6650/260828.

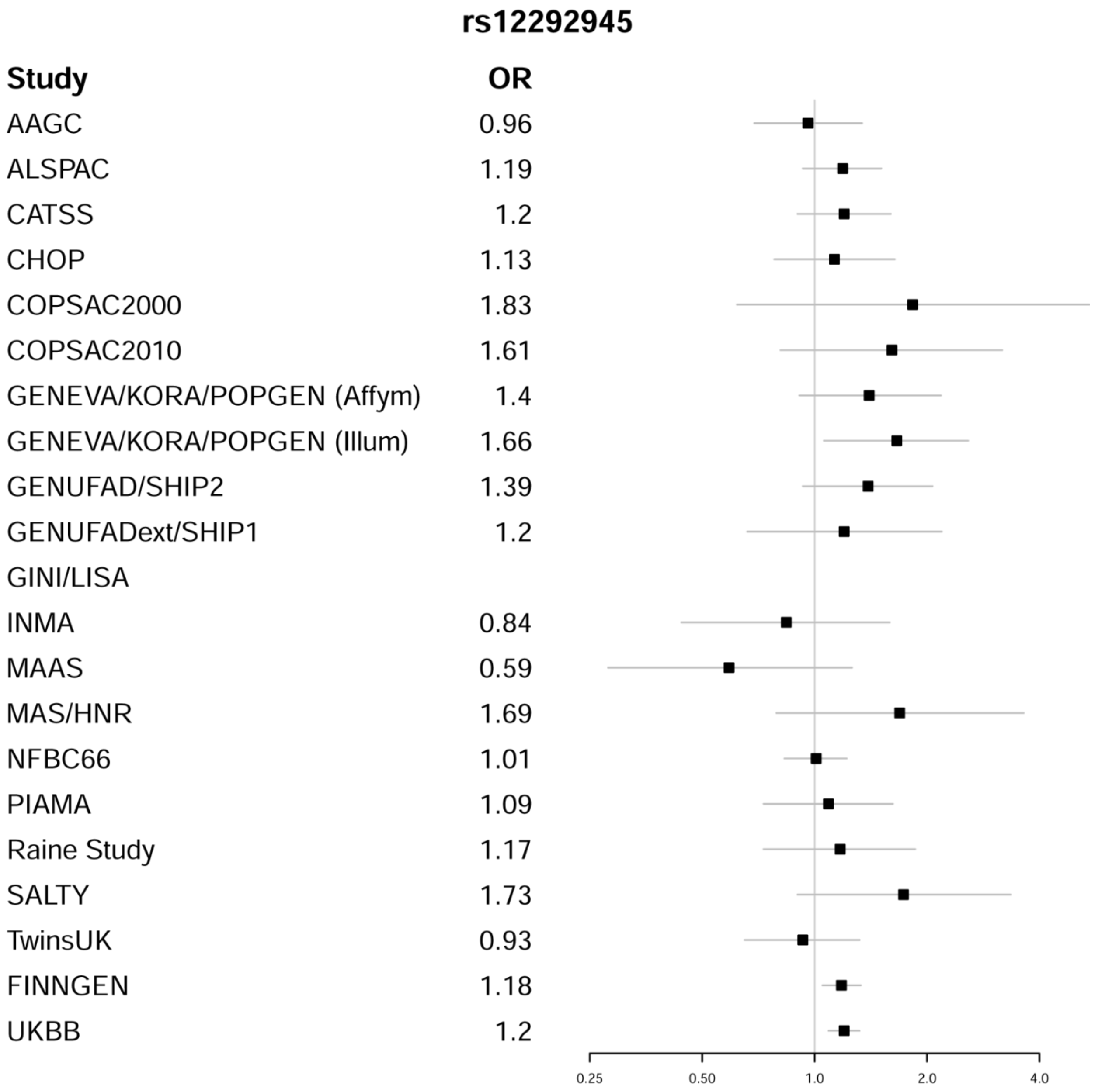

Supplementary Figure 18. Risk estimates of rs12292945 in *PRR5L* in the study populations of RV set, FINNGEN, and UKBB.
Odds ratios (OR, squares) and 95% confidence intervals (error bars) are shown. rs12292945 was not available in GINI/LISA. Numbers of cases/controls for each study; AAGC, 934/2101 ; ALSPAC, 1633/3600; CATSS, 873/5306; CHOP, 624/1774; COPSAC2000, 73/263; COPSAC2010, 177/441; GENEVA/KORA F4/POPGEN (Affym), 517/1304; GENEVA/KORA F4/POPGEN (Illum), 529/1247; GENUFADext/SHIP1, 417/1667; GENUFAD/SHIP2, 259/1792; INMA, 404/440; MAAS, 257/355; MAS/HNR, 104/379; NFBC66, 1314/3146; PIAMA, 808/895; Raine Study, 404/972; SALTY, 103/2254; TWINS UK, 831/2044; FINNGEN, 2663/88760; UKBB, 6650/260828.

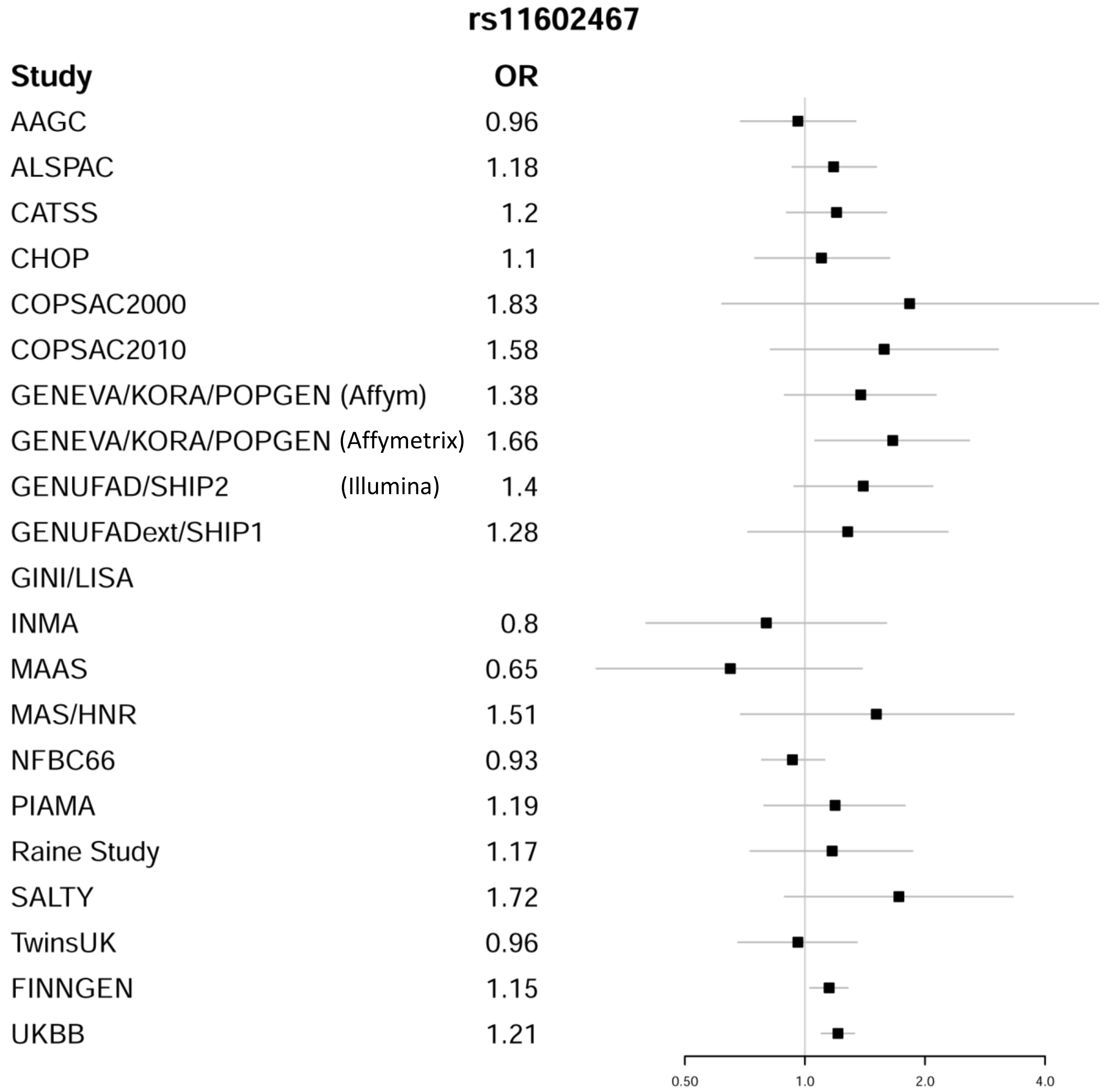

Supplementary Figure 19. Risk estimates of rs11602467 in *PRR5L* in the study populations of RV set, FINNGEN, and UKBB.

Odds ratios (OR, squares) and 95% confidence intervals (error bars) are shown. rs11602467 was not available in GINI/LISA. Numbers of cases/controls for each study; AAGC, 934/2101 ; ALSPAC, 1633/3600; CATSS, 873/5306; CHOP, 624/1774; COPSAC2000, 73/263; COPSAC2010, 177/441; GENEVA/KORA F4/POPGEN (Affym), 517/1304; GENEVA/KORA F4/POPGEN (Illum), 529/1247; GENUFADext/SHIP1, 417/1667; GENUFAD/SHIP2, 259/1792; INMA, 404/440; MAAS, 257/355; MAS/HNR, 104/379; NFBC66, 1314/3146; PIAMA, 808/895; Raine Study, 404/972; SALTY, 103/2254; TWINS UK, 831/2044; FINNGEN, 2663/88760; UKBB, 6650/260828.

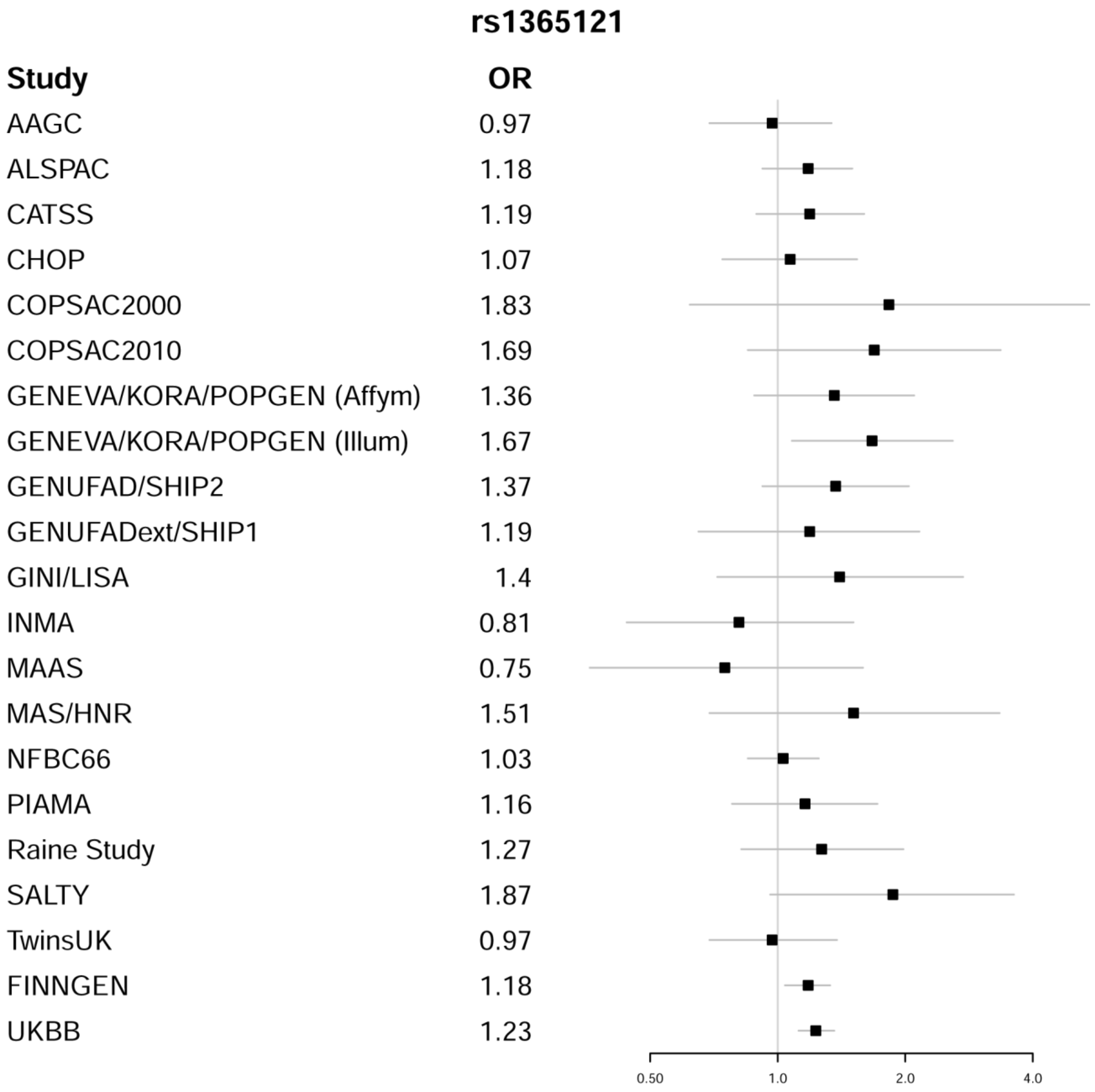

Supplementary Figure 20. Risk estimates of rs1365121 in *PRR5L* in the study populations of RV set, FINNGEN, and UKBB.

Odds ratios (OR, squares) and 95% confidence intervals (error bars) are shown. Numbers of cases/controls for each study; AAGC, 934/2101 ; ALSPAC, 1633/3600; CATSS, 873/5306; CHOP, 624/1774; COPSAC2000, 73/263; COPSAC2010, 177/441; GENEVA/KORA F4/POPGEN (Affym), 517/1304; GENEVA/KORA F4/POPGEN (Illum), 529/1247; GENUFADext/SHIP1, 417/1667; GENUFAD/SHIP2, 259/1792; GINI/LISA, 442/865; INMA, 404/440; MAAS, 257/355; MAS/HNR, 104/379; NFBC66, 1314/3146; PIAMA, 808/895; Raine Study, 404/972; SALTY, 103/2254; TWINS UK, 831/2044; FINNGEN, 2663/88760; UKBB, 6650/260828.

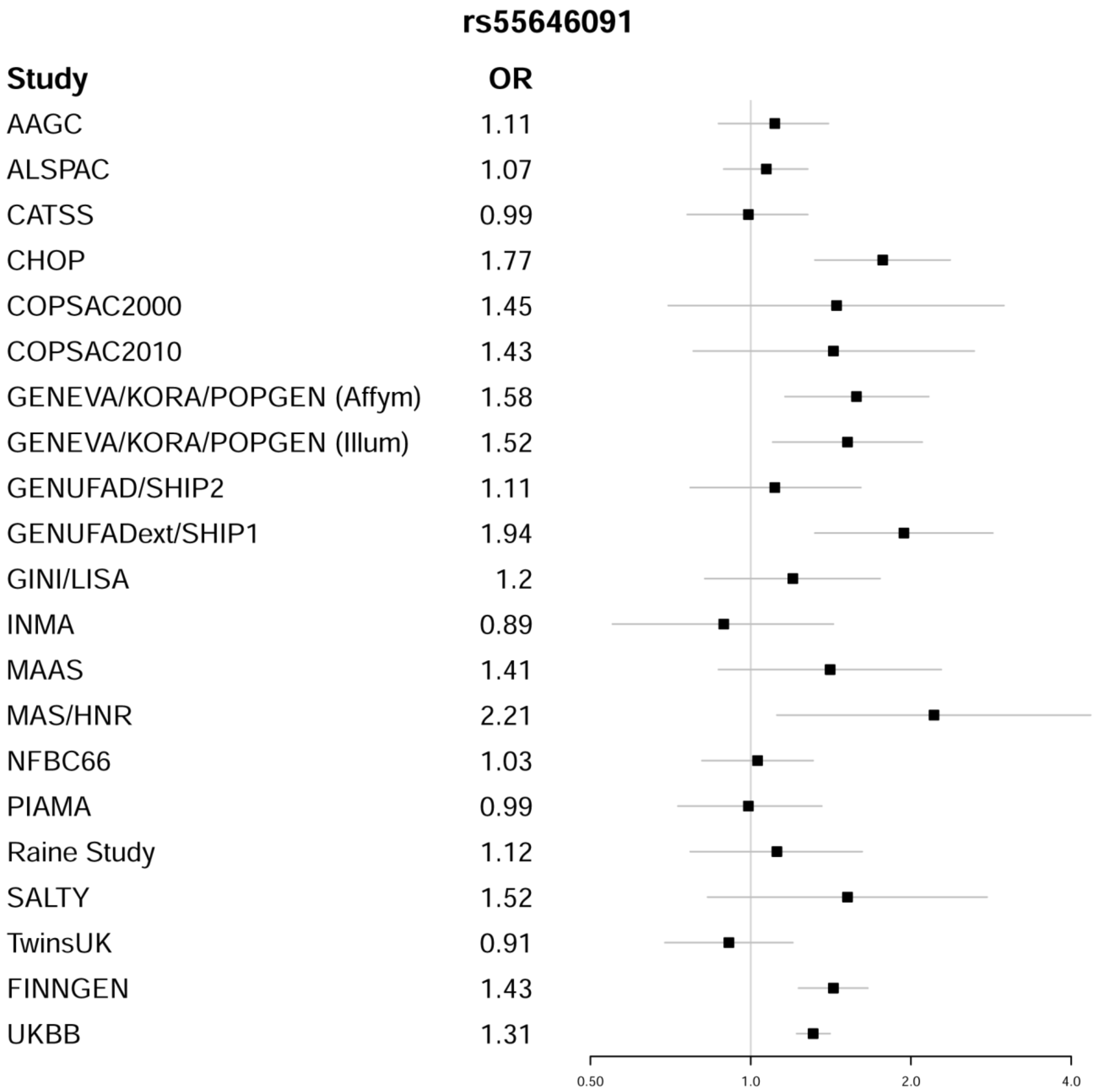

Supplementary Figure 21. Risk estimates of rs55646091 near *LRRC32* in the study populations of RV set, FINNGEN, and UKBB.
Odds ratios (OR, squares) and 95% confidence intervals (error bars) are shown. Numbers of cases/controls for each study; AAGC, 934/2101 ; ALSPAC, 1633/3600; CATSS, 873/5306; CHOP, 624/1774; COPSAC2000, 73/263; COPSAC2010, 177/441; GENEVA/KORA F4/POPGEN (Affym), 517/1304; GENEVA/KORA F4/POPGEN (Illum), 529/1247; GENUFADext/SHIP1, 417/1667; GENUFAD/SHIP2, 259/1792; GINI/LISA, 442/865; INMA, 404/440; MAAS, 257/355; MAS/HNR, 104/379; NFBC66, 1314/3146; PIAMA, 808/895; Raine Study, 404/972; SALTY, 103/2254; TWINS UK, 831/2044; FINNGEN, 2663/88760; UKBB, 6650/260828.

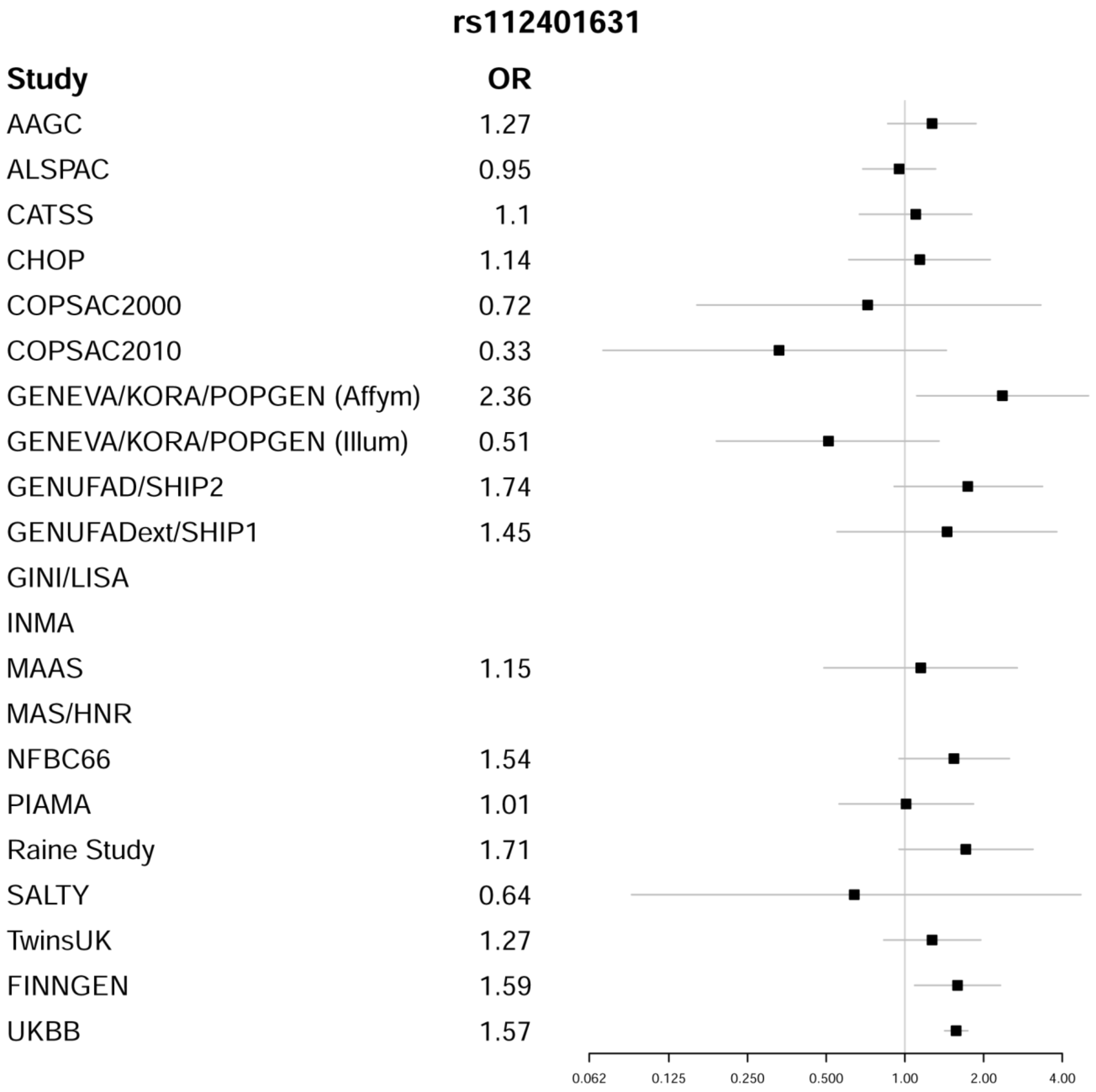

Supplementary Figure 22. Risk estimates of rs112401631 in *SMARCE1* in the study populations of RV set, FINNGEN, and UKBB.

Odds ratios (OR, squares) and 95% confidence intervals (error bars) are shown. rs112401631 was not available in GINI/LISA, INMA, and MAS/HNR. Numbers of cases/controls for each study; AAGC, 934/2101 ; ALSPAC, 1633/3600; CATSS, 873/5306; CHOP, 624/1774; COPSAC2000, 73/263; COPSAC2010, 177/441; GENEVA/KORA F4/POPGEN (Affym), 517/1304; GENEVA/KORA F4/POPGEN (Illum), 529/1247; GENUFADext/SHIP1, 417/1667; GENUFAD/SHIP2, 259/1792; MAAS, 257/355; NFBC66, 1314/3146; PIAMA, 808/895; Raine Study, 404/972; SALTY, 103/2254; TWINS UK, 831/2044; FINNGEN, 2663/88760; UKBB, 6650/260828.

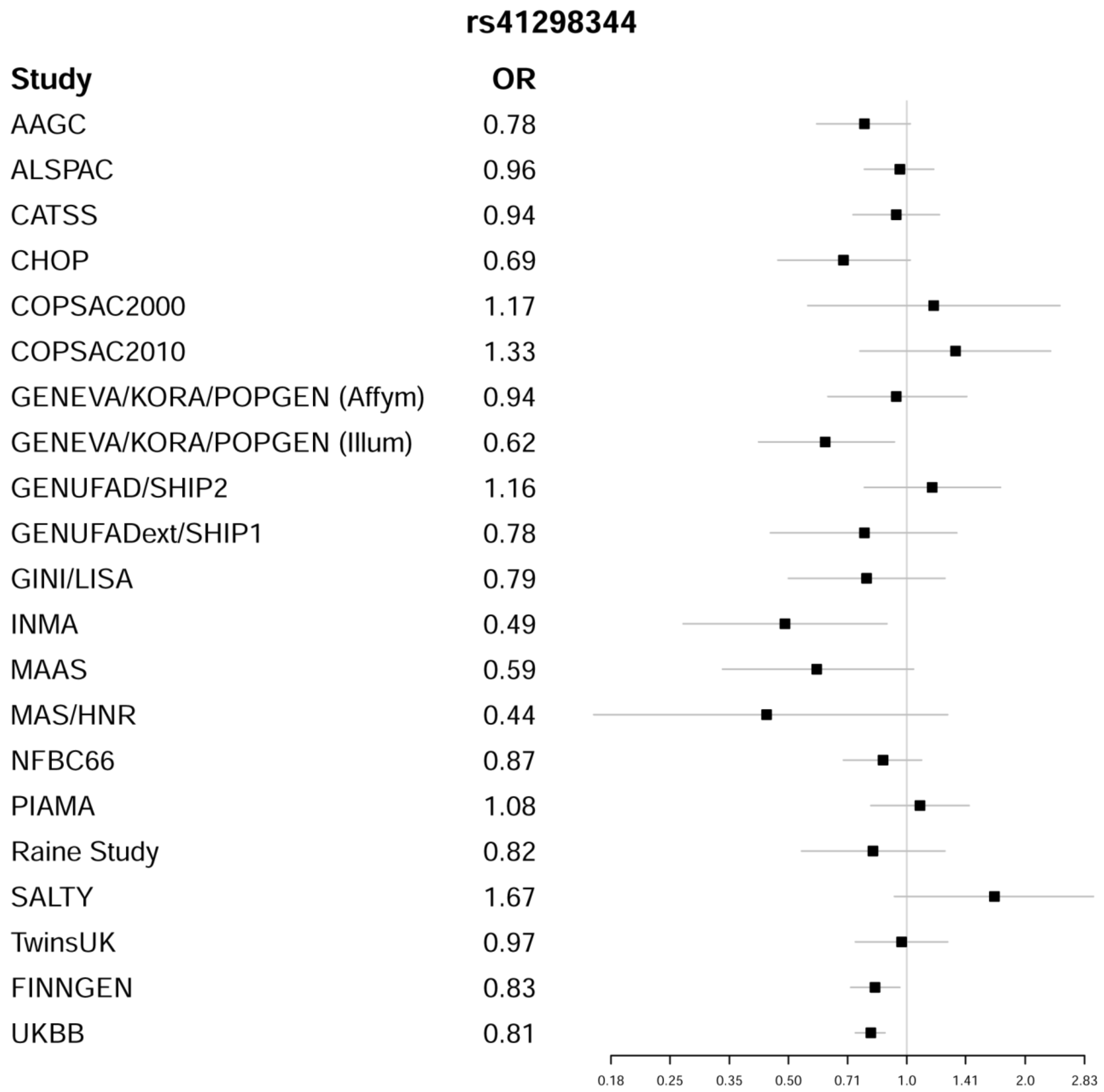

Supplementary Figure 23. Risk estimates of rs41298344 near *TNFRSF6B* in the study populations of RV set, FINNGEN, and UKBB.

Odds ratios (OR, squares) and 95% confidence intervals (error bars) are shown. Numbers of cases/controls for each study; AAGC, 934/2101 ; ALSPAC, 1633/3600; CATSS, 873/5306; CHOP, 624/1774; COPSAC2000, 73/263; COPSAC2010, 177/441; GENEVA/KORA F4/POPGEN (Affym), 517/1304; GENEVA/KORA F4/POPGEN (Illum), 529/1247; GENUFADext/SHIP1, 417/1667; GENUFAD/SHIP2, 259/1792; GINI/LISA, 442/865; INMA, 404/440; MAAS, 257/355; MAS/HNR, 104/379; NFBC66, 1314/3146; PIAMA, 808/895; Raine Study, 404/972; SALTY, 103/2254; TWINS UK, 831/2044; FINNGEN, 2663/88760; UKBB, 6650/260828.

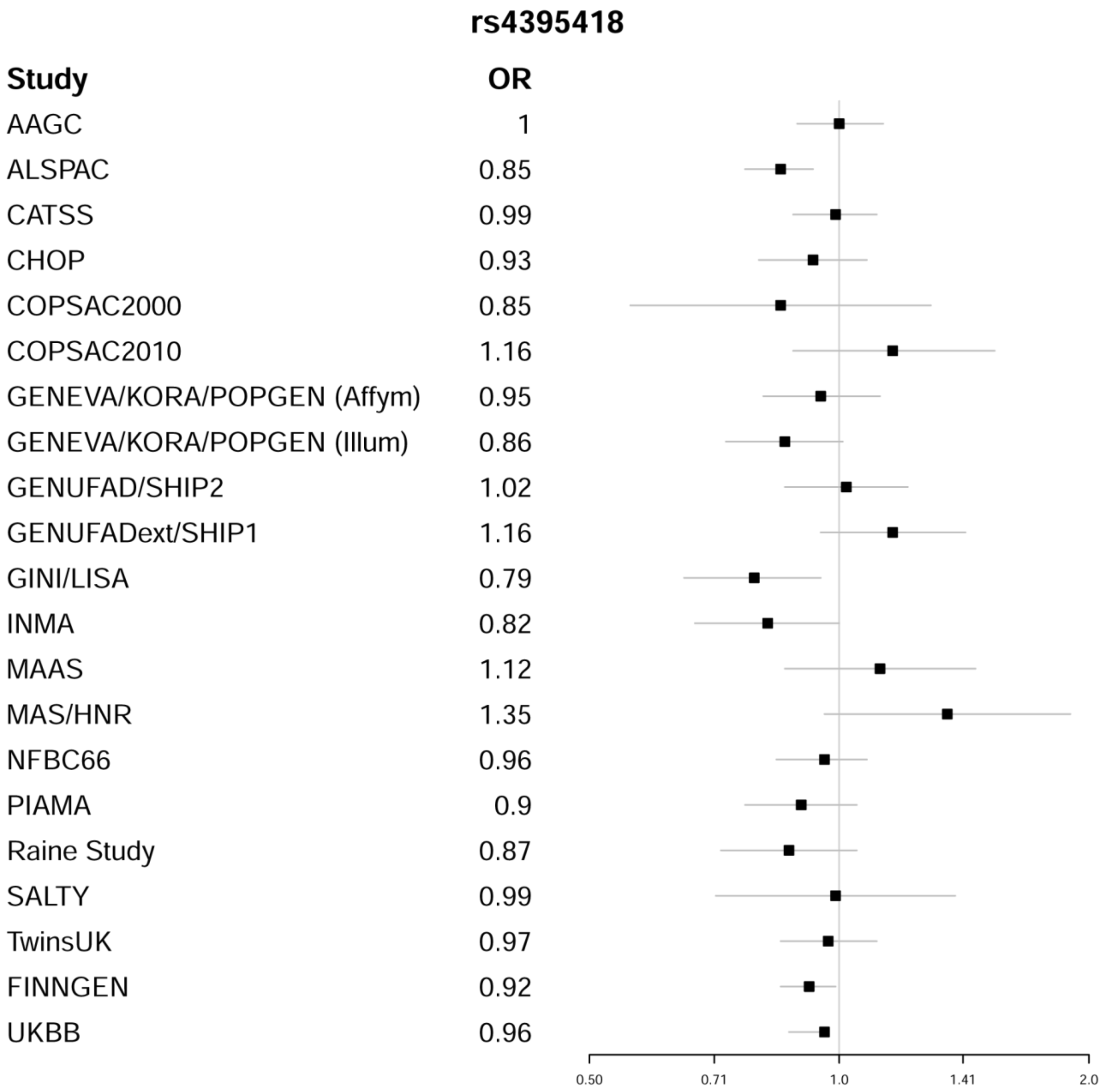

Supplementary Figure 24. Risk estimates of rs4395418 between *SATB1-AS1* and *KCNH8* in the study populations of RV set, FINNGEN, and UKBB.
Odds ratios (OR, squares) and 95% confidence intervals (error bars) are shown. Numbers of cases/controls for each study; AAGC, 934/2101 ; ALSPAC, 1633/3600; CATSS, 873/5306; CHOP, 624/1774; COPSAC2000, 73/263; COPSAC2010, 177/441; GENEVA/KORA F4/POPGEN (Affym), 517/1304; GENEVA/KORA F4/POPGEN (Illum), 529/1247; GENUFADext/SHIP1, 417/1667; GENUFAD/SHIP2, 259/1792; GINI/LISA, 442/865; INMA, 404/440; MAAS, 257/355; MAS/HNR, 104/379; NFBC66, 1314/3146; PIAMA, 808/895; Raine Study, 404/972; SALTY, 103/2254; TWINS UK, 831/2044; FINNGEN, 2663/88760; UKBB, 6650/260828.

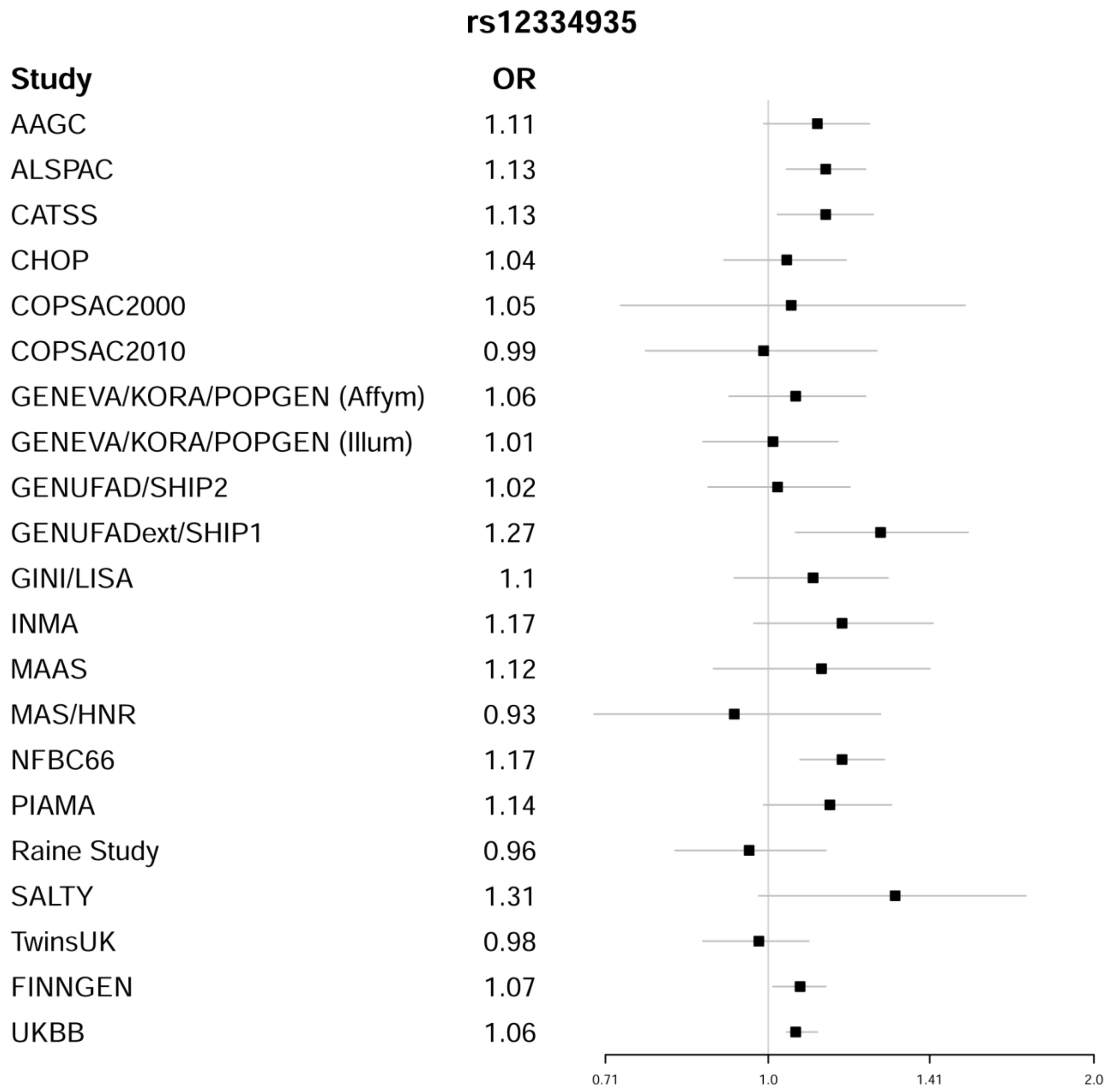

Supplementary Figure 25. Risk estimates of rs12334935 between *TRIB1* and *LINC00861* in the study populations of RV set, FINNGEN, and UKBB.

Odds ratios (OR, squares) and 95% confidence intervals (error bars) are shown. Numbers of cases/controls for each study; AAGC, 934/2101 ; ALSPAC, 1633/3600; CATSS, 873/5306; CHOP, 624/1774; COPSAC2000, 73/263; COPSAC2010, 177/441; GENEVA/KORA F4/POPGEN (Affym), 517/1304; GENEVA/KORA F4/POPGEN (Illum), 529/1247; GENUFADext/SHIP1, 417/1667; GENUFAD/SHIP2, 259/1792; GINI/LISA, 442/865; INMA, 404/440; MAAS, 257/355; MAS/HNR, 104/379; NFBC66, 1314/3146; PIAMA, 808/895; Raine Study, 404/972; SALTY, 103/2254; TWINS UK, 831/2044; FINNGEN, 2663/88760; UKBB, 6650/260828.

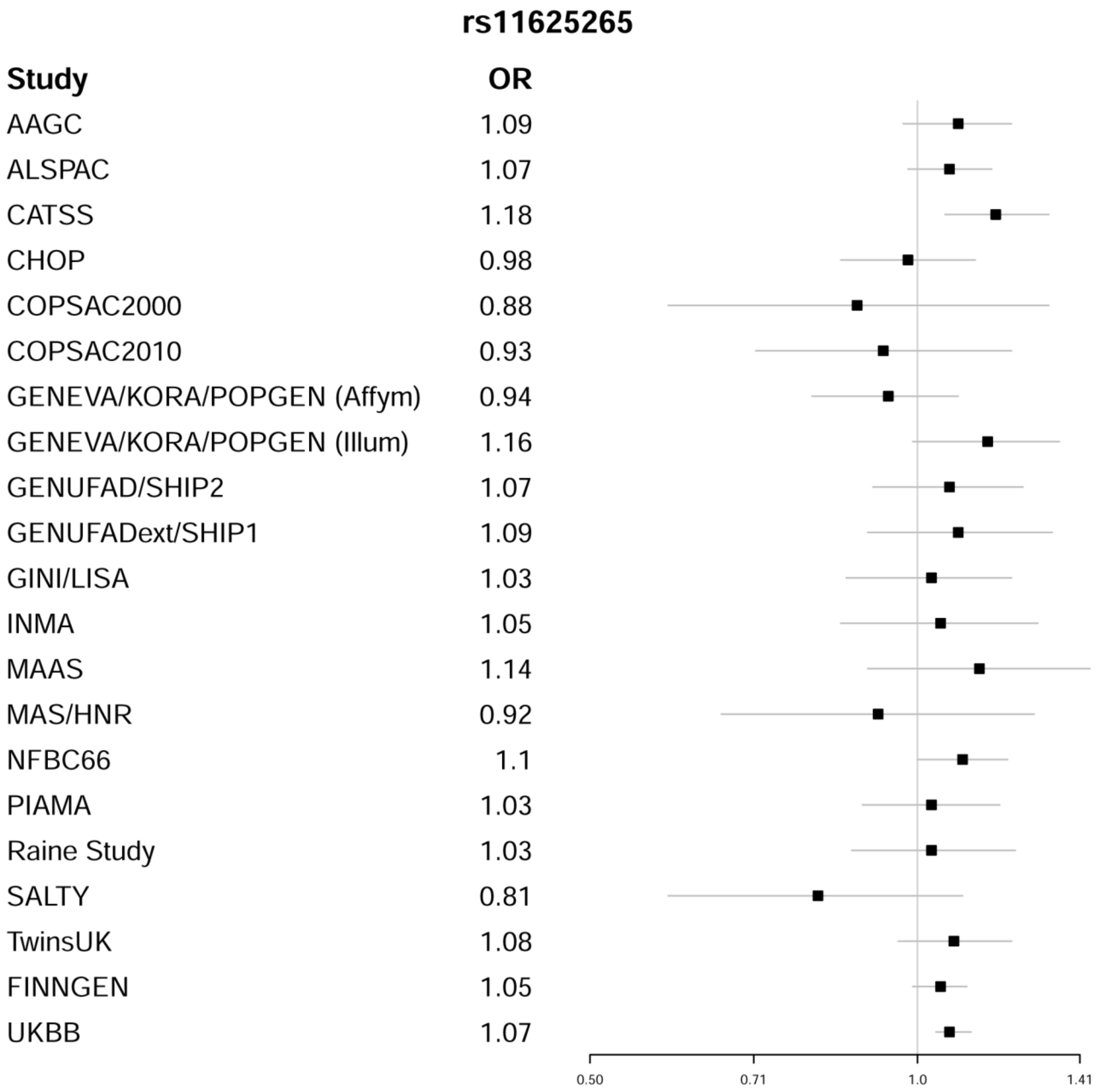

Supplementary Figure 26. Risk estimates of rs11625265 in *ZBTB1* in the study populations of RV set, FINNGEN, and UKBB.

Odds ratios (OR, squares) and 95% confidence intervals (error bars) are shown. Numbers of cases/controls for each study; AAGC, 934/2101 ; ALSPAC, 1633/3600; CATSS, 873/5306; CHOP, 624/1774; COPSAC2000, 73/263; COPSAC2010, 177/441; GENEVA/KORA F4/POPGEN (Affym), 517/1304; GENEVA/KORA F4/POPGEN (Illum), 529/1247; GENUFADext/SHIP1, 417/1667; GENUFAD/SHIP2, 259/1792; GINI/LISA, 442/865; INMA, 404/440; MAAS, 257/355; MAS/HNR, 104/379; NFBC66, 1314/3146; PIAMA, 808/895; Raine Study, 404/972; SALTY, 103/2254; TWINS UK, 831/2044; FINNGEN, 2663/88760; UKBB, 6650/260828.

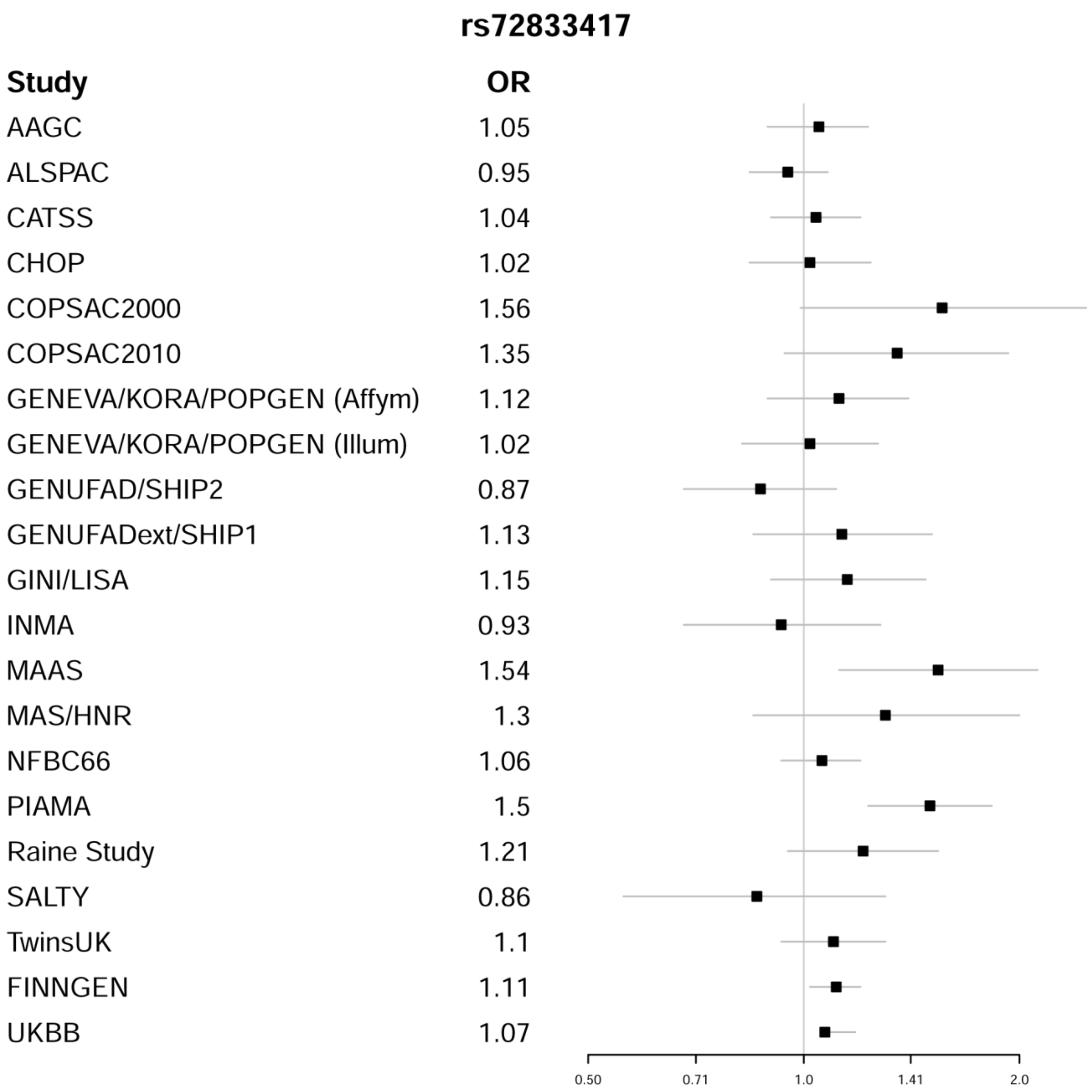

Supplementary Figure 27. Risk estimates of rs72833417 between *TBX21* and *OSBPL7* in the study populations of RV set, FINNGEN, and UKBB.

Odds ratios (OR, squares) and 95% confidence intervals (error bars) are shown. Numbers of cases/controls for each study; AAGC, 934/2101 ; ALSPAC, 1633/3600; CATSS, 873/5306; CHOP, 624/1774; COPSAC2000, 73/263; COPSAC2010, 177/441; GENEVA/KORA F4/POPGEN (Affym), 517/1304; GENEVA/KORA F4/POPGEN (Illum), 529/1247; GENUFADext/SHIP1, 417/1667; GENUFAD/SHIP2, 259/1792; GINI/LISA, 442/865; INMA, 404/440; MAAS, 257/355; MAS/HNR, 104/379; NFBC66, 1314/3146; PIAMA, 808/895; Raine Study, 404/972; SALTY, 103/2254; TWINS UK, 831/2044; FINNGEN, 2663/88760; UKBB, 6650/260828.

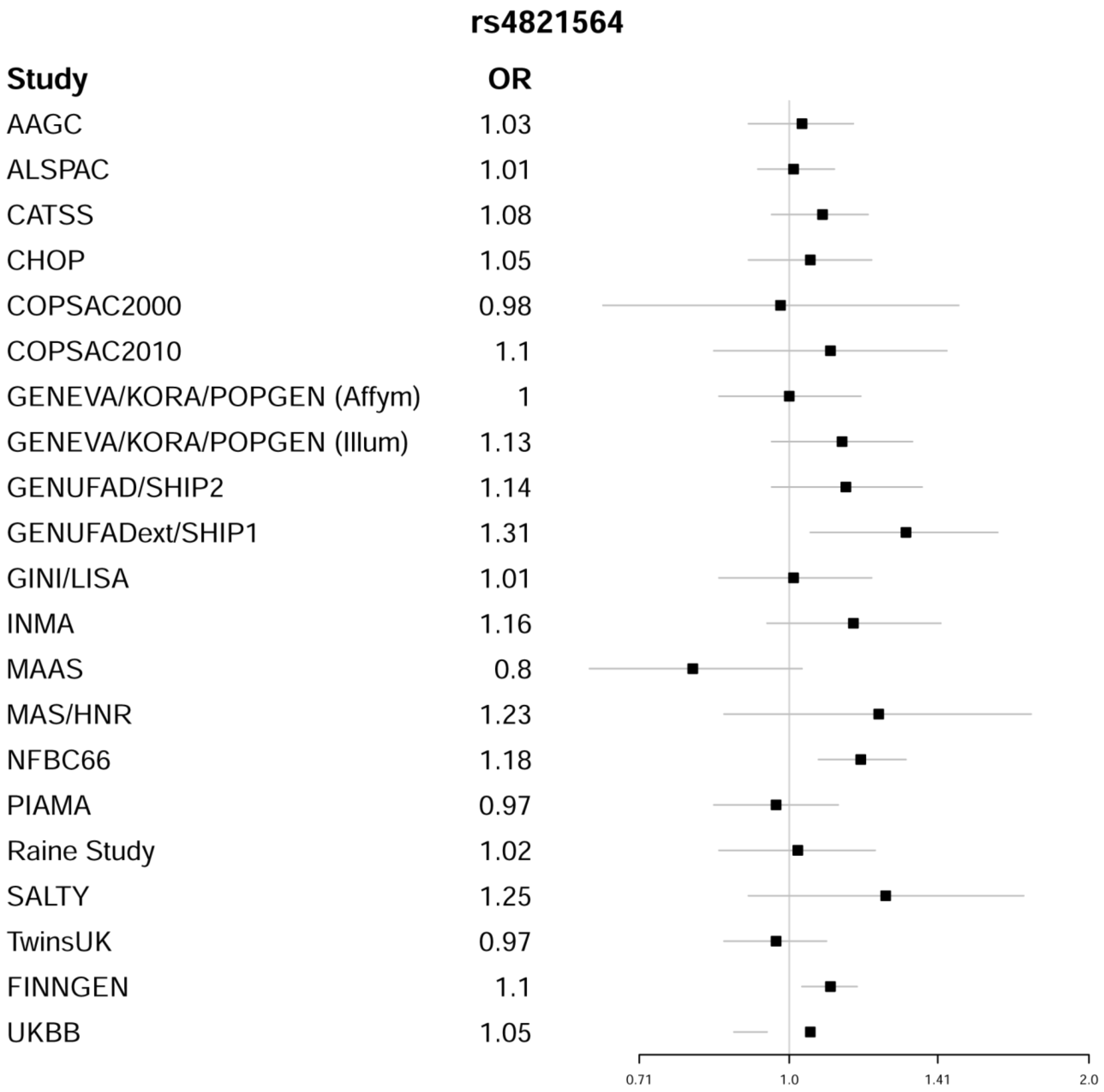

Supplementary Figure 28. Risk estimates of rs4821564 in *CSF2RB* in the study populations of RV set, FINNGEN, and UKBB.

Odds ratios (OR, squares) and 95% confidence intervals (error bars) are shown. Numbers of cases/controls for each study; AAGC, 934/2101 ; ALSPAC, 1633/3600; CATSS, 873/5306; CHOP, 624/1774; COPSAC2000, 73/263; COPSAC2010, 177/441; GENEVA/KORA F4/POPGEN (Affym), 517/1304; GENEVA/KORA F4/POPGEN (Illum), 529/1247; GENUFADext/SHIP1, 417/1667; GENUFAD/SHIP2, 259/1792; GINI/LISA, 442/865; INMA, 404/440; MAAS, 257/355; MAS/HNR, 104/379; NFBC66, 1314/3146; PIAMA, 808/895; Raine Study, 404/972; SALTY, 103/2254; TWINS UK, 831/2044; FINNGEN, 2663/88760; UKBB, 6650/260828.

a

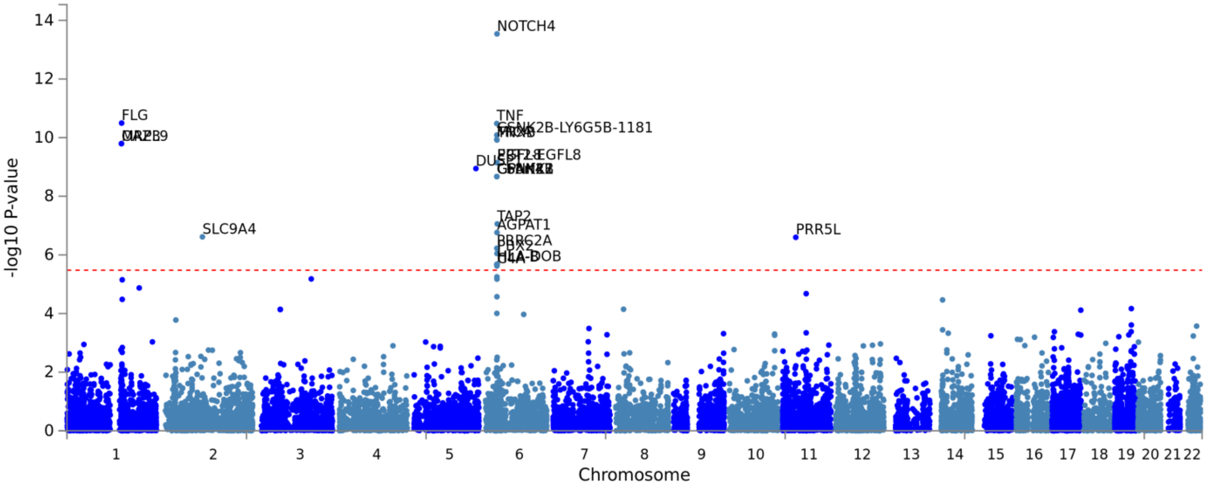

b

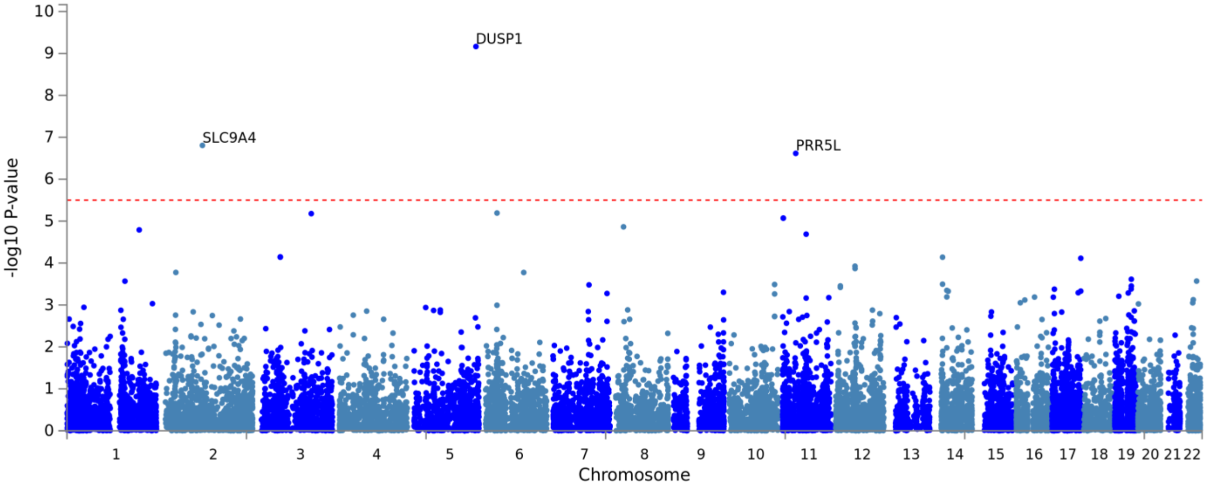

Supplementary Figure 29. Gene-level association results (a) before and (b) after adjusting for the eczema-associated variants in *FLG* and *NOTCH4*.

For each gene (dots), the genomic location (*x*-axis) and the association *P* value (*y*-axis) are indicated. The red line corresponds to the genome-wide significance threshold of 3×10^{-6} for 13,000 genes under study. Gene-level association analysis was conducted with MAGMA[2] via the FUMA webpage (https://fuma.ctglab.nl/).[3]

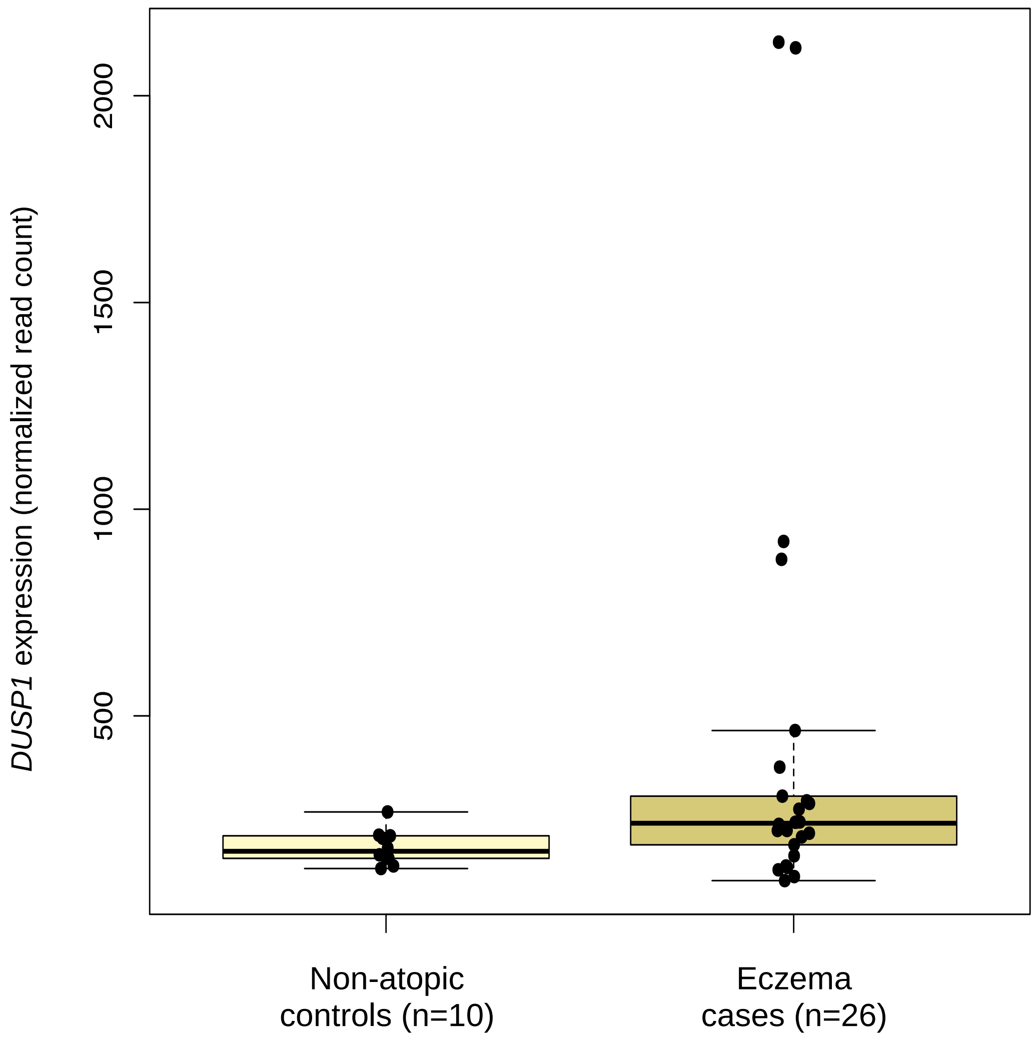

Supplementary Figure 30. *DUSP1* expression in eczema cases and non-atopic controls. Direct (single molecule) RNA sequencing of paediatric atopic skin biopsies[4] shows a significant up-regulation of DUSP1 at mRNA level compared to non-atopic site-matched control skin: fold-change 2.4, 2-sided FDR P = 0.0097 (n=26 cases and 10 controls). Box and whisker plots show minima, maxima, interquartile range and median. Data points including outliers are indicated.

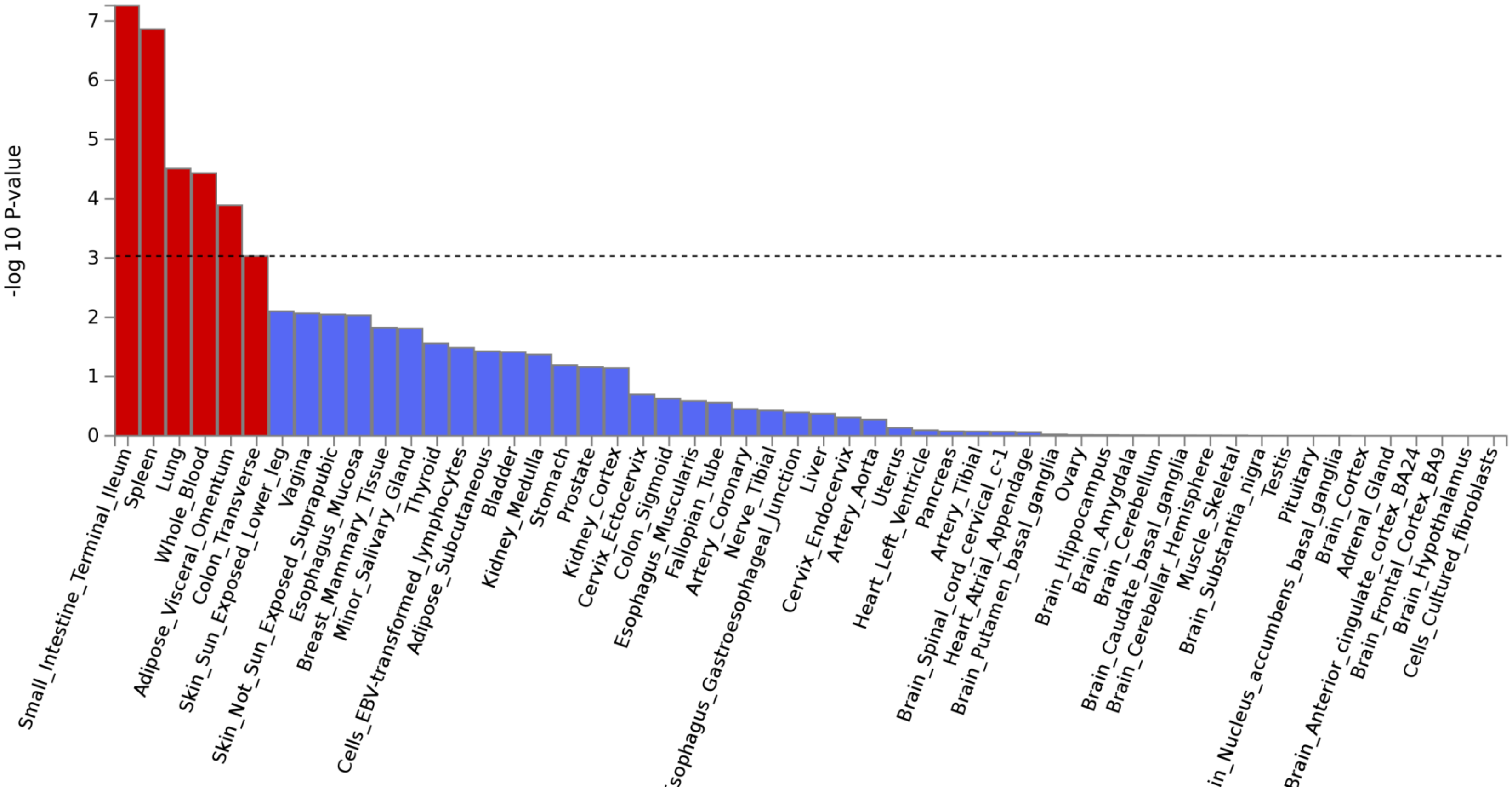

Supplementary Figure 31. Tissue expression analysis using all variants included in the GWAS.

To test for a positive correlation between tissue-specific gene expression profiles and the distribution of *P* values from the GWAS on eczema, a tissue expression analysis was performed with MAGMA.[2] Expression profiles were from 54 tissue types from GTEx.[5] Gene-based *P* values were converted to gene Z-scores. A one-sided test was performed to test the positive relationship between tissue specificity and genetic association of genes. The Bonferroni corrected significance threshold (dotted line) was set at $P < 9.25\times10^{-4}$ (=0.05/54) according to the 54 tissues tested. Significantly associated tissues are labelled in red; the corresponding *P* values are 5.5×10^{-8} (Small_Intestine Terminal Ileum), 1.4×10^{-7} (Spleen), 3.1×10^{-5} (Lung), 3.7×10^{-5} (Whole Blood), 1.3×10^{-4} (Adipose Visceral Omentum), 9.2×10^{-4} (Colon Transverse).

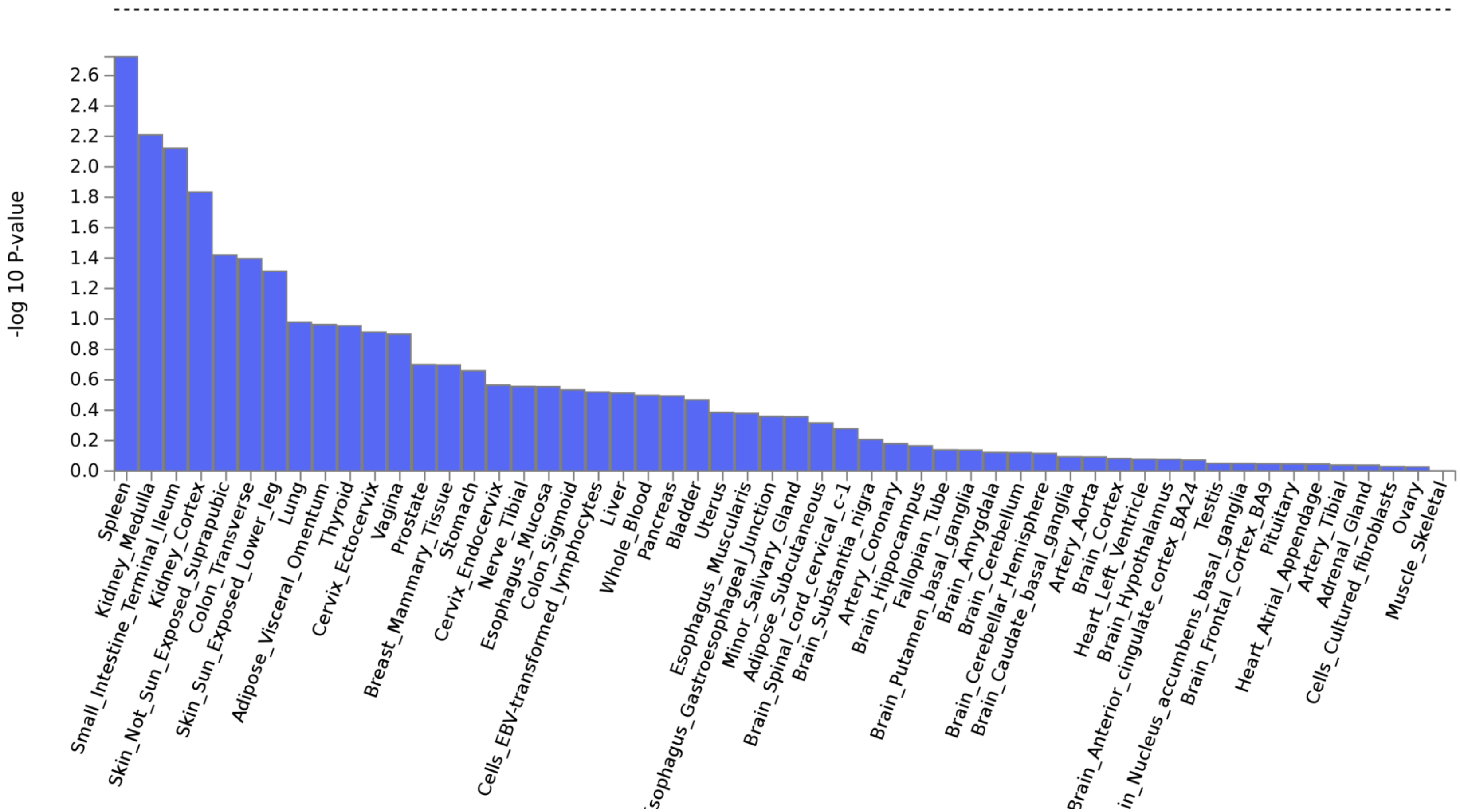

Supplementary Figure 32. Tissue expression analysis for rare and low-frequency variants with a minor allele frequency < 5%.
To test for a positive correlation between tissue-specific gene expression profiles and the distribution of *P* values from the GWAS on eczema, a tissue expression analysis was performed with MAGMA.[2] Expression profiles were from 54 tissue types from GTEx.[5] Gene-based *P* values were converted to gene Z-scores. A one-sided test was performed to test the positive relationship between tissue specificity and genetic association of genes. The Bonferroni corrected significance threshold (dotted line) was set at $P < 9.25\times10^{-4}$ (=0.05/54) according to the 54 tissues tested. Results for rare and low-frequency variants with a minor allele frequency < 5% are shown. Significantly associated tissues are labelled in red.

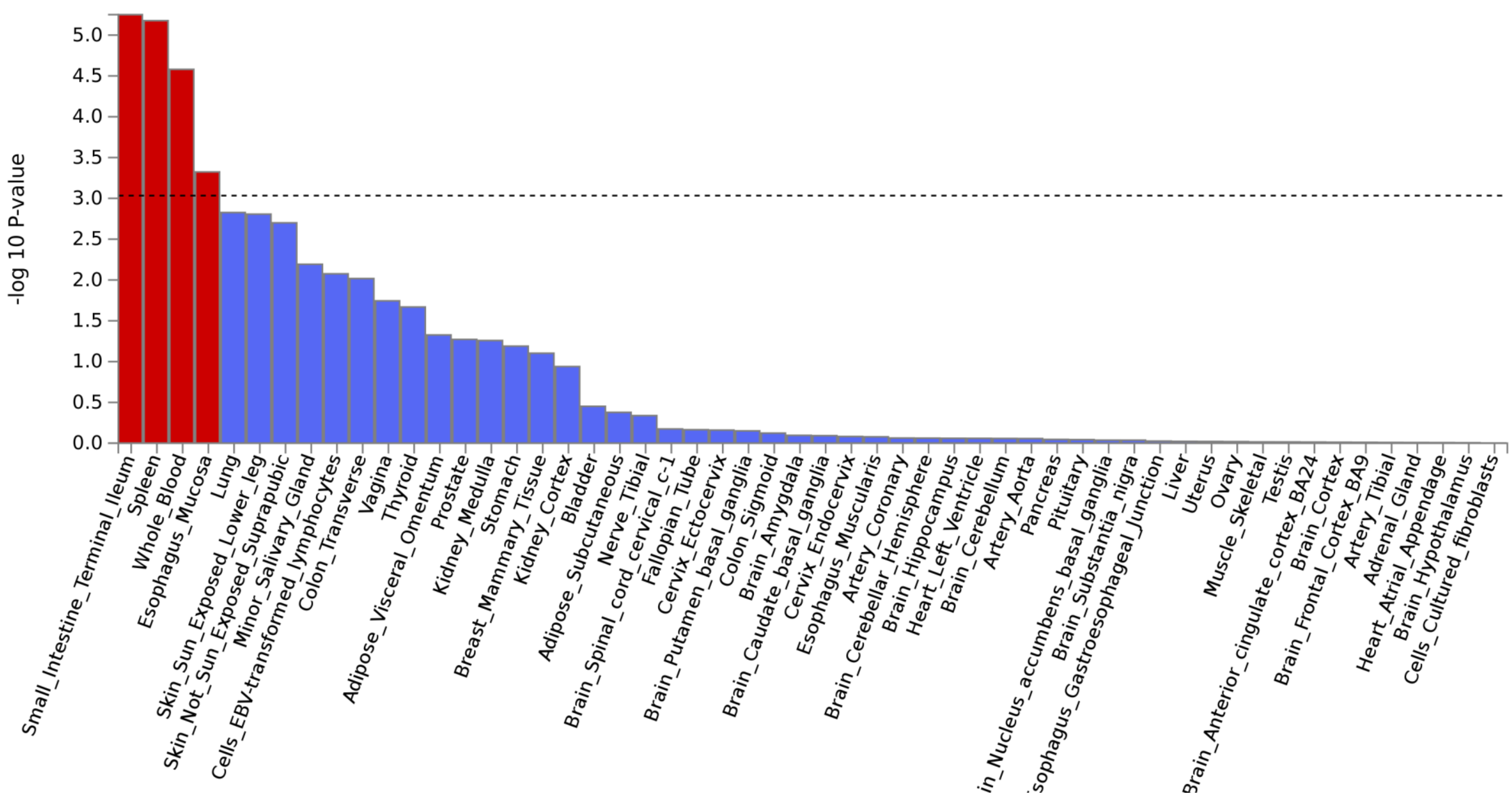

Supplementary Figure 33. Tissue expression analysis for common variants with a minor allele frequency ≥ 5%.
To test for a positive correlation between tissue-specific gene expression profiles and the distribution of *P* values from the GWAS on eczema, a tissue expression analysis was performed with MAGMA.[2] Expression profiles were from 54 tissue types from GTEx.[5] Gene-based *P* values were converted to gene Z-scores. A one-sided test was performed to test the positive relationship between tissue specificity and genetic association of genes. The Bonferroni corrected significance threshold (dotted line) was set at $P < 9.25\times10^{-4}$ (=0.05/54) according to the 54 tissues tested. Results for all common SNPs with a minor allele frequency ≥ 5% are shown. Significantly associated tissues are labelled in red; the corresponding *P* values are $5.6\times10^{\mathbf{-6}}$ (Small Intestine Terminal Ileum), $6.7\times10^{\mathbf{-6}}$ (Spleen), $2.6\times10^{\mathbf{-5}}$ (Whole Blood), $4.7\times10^{\mathbf{-4}}$ (Esophagus Mucosa).

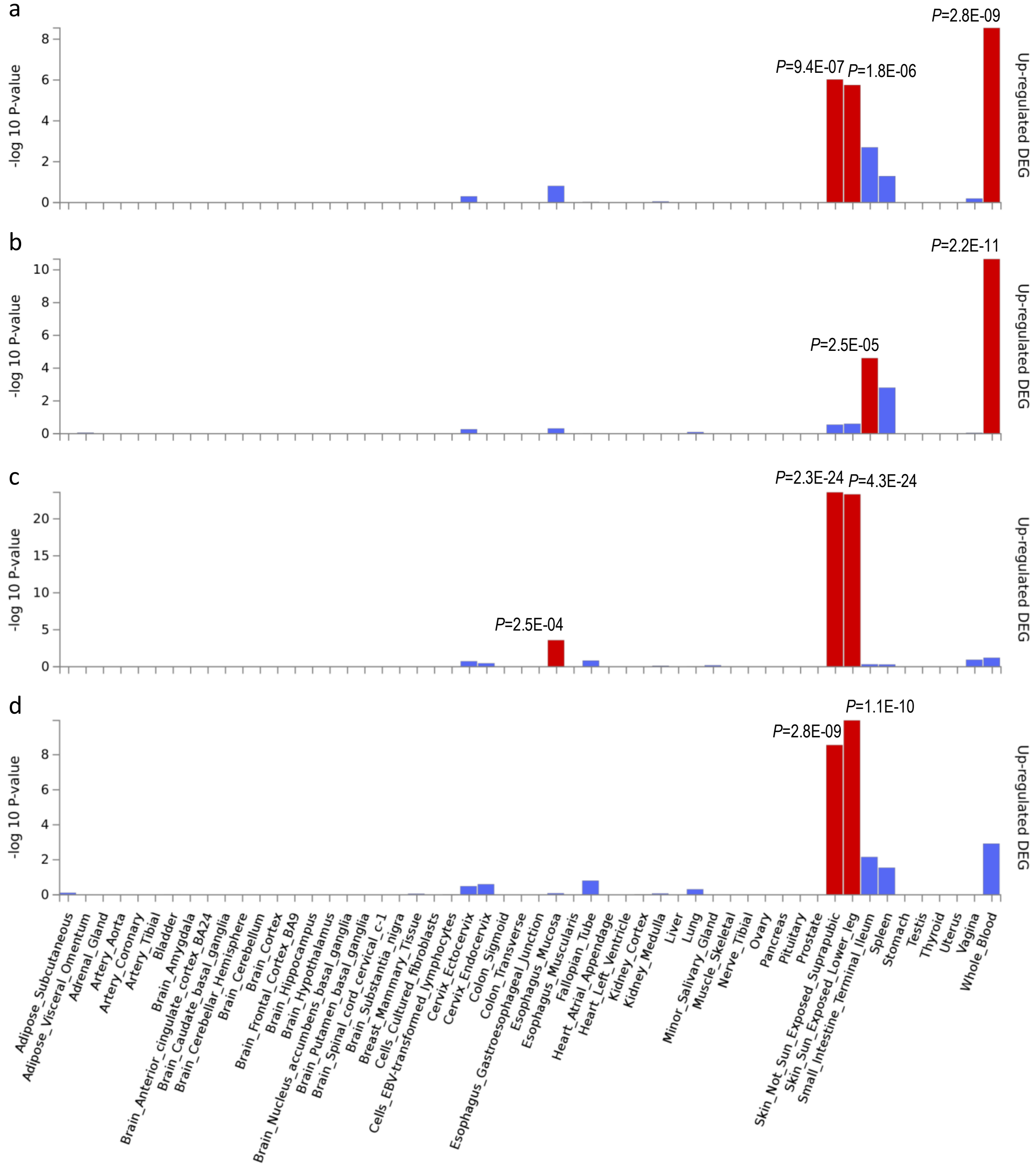

Supplementary Figure 34. Enrichment of differentially expressed genes in 54 tissue types.

To obtain sets of differentially expressed genes (DEGs) for each of 54 tissue types, the normalized expression was used. Two-sided Student's t-tests were performed per gene per tissue against all other tissues. Genes with Bonferroni corrected *P* value < 9.25×10^{-4} (=0.05/54) and absolute log fold change ≥ 0.58 were defined as a DEG set in a given tissue. Genes were prioritized based on (a) all variants, (b) common variants (MAF ≥ 5%), (c) rare/low-frequency variants (MAF < 5%), and (d) variants with MAF < 5% after conditioning on the 3 identified *FLG* variants. *P* values for significant DEGs are indicated. All of them were up-regulated in the respective tissues (labelled in red).

Supplementary Note 1

Australian Asthma Genetic Consortium (AAGC)

Recruitment and phenotype definition

As part of the AAGC, a GWAS on eczema in 7,197 unrelated individuals of European ancestry ascertained from the Australian population as described in detail elsewhere was performed.[6] For this analysis, 3,035 individuals (56% females, mean age 36 years, range 3 to 89), including 934 who reported having had atopic dermatitis at any point in their lifetime and 2,101 atopic dermatitis-free controls were included in the study. These individuals participated in one of the five studies: QIMR (N=1,849), CAPS (N=53), LIWA (N=637),MESCA (N=127(or TAHS (N=369). Participants provided informed consent to participate in this study, which was approved by the respective ethics committees.

Genotyping and imputation

Genotyping was performed with Illumina 610K and stringent quality control filters were applied as described previously.[6] Haplotype phasing and imputation in the HRC panel version1.1 was performed using the University of Michigan server.[7] Variants with an imputation quality score of $r^2 < 0.5$ or out of Hardy-Weinberg-Equilibrium $P < 10^{-12}$ were excluded.

Statistical analysis

A score test implemented in RVTESTS[8] was used to test the association between SNP allelic dosage and eczema status. Sex and age were included as covariates.

Acknowledgments and funding

The AAGC was funded by a project grant from the NHMRC (613627).

AAGC members:

Graham Jones[1], Patrick Danoy[2], Svetlana Baltic[3], Desiree Mészáros[4], Catherine Hayden[5], Sarah E Medland[6], Andrew J. Kemp[7], Faang Cheah[3], Dale R. Nyholt[6], Melissa C Southey[8], Mary Roberts[9], Scott D. Gordon[6], Euan R. Tovey[1], Loren Price[3], Margaret J. Wright[6], James Markos[10], Anjali K. Henders[6], Graham Giles[11], Li P. Chung[3], Paul S. Thomas[12], Ian Feather[13], Pamela A Madden[14], Suzanna Temple[3], Stephen Morrison[15], Chalermchai Mitrpant[3], Brad Shelton[3], Andrew C. Heath[14], Mark Jenkins[2], Warwick J. Britton[16], John L. Hopper[17], Stephen R. Leeder[18], Haydn Walters[4], Michael J. Abramson[19], Matthew A Brown[2]

[1]Woolcock Institute of Medical Research, University of Sydney, Sydney, Australia.

[2]University of Queensland Diamantina Institute, Princess Alexandra Hospital, Brisbane,Australia.

[3]Lung Institute of WA and Centre for Asthma, Allergy and Respiratory Research, University of WA, Perth, Australia.

[4]Menzies Research Institute, Hobart, Australia.

[5]School of Pediatrics and Child Health, Princess Margaret Hospital for Children, Perth, Australia.

[6]The Queensland Institute of Medical Research, Brisbane, Australia.

[7]The Children's Hospital, Westmead, Sydney, Australia.

[8]Department of Pathology, The University of Melbourne, Melbourne, Australia.

[9]Departmenet of Respiratory Medicine, Royal Children's Hospital, Parkville, Australia.

[10]Launceston General Hospital, Lauceston, Australia.

[11]Cancer Epidemiology Centre, The Cancer Council Victoria, Melbourne, Australia.

[12]Faculty of Medicine, University of New South Wales, Sydney, Australia.
[13]Gold Coast Hospital, Southport, Australia.
[14]Washington University School of Medicine, St Louis, United States.
[15]University of Queensland, Brisbane, Australia.
[16]Centenary Institute of Cancer Medicine & Cell Biology, Royal Prince Alfred Hospital, Camperdown, Australia.
[17]Centre for Molecular, Environmental, Genetic and Analytic Epidemiology, University of Melbourne, Melbourne, Australia.
[18]Australian Health Policy Institute, University of Sydney, Sydney, Australia.

Avon Longitudinal Study of Parents and Children (ALSPAC)

Recruitment and phenotype definition

ALSPAC recruited 15,247 pregnant women resident in Avon, UK with expected dates of delivery 1st April 1991 to 31st December 1992, resulting in 14,775 live births and 14,701 children wo where alive at 1 year of age. Enrolment is described in more detail in the cohort profile papers[9,10] and via the study website http://www.bris.ac.uk/alspac/researchers/data-access/data-dictionary/. Please note that the study website contains details of all the data that is available through a fully searchable data dictionary and variable search tools. Biological samples including DNA have been collected for 10,121 children from this cohort. Ethical approval for the study was obtained from the ALSPAC Ethics and Law Committee and the Local Research Ethics Committees (NHS Haydock REC: 10/H1010/70). Written and informed consent for the use of data collected via questionnaires and clinics was obtained from the parents following the recommendations of the ALSPAC Ethics and Law Committee at the time.
The children have been followed up with regular questionnaires and clinic visits. For the current study data collected from the questionnaires was used to classify children as eczema cases or controls. When the children were approximately 81, 91, 103 months , 10, 13 and 14 years, parents were asked the following questions [possible answers]:
1. Has your child in the past 12 months had eczema? [yes, saw a Dr; Yes, but did not see a Dr; No, did not have]
2. Has a doctor ever actually said that your child has eczema? (10 & 14 years only) [yes; no]
We defined cases as the children of parents who answered 'Yes, and saw a Dr' to Q1 or 'yes' to Q2. We defined controls as the children who were not cases and whose parent answered 'no' to Q2 at age 14 years.

Genotyping and imputation

GWAS data was generated by Sample Logistics and Genotyping Facilities at the Wellcome Trust Sanger Institute and LabCorp (Laboratory Corporation of America) using support from 23andMe. 975 individuals were genotyped at the WTSI and 9382 were genotyped at LabCorp, both on the Illumina 550K Custom chip. All individuals of non-European ancestry, ambiguous sex, extreme heterozygosity (< 0.32 or >0.345 in the WTSI set and < 0.31 or >0.33 in the LabCorp set), cryptic relatedness (>10% IBD) and high missingness (>3%) were removed. SNPs with low genotyping rate (< 95%), with low minor allele frequency (< 1%), out of Hardy Weinberg equilibrium ($p< 5x10^{-7}$) or from the pseudo-autosomal region of the X chromosome

were excluded. 8365 individuals typed on 464,311 probes remained. Haplotype phasing and imputation in the HRC panel version1.1 was performed using the University of Michigan server.[7] Variants with an imputation quality score of $r^2 < 0.5$ or out of Hardy-Weinberg-Equilibrium $P < 10^{-12}$ were excluded.

Statistical analysis

A score test implemented in RVTESTS[8] was used to test the association between SNP allelic dosage and eczema status. Sex, age and the first 10 principle components were included as covariates.

Acknowledgements and funding

We are extremely grateful to all the families who took part in this study, the midwives for their help in recruiting them, and the whole ALSPAC team, which includes interviewers, computer and laboratory technicians, clerical workers, research scientists, volunteers, managers, receptionists and nurses. The UK Medical Research Council and the Wellcome Trust (Grant ref: 217065/Z/19/Z) and the University of Bristol provide core support for ALSPAC. GWAS data was generated by Sample Logistics and Genotyping Facilities at the Wellcome Trust Sanger Institute and LabCorp (Laboratory Corporation of America) using support from 23andMe.

Child and Adolescent Twin Study in Sweden (CATSS)

Recruitment and phenotype definition

The Child and Adolescent Twin Study in Sweden (CATSS) is the first of two cohorts from the Swedish Twin Registry (STR) that contributed to the meta-analysis. The other one was Screening Across the Lifespan Twin Study: the Younger (SALTY, born 1943-1958). A detailed summary of the collection of biosamples within these two cohorts is available in previous publications[11-14] and briefly outlined below. Genotyping was dependent on biosample availability and quality of extracted DNA. As the cohorts include both identical (monozygotic, MZ) and fraternal (dizygotic, DZ) twin pairs, genotyping was carried out for only one of the twins within MZ pairs and imputed in the other. In DZ pairs with available biosamples, both were genotyped when possible. The phenotype definitions within the STR cohorts were created using a combination of questionnaire data; population-based register data sources; the Swedish National Patient Register from 1987, and the Swedish Prescribed Drug Register from July 2005. All studies were approved by the Regional Ethical Review Board in Stockholm, Sweden and all participants gave informed consent. Here, we summarise methods for CATSS.

The CATSS is an ongoing longitudinal twin study targeting all twins born from 1992 and living in Sweden. Since 2004, twins are invited to participate in CATSS following their ninth birthday. During the first three years of data collection, twelve-year-old twins were also invited. Participation in CATSS starts with a parental telephone interview on the children’s health, perinatal factors, living situation. A module including questions regarding the twin pair’s physical similarities is the basis for an algorithm-based assessment of zygosity. Since 2008, twins have also been offered a DNA-based zygosity test using the saliva samples collected by mail in connection with invitation to the study. DNA from saliva is then stored in the biobank of Karolinska Institutet. To date, approximately n=29100 twins have participated in CATSS-9/12.

Eczema was defined if the study participant had answered YES to “Has the child been diagnosed with atopic dermatitis or atopic eczema by a doctor?”.

Exclusion for the controls included an affirmative response to the above criteria; if they had any dispense of R03AC, R03AK, R03BA or R03DC; if they had answered that the child had food allergies like celiac disease or lactose intolerant; or if they answered yes to any of the following: Has or has the child had asthma, hay fever or eczema.

Genotyping and imputation

Saliva samples from the CATSS and SALTY (described below) cohorts were analysed jointly. DNA was extracted using either the Chemagic STAR instrument from Hamilton Robotics, with magnet bead purification kits from Chemagen, or the Puregene extraction kit (Gentra systems, Minneapolis, USA). Genotyping was performed in 18 batches at the SNP&SEQ Technology Platform in Uppsala, Sweden, using the Illumina PsychArray bead chip. Genotype calls from the zCall algorithm for rare variants were combined with those from the Illumina GenCall algorithm to increase sensitivity at low minor allele frequencies. After initial intensity-level quality control, 18,193 samples remained. Additional QC filtering was applied as follows: SNPs with missingness > 2%, SNPs with more than 10% discordant genotypes across replicates or MZ pairs, SNPs out of Hardy-Weinberg equilibrium (exact test P value $< 10^{-6}$), SNPs with clear batch effects or absolute MAF difference from 1000 Genomes European samples > 10%, Y-chromosome and mitochondrial SNPs, and SNPs with minor allele count < = 1 were all excluded. Further, individuals with missingness > 2%, individuals with deviant autosomal heterozygosity (autosomal inbreeding coefficient F outside [-0.02, 0.02]), individuals showing excessive mean relatedness to the rest of the sample (mean relatedness > 6 s.d. above the sample mean), individuals where genotype-based sex did not match phenotype information, and individuals identified as non-European ancestral outliers were excluded. Non-genotyped monozygous twins in the study were imputed from their genotyped twin, resulting in a total of 21,752 individuals with genotypes. The actual number of samples used for the present analyses was limited by the available phenotype data. Haplotype phasing and imputation in the HRC panel version1.1 was performed using the Sanger server at the Wellcome Sanger Institute.[7] Variants with an imputation quality score of info < 0.5 or out of Hardy-Weinberg-Equilibrium $P < 10^{-12}$ were excluded.

Statistical analysis

A score test implemented in RVTESTS[8] was used to test the association between SNP allelic dosage and eczema status. Sex and the first four principle components were included as covariates. A kinship matrix was used to adjust for relatedness among the samples.

Acknowledgment and funding

We acknowledge The Swedish Twin Registry for access to data. The Swedish Twin Registry is managed by Karolinska Institutet and receives funding through the Swedish Research Council under the grant no 2017-00641. Financial support was also provided from the Swedish Research Council (grant no 2018-02640) and the Swedish Heart-Lung Foundation (grant no 20180512). We wish to thank the Biobank at Karolinska Institutet for professional biobank service.

Children’s Hospital of Philadelphia (CHOP)

Recruitment and phenotype definition

The Center for Applied Genomics (CAG) has recruited ~80K pediatric patients from CHOP. Enrolment into the study is random and therefore encompasses all of the major common pediatric disorders including atopic dermatitis. Upon enrollment, CAG is authorized to extract the patients' medical history from their electronic medical record (EMR) and store the information in a de-identified database. Approximately 90% of enrollees consent to a yearly EMR update. Biological samples including DNA have been collected from all patients. The study was approved by the CHOP institutional Review Board (IRB). Written informed consent for participation in the study was obtained from all participants and their parents or guardians.

Cases were defined from the electronic medical record according to one of the following two criteria. 1) individuals 60 days old or older with relevant ICD9 code for Atopic Dermatitis (691.8) in two or more in person visits to the hospital, on separate calendar days. Plus, two or more prescriptions for Atopic Dermatitis-related medications; or 2) individuals 60 days old or older with relevant ICD9 code for Atopic Dermatitis (691.8) in three or more in person visits, on separate calendar days. Individuals with ICD9 codes for Scabies, Wiskott-Aldrich Syndrome, Allergic Purpura or Ichtyosis congenita were excluded from the study.

Controls were defined as individuals 60 days or older with two or more in person visits to the hospital over the preceding 5 years, no diagnosis codes for atopic dermatitis (691.8), no history of relevant medications and no exclusionary ICD 9 codes. Exclusionary codes included a comprehensive list of skin diseases and allergic conditions.

Genotyping and imputation

Samples were genotyped on either the Illumina HumanHap550 or the HH610 at CAG, following the manufacturers' instructions. Standard quality control parameters were applied to the dataset, samples with chip-wide genotyping failure rate were excluded; SNPs with minor allele frequencies of < 1%; genotyping failure rates of greater that 2% or Hardy-Weinberg *P* Values less that $1x10^{-6}$ were excluded from further analysis.

Haplotype phasing and imputation in the HRC panel version1.1 was performed using the University of Michigan server.[7] Variants with an imputation quality score of $r^2 < 0.5$ or out of Hardy-Weinberg-Equilibrium $P < 10^{-12}$ were excluded.

Statistical analysis

A score test implemented in RVTESTS[8] was used to test the association between SNP allelic dosage and eczema status. Sex, age and the first 2 principle components were included as covariates.

Acknowledgements and funding

Genotyping was funded by an Institute Development Fund and an Adele S. and Daniel S. Kubert Estate gift to the Center for Applied Genomics. Additional funding for phenotyping and analysis was provided by a U01 award from the National Institute of Health (HG006830-02).

Copenhagen Prospective Studies on Asthma in Childhood 2000 (COPSAC2000)

Recruitment and phenotype definition

The COPSAC2000 birth cohort study is a prospective clinical study of a birth cohort of 411 infants born to mothers with a history of asthma. The newborns were enrolled at the age of 1 month, the recruitment of which was previously described in detail.[15] The study was approved by the Ethics Committee for Copenhagen (KF 01- 289/96) and The Danish Data Protection Agency (2008-41-1754) and informed consent was obtained from both parents

The families used doctors employed at the clinical research unit, and not the family practitioner, for diagnosis and treatment of AD and other skin-related symptoms. Skin lesions were described at both scheduled visits at 6-monthly intervals and acute visits with skin symptoms according to pre-defined morphology and localization; AD was defined based on the Hanifin-Rajka criteria.[16]

Genotyping and imputation

High throughput genome-wide SNP genotyping were performed using the Illumina infinium™ II HumanHap550 v1 and v3 platform (Illumina, San Diego), at the Children's Hospital of Philadelphia's Center for Applied Genomics. Haplotype phasing and imputation in the HRC panel version1.1 was performed using the University of Michigan server.[7] Variants with an imputation quality score of $r^2 < 0.5$ or out of Hardy-Weinberg-Equilibrium $P < 10^{-12}$ were excluded.

Statistical analysis

A score test implemented in RVTESTS[8] was used to test the association between SNP allelic dosage and eczema status. Sex, age and the first 4 principle components were included as covariates.

Acknowledgements and funding

COPSAC is funded by private and public research funds, all of which are listed at www.copsac.com. Aase and Ejnar Danielsens Fond, the Lundbeck Foundation, the Danish State Budget, the Danish Council for Strategic Research, the Danish Council for independent Research, and the Capital Region Research Foundation has provided core support for COPSAC. No pharmaceutical company was involved in the study. The funding agencies did not have any influence on design and conduct of the study; collection, management, and interpretation of the data; or preparation, review, or approval of the manuscript.

Copenhagen Prospective Studies on Asthma in Childhood 2010 (COPSAC2010)

Recruitment and phenotype definition

The COPSAC2010 birth cohort is a population based longitudinal clinical study of 800 pregnant women and their offspring. The families are monitored closely from week 24 of mothers' pregnancy till age 3 year of the offspring with 10 scheduled visits to the research center and additional visits at onset of any skin or wheezy symptoms.

The families used doctors employed at the clinical research unit, and not the family practitioner, for diagnosis and treatment of AD and other skin-related symptoms. Skin lesions were described at both scheduled visits at 6-monthly intervals and acute visits with skin symptoms according to pre-defined morphology and localization; AD was defined based on the Hanifin-Rajka criteria.[16]

Genotyping and imputation

Genotyping of 951,117 genetic markers were carried on the Illumina infinium HumanOmniExpressExome Bead chip at the AROS Applied Biotechnology AS center, in Aarhus, Denmark. Genotypes were called with Illumina's Genome Studio software. All individuals underwent quality control (QC) filters, where individuals with Hardy-Weinberg equilibria p values $>10^{-6}$, minor allele frequency (MAF>0.01), individual genotyping call rate > 0.95, and SNP genotyping call rate > 0.95 were retained. We excluded individuals with gender mismatches, genetic duplicates, outlying heterozygosity > 0.27 and < 0.037, and those individuals not clustering with the CEU individuals (Utah residents with ancestry from northern and Western Europe) through a multi-dimensional clustering analyses (MDS) seeded with individuals from the international Hap Map Phase 3. Haplotype phasing and imputation in the HRC panel version1.1 was performed using the University of Michigan server.[7] Variants with an imputation quality score of $r^2 < 0.5$ or out of Hardy-Weinberg-Equilibrium $P < 10^{-12}$ were excluded.

Statistical analysis

A score test implemented in RVTESTS[8] was used to test the association between SNP allelic dosage and eczema status. Sex, age and the first 4 principle components were included as covariates.

Acknowledgements and funding

COPSAC is funded by private and public research funds, all of which are listed at www.copsac.com. Aase and Ejnar Danielsens Fond, the Lundbeck Foundation, the Danish State Budget, the Danish Council for Strategic Research, the Danish Council for independent Research, and the Capital Region Research Foundation has provided core support for COPSAC. No pharmaceutical company was involved in the study. The funding agencies did not have any influence on design and conduct of the study; collection, management, and interpretation of the data; or preparation, review, or approval of the manuscript.

GENEVA / KORA F4 / POPGEN

Recruitment and phenotype definition

AD patients were recruited from tertiary dermatology clinics based at three centers (Technische Universität Munich, as part of the GENEVA study, University of Kiel, University of Bonn). German controls were obtained from the population-representative PopGen biorepository[17] and the population-based KORA study.[18,19]

Atopic dermatitis was diagnosed on the basis of a skin examination by experienced dermatologists according to standard criteria, which included the presence of chronic or chronically relapsing pruritic dermatitis with the typical morphology and distribution.[20]

Genotyping and imputation

Prior imputation we excluded samples with extensive missing data rate (>5%), excess of heterozygosity or homozygosity and ambiguous sex. We examined IBS and excluded close related samples with PI_HAT>0.1875 (halfway between expected IBD for third- and second degree relatives) as well as outliers of unusual ancestry by MDS analysis. SNPs with low genotyping rate (< 95%), low minor allele frequency (< 1%), strong deviation from Hardy-Weinberg equilibrium ($p < 10^{-8}$) and differential call rate between cases and controls were excluded.

Cases and controls with high quality SNPs were matched and imputed by array type. Haplotype phasing and imputation in the HRC panel version1.1 was performed using the University of Michigan server.[7] Variants with an imputation quality score of $r^2 < 0.5$ or out of Hardy-Weinberg-Equilibrium $P < 10^{-12}$ were excluded.

Statistical analysis

A score test implemented in RVTESTS[8] was used to test the association between SNP allelic dosage and eczema status. Sex, age and the first 4 principle components were included as covariates.

Acknowledgements and funding

The project received infrastructure support through the DFG Clusters of Excellence “inflammation at interfaces” (grants EXC306 and EXC306/2), and was supported by the German Federal Ministry of Education and Research (BMBF) within the framework of the e:Med research and funding concept (sysINFLAME, grant # 01ZX1306A), and the PopGen 2.0 network (01EY1103). The KORA study was initiated and financed by the Helmholtz Zentrum München – German Research Center for Environmental Health, which is funded by the German Federal Ministry of Education and Research (BMBF) and by the State of Bavaria. Furthermore, KORA research was supported within the Munich Center of Health Sciences (MC-Health), Ludwig-Maximilians-Universität, as part of LMUinnovativ.

GEnetic analysis of NUclear Families with Atopic Dermatitis, extended / Study of Health in Pomerania - 1 (GENUFAD ext / SHIP1)

Recruitment and phenotype definition

Atopic dermatitis patients were recruited at Charité Universitätsmedizin Berlin, Germany for the extended GENUFAD study (GEnetic analysis of NUclear Families with Atopic Dermatitis) and have been described previously.[21,22] Children of the extended GENUFAD study were unrelated individuals recruited based on moderate to severe atopic dermatitis and an age of onset below two years. A total of 417 unrelated atopic dermatitis patients were included in the present study. All controls originated from the population-based Study of Health in Pomerania (SHIP),[23] which included individuals in the North-Eastern part of Germany. The SHIP set was split for two case-control studies by a random function. 1,667 unrelated individuals were included in SHIP-1. All cases had early onset eczema (< 2years) diagnosed by a physician according to standard criteria.[16] Controls were unrelated individuals from the population-based SHIP cohort.

Genotyping and imputation

All cases and controls were genotyped with Affymetrix Genome-Wide Human SNP Array 6.0. Individuals with a call rate < 0.95 were excluded from the study. In addition, samples were excluded when the gender estimated from X-chromosome heterozygosity did not match the clinical records. SNPs were filtered according to the following criteria: i) low call rate (< 0.95 in cases or controls), ii) low allele frequency (MAF < 0.01 in cases or controls), iii) genotypes out of Hardy-Weinberg equilibrium ($P < 0.00001$ in cases or $P < 0.0005$ in controls). Additionally, SNPs with a call rate < 0.99 were excluded if having MAF < 0.05 or if they were out of Hardy-Weinberg equilibrium ($P < 0.001$). Only SNPs fulfilling the above mentioned QC were used in subsequent steps. Genotypes of cases and controls were recoded to the “+” using the –flip

command from PLINK.[24] In addition, markers were excluded if: i) 3 alleles were detected, ii) the allele frequency in the SHIP control population differed by more than 0.1 compared with the frequency in 379 Europeans available from the 1000 Genomes project. Haplotype phasing and imputation in the HRC panel version1.1 was performed using the University of Michigan server.[7] Variants with an imputation quality score of $r^2 < 0.5$ or out of Hardy-Weinberg-Equilibrium $P < 10^{-12}$ were excluded. Principal component (PC) analysis was performed with EIGENSTRAT (SMARTPCA).

Statistical analysis

A score test implemented in RVTESTS[8] was used to test the association between SNP allelic dosage and eczema status. Sex and the first 2 principle components were included as covariates.

Acknowledgements and funding

We thank all individuals and families for their participation in this study. We thank all physicians and nurses involved in patient recruitment for their valuable contribution to the study. We are grateful to the laboratory technicians Christina Flachmeier and Theresa Thuß for their excellent technical assistance. The study was funded by the German Ministry of Education and Research (BMBF) through the Clinical Research Group for Allergy at Charité Berlin, the National Genome Research Network (NGFN). The SHIP authors are grateful to Mario Stanke for the opportunity to use his server cluster for SNP imputation. We thank all staff members and participants of the SHIP studies, as well as all of the genotyping staff for generating the SHIP SNP data set. SHIP is part of the Community Medicine Research net of the University of Greifswald, Germany, which is funded by the Federal Ministry of Education and Research (grants no. 01ZZ9603, 01ZZ0103, and 01ZZ0403), the Ministry of Cultural Affairs as well as the Social Ministry of the Federal State of Mecklenburg-West Pomerania, and the network 'Greifswald Approach to individualized Medicine (GANI_MED)' funded by the Federal Ministry of Education and Research (grant 03IS2061A). Genome-wide data were supported by the Federal Ministry of Education and Research (grant 03ZIK012) and a joint grant from Siemens Healthcare, Erlangen, Germany, and the Federal State of Mecklenburg–West Pomerania. The University of Greifswald is a member of the 'Center of Knowledge interchange' program of the Siemens AG and the Caché Campus program of the interSystems GmbH.

GEnetic analysis of NUclear Families with Atopic Dermatitis / Study of Health in Pomerania - 2 (GENUFAD / SHIP2)

Recruitment and phenotype definition

All atopic dermatitis patients were recruited at Charité Universitätsmedizin Berlin for the GENUFAD study (GEnetic analysis of NUclear Families with Atopic Dermatitis) and have been previously described in a previous GWAS[21,22] (Set 2 in original study). 270 German families were recruited through two affected siblings with an age of onset below two years of age and moderate to severe disease expression. One affected child was selected from each family and 259 atopic dermatitis patients were included in the present study.

All controls originated from the population-based Study of Health in Pomerania (SHIP)[23], which recruited individuals in the North-Eastern part of Germany. The SHIP set was split for two case-control studies by a random function. 1,792 unrelated individuals were included in SHIP-2.
All cases had an early onset (< 2years) physician's diagnosis of AD made according to standard criteria[16]. Controls were unrelated individuals from the population-based SHIP cohort.

Genotyping and imputation

All cases were genotyped with Affymetrix 500K arrays and only samples with high call rate (> 0.95) were used for the analysis. Controls were genotyped with Affymetrix Genome-Wide Human SNP Array 6.0 and were excluded when call rate < 0.96. For both case and control groups, samples were excluded when the gender estimated from X-chromosome heterozygosity did not match the clinical records. SNPs from the 500K array were filtered as previously described41 according to the following criteria: i) low call rate (< 0.95), ii) low allele frequency (MAF < 0.01), iii) Mendelian errors in 5 or more families, iv) unlikely genotypes in more than 5 families (double recombinants as detected by Merlin44), v) founder genotypes out of Hardy-Weinberg equilibrium ($P < 0.00001$). Additionally, SNPs with a call rate < 0.99 were excluded if having MAF < 0.05 or if they were out of Hardy-Weinberg equilibrium ($P < 0.001$). SNPs on the Human SNP Array 6.0 were excluded if having: i) low call rate (< 0.97), ii) low allele frequency (MAF < 0.01), iii) founder genotypes out of Hardy-Weinberg equilibrium ($P < 0.0005$). Additionally SNPs with a call rate < 0.99 were excluded if having MAF < 0.05 or if they were out of Hardy-Weinberg equilibrium ($P < 0.001$). Only SNPs fulfilling the above mentioned QC on both arrays were used in subsequent steps, and the rest of non-overlapping SNPs were excluded. Genotypes of cases and controls were recoded to the "+" using the –flip command from PLINK[24] and merged. Additionally, markers were excluded if: i) 3 alleles were detected, ii) the allele frequencies in the SHIP control population differed by more than 0.1 compared to the 379 Europeans available from the 1000 Genomes project. Haplotype phasing and imputation in the HRC panel version1.1 was performed using the University of Michigan server.[7] Variants with an imputation quality score of $r^2 < 0.5$ or out of Hardy-Weinberg-Equilibrium $P < 10^{-12}$ were excluded. Principal component (PC) analysis was performed with EIGENSTRAT (SMARTPCA).[25]

Statistical analysis

A score test implemented in RVTESTS[8] was used to test the association between SNP allelic dosage and eczema status. Sex and the first 2 principle components were included as covariates.

Acknowledgements and funding

We thank all individuals and families for their participation in this study. We thank all physicians and nurses involved in patient recruitment for their valuable contribution to the study. We are grateful to the laboratory technicians Christina Flachmeier and Theresa Thuß for their excellent technical assistance. The study was funded by the German Ministry of Education and Research (BMBF) through the Clinical Research Group for Allergy at Charité Berlin, the National Genome Research Network (NGFN). The SHIP authors are grateful to Mario Stanke for the opportunity to use his server cluster for SNP imputation. We thank all staff members and participants of the SHIP studies, as well as all of the genotyping staff for generating the SHIP SNP data set. SHIP is part of the Community Medicine Research net of the University of Greifswald, Germany, which is funded by the Federal Ministry of Education and Research (grants no. 01ZZ9603, 01ZZ0103, and 01ZZ0403), the Ministry of Cultural Affairs as well as the Social Ministry of the Federal State

of Mecklenburg-West Pomerania, and the network 'Greifswald Approach to individualized Medicine (GANI_MED)' funded by the Federal Ministry of Education and Research (grant 03IS2061A). Genome-wide data were supported by the Federal Ministry of Education and Research (grant 03ZIK012) and a joint grant from Siemens Healthcare, Erlangen, Germany, and the Federal State of Mecklenburg–West Pomerania. The University of Greifswald is a member of the 'Center of Knowledge interchange' program of the Siemens AG and the Caché Campus program of the interSystems GmbH.

GINI / LISA

Recruitment and phenotype definition

The influence of Life-style factors on the development of the Immune System and Allergies in East and West Germany (LISA) study is a population based birth cohort study. A total of 3,094 healthy, full-term neonates were recruited between 1997 and 1999 in Munich, Leipzig, Wesel and Bad Honnef. The participants were not pre-selected based on family history of allergic diseases.[26]

A total of 5,991 mothers and their newborns were recruited into the German infant study on the influence of Nutrition intervention PLUS environmental and genetic influences on allergy development (GINIplus) between September 1995 and June 1998 in Munich and Wesel. Infants with at least one allergic parent and/or sibling were allocated to the interventional study arm investigating the effect of different hydrolysed formulas for allergy prevention in the first year of life.[27] All children without a family history of allergic diseases and children whose parents did not give consent for the intervention were allocated to the non-interventional arm. Detailed descriptions of the LISA and GINIplus studies have been published elsewhere.[26,27] DNA was collected at the age 6 and 10 years. For both studies, approval by the local Ethics Committees and written consent from participant's families were obtained.

information on ever having physician-diagnosed atopic eczema was collected using self-administered questionnaires completed by the parents. The questionnaires were completed at 6, 12, 18 and 24 months and 4, 5, 6, 10 years of age in the LISA study and 1, 2, 3, 4, 6 and 10 years in the GINIplus study, asking for each year of age since the previous follow-up. Cases were defined as subjects who reported having a diagnosis at any time point, and controls were defined as those reporting no diagnosis at every time point.

Genotyping and imputation

1,511 children from Munich from both studies were included (835 (55%) children from the GINIplus study and 676 (45%) children from the LISA study). 1423 individuals (835 from the GINIplus study and 588 from the LISA study) were analyzed using the Affymetrix Human SNP Array 5.0 and 88 individuals from the LISA study were analyzed using Affymetrix Human SNP Array 6.0. Genotypes were called using BRLMM-P algorithm (5.0), respectively BIRDSEED V2 algorithm (6.0). In each of the two data sets, criteria for exclusion of individuals were: a call rate below 95%, heterozygosity outside mean +/- 4sd, a failure of the sex check or a failure of the similarity quality control using MDS analysis based on IBS. Criteria for exclusion of variants were: a call rate below 95%, a MAF < 0.01 and a HWE P value < 0.00001. Haplotype phasing and imputation in the HRC panel version1.1 was performed using the University of Michigan

server.[7] Variants with an imputation quality score of $r^2 < 0.5$ or out of Hardy-Weinberg-Equilibrium $P < 10^{-12}$ were excluded.

Statistical analysis

A score test implemented in RVTESTS[8] was used to test the association between SNP allelic dosage and eczema status. Sex was included as a covariate.

Acknowledgements and funding

The authors thank all families for participation in the studies and the LISA and GINIplus study teams for their excellent work.

INfancia y Medio Ambiente Project (INMA)

Recruitment and phenotype definition

Population-based birth cohorts were established as part of the INMA – INfancia y Medio Ambiente [Environment and Childhood] Project in several regions of Spain following a common protocol. This project aims to study the associations between pre- and postnatal environmental exposures and growth, health, and development from early fetal life until adolescence and has been described previously in detail.[28] Pregnant women were enrolled during the 1st trimester of pregnancy at public primary health care centers or public hospitals. Detailed measurements were performed using ultrasound and physical examinations and biological samples were collected. Informed consent was obtained from all participants and the study was approved by the Hospital Ethics Committees in each participating region.

Children from the subcohorts of INMA Sabadell, Valencia and Menorca were included in the present study. Atopic eczema (Sabadell and Valencia) and doctor atopic eczema (Menorca) was assessed by questionnaire at the ages of 1, 2 and 4y. Atopic eczema cases were those children that had had eczema at least in one of the three visits. Control children were those that had never had eczema.

Genotyping and imputation

DNA was obtained from cord blood, whole blood collected at 4y or saliva using the Chemagen protocol at the Spanish National Genotyping Centre (CEGEN). Children whose parents reported to be white and to be born in Spain or in European countries and that were not lost during the follow-up were selected for genotyping. Genome-wide genotyping was performed using the HumanOmni1-Quad Beadchip (Illumina) at CEGEN. Genotype calling was done using the GeneTrain2.0 algorithm based on HapMap clusters implemented in the GenomeStudio software. Quality control was done using PLINK[24] and following standard criteria. First of all, SNPs were flipped to the human genome + strand. We applied the following initial quality control thresholds: sample call rate>98% and/or LRR SD< 0.3. Then, we checked sex, relatedness, heterozygosity and population stratification. Genetic variants were filtered for SNP call rate>95%, MAF>1% and HWE P value>1.1×10^{-6}. Haplotype phasing and imputation in the HRC panel version1.1 was performed using the University of Michigan server.[7] Variants with an imputation quality score of $r^2 < 0.5$ or out of Hardy-Weinberg-Equilibrium $P < 10^{-12}$ were excluded.

Statistical analysis

A score test implemented in RVTESTS[8] was used to test the association between SNP allelic dosage and eczema status. Sex and the first two principle components were included as covariates.

Acknowledgements and funding

This study was funded by grants from instituto de Salud Carlos III (CB06/02/0041, G03/176, FIS PI041436, PI081151, PI041705, PI061756, PI091958, and PS09/00432, FIS-FEDER 03/1615, 04/1509, 04/1112, 04/1931 , 05/1079, 05/1052, 06/1213, 07/0314, 09/02647, 11/01007, 11/02591, 11/02038, 13/1944, 13/2032 and CP11/0178), Spanish Ministry of Science and innovation (SAF2008-00357), European Commission (ENGAGE project and grant agreement HEALTH-F4-2007-201413, HEALTH.2010.2.4.5-1, FP7-ENV-2011 cod 282957), Fundació La Marató de TV3, Generalitat de Catalunya-CIRIT 1999SGR 00241 and Conselleria de Sanitat Generalitat Valenciana. Part of the DNA extractions and genotyping was performed at the Spanish National Genotyping Centre (CEGEN-Barcelona). The authors are grateful to Silvia Fochs, Anna Sànchez, Maribel López, Nuria Pey, Muriel Ferrer, Amparo Quiles, Sandra Pérez, Gemma León, Elena Romero, Maria Andreu, Nati Galiana, Maria Dolores Climent, Amparo Cases and Cristina Capo for their assistance in contacting the families and administering the questionnaires. A full roster of the INMA Project investigators can be found at http://www.proyectoinma.org/presentacion-inma/listado-investigadores/en_listado-investigadores.html. The authors would particularly like to thank all the participants for their generous collaboration.

Manchester Asthma and Allergy Study (MAAS)

Recruitment and phenotype defintion

The Manchester Asthma and Allergy Study is an unselected (i.e. population-based), birth cohort study.[29-33] The setting is the maternity catchment area of Wythenshawe and Stepping Hill Hospitals, comprising of 50 square miles of South Manchester and Cheshire, UK, a stable mixed urban-rural population. Study was approved by the Local Research Ethics Committee. Informed consent was obtained from all parents.

Screening & Recruitment

All pregnant women were screened for eligibility at antenatal visits (8th-10th week of pregnancy). The study was explained to the parents, and informed consent for initial questionnaires and skin prick testing was obtained. Both parents completed a questionnaire about their and their partner's history of asthma and allergic diseases and smoking habits. If the pregnant woman's partner was not present at the antenatal clinic visit, an invitation was sent for him to attend an open-access evening clinic for skin prick testing and questionnaire. Once both parents had completed questionnaires and skin prick testing, a full explanation of the proposed future follow-up for the child was given. Of the 1,499 couples who met the inclusion criteria (< 10 weeks of pregnancy, maternal age >18 years, questionnaire and skin test data available for both parents), 288 declined to take part in the study. A total of 1,185 participants had at least some evaluable data.

Follow-up

The children have been followed prospectively, and attended review clinics at ages 1, 3, 5, 8 and 11 years.

Cases were defined as a doctor diagnosis of atopic dermatitis on the day that the child came to the follow-up clinic, at any timepoint (1y, 3y, 5y, 8y)
Controls were a Parental report of “no” at all timepoints (1y, 3y, 5y, 8y) to the question "Has your child ever suffered from atopic dermatitis", ‘No’

Genotyping and imputation

DNA samples were genotyping on an Illumina 610 quad chip. The Illumina genotypes were called using the Illumina GenCall application following the manufacturer’s instructions. Quality control criteria for samples included: 97% call rate, exclusion of samples with an outlier autosomal heterozygosity (scree-plot visualization) gender validation and sequenome genotype concordance. Quality control criteria for SNPs included a 95% call rate, HWE > $5.9x10^{-7}$, minor allele frequency > 0.005. Haplotype phasing and imputation in the HRC panel version1.1 was performed using the University of Michigan server.[7] Variants with an imputation quality score of $r^2 < 0.5$ or out of Hardy-Weinberg-Equilibrium $P < 10^{-12}$ were excluded.

Statistical analysis

A score test implemented in RVTESTS[8] was used to test the association between SNP allelic dosage and eczema status. Sex and the first two principle components were included ascovariates.

Acknowledgements and funding

We would like to thank the children and their parents for their continued support and enthusiasm. We greatly appreciate the commitment they have given to the project. We would also like to acknowledge the hard work and dedication of the study team (post-doctoral scientists, research fellows, nurses, physiologists, technicians and clerical staff). MAAS was supported by the Asthma UK Grants No 301 (1995-1998), No 362 (1998-2001), No 01/012 (2001-2004), No 04/014 (2004-2007), the James Trust and Medical Research Council, UK (G0601361) and The Moulton Charitable Foundation (2004-current); age 11 years clinical follow-up is funded by the Medical Research Council (MRC) Grant G0601361.

Multicentre Allergy Study / Heinz Nixdorf RECALL (MAS / HNR)

Recruitment and phenotype definition

The Multicentre Allergy Study (MAS) is a German birth cohort which has been described in detail previously.[34,35] Briefly, in 1990, the MAS cohort recruited 1,314 newborns, who were regularly followed-up. Data were collected from examinations and questionnaires at birth, at 1, 3, 6, 12, and 18 months, and yearly from age 2 to age 13. Samples included in this study participated in the GWAS on asthma of the GABRIEL consortium.[36] German controls are from the population-based Heinz Nixdorf RECALL (HNR) study which randomly selected 4,200 individuals in the Western part of Germany in order to study atherosclerotic disease.[37]
Eczema was defined based on a parental report of a doctor’s diagnosis of eczema up to the age of 13 years. Controls were unrelated individuals from the population-based HNR study.

Genotyping and imputation

MAS samples were genotyped with the Illumina Human610 array, HNR samples with the Illumina Human550v3 array. Individuals with a call rate < 0.95 or with high heterozygosity (> 0.35) were excluded. SNPs were filtered according to the following criteria: i) low call rate (<

0.95 in cases or controls), ii) low allele frequency (MAF < 0.01 in cases or controls), iii) genotypes out of Hardy-Weinberg equilibrium ($P < 0.00001$ in cases or $P < 0.0005$ in controls). Additionally, SNPs with a call rate < 0.99 were excluded if having a MAF < 0.05 or if they were out of Hardy-Weinberg equilibrium ($P < 0.001$). Only SNPs fulfilling the above mentioned QC were used in subsequent steps. Genotypes of cases and controls were recoded to the "+" using the –flip command from PLINK.[24] Additionally, markers were deleted if: i) 3 alleles were detected, ii) the allele frequencies in the HNR control population differed by more than 0.15 compared with the frequency in 379 Europeans available from the 1000 Genomes project. After filtering, 514680 SNPs in 104 cases and 379 controls remained in the analysis. Haplotype phasing and imputation in the HRC panel version1.1 was performed using the University of Michigan server.[7] Variants with an imputation quality score of $r^2 < 0.5$ or out of Hardy-Weinberg-Equilibrium $P < 10^{-12}$ were excluded. Principal component (PC) analysis was performed with EIGENSTRAT (SMARTPCA).[25]

Statistical analysis

A score test implemented in RVTESTS[8] was used to test the association between SNP allelic dosage and eczema status. Sex and the first two principle components were included as covariates.

Acknowledgements and funding

We are grateful to all children and parents who participated in this study. Collaborators of the MAS group: R. Bergmann (Berlin, Germany) V. Wahn, M. Groeger (Dusseldorf, Germany); J. Forster, U. Tacke (Freiburg, Germany); C-P. Bauer (Gaisach, Germany); F. Zepp, I. Bieber (Mainz, Germany). This study was supported by the German Ministry of Education and Research (BMBF) through grants number 01 EE9406 and 01 GC9702/0. Genotyping of the MAS cases was supported through the EU Framework 6 integrated Project "GABRIEL ". The collection of probands in the Heinz Nixdorf RECALL Study (HNR) was supported by the Heinz Nixdorf Foundation. The genotyping of HNR probands was financed through a grant of the German Ministry of Education and Science (BMBF). MMN is member of the Excellence Cluster ImmunoSensation2 which is funded by the DFG under Germany's Excellence Strategy – EXC2151 (project number 390873048).

Northern Finland Birth Cohort 1966 (NFBC66)

Recruitment and phenotype definition

The Northern Finland Birth Cohort 1966 is a prospective follow-up study of children from the two northernmost provinces of Finland.[38] Women with expected delivery dates in 1966 were recruited through maternity health centers.[39] Cohort members living in northern Finland or in the capital area were invited to a clinical examination as well as questionnaire at age 31 years. DNA was extracted from blood samples given at the clinical examination.[40] Informed consent for the use of the data including DNA was obtained from all subjects. The study was approved by the ethics committees in Oulu (Finland) and Oxford (UK) universities in accordance with the Declaration of Helsinki.

For the purpose of this meta-analysis, we included data from the following questions:

1. Have you had eczema (infantile, atopic or allergic)?

2. If yes, have you ever been treated by a doctor

individuals who answered yes to both questions were defined as cases (1,200). Individuals that answered no to the first question were defined as controls (2,270).

Genotyping and imputation

Genotyping was completed at the Broad Institute Biological Sample Repository in participants with available DNA using Illumina HumanCNV370DUO Analysis BeadChip array for 339,629 SNPs. We excluded 3,345 SNPs from analysis because HWE was not met at a level $P < 0.0001$, 55 because of low call rate (< 95%) and 7,681 because the MAF was < 1% as well as SNPs with duplicates concordance < 99% leaving 309, 948 SNPs for the association analysis. Individuals with IBS pairwise sharing>0.20, that withdrew consent, with gender mismatch, that are heterozygosity outliers 0.29< F< 0.35, and MDS outliers were also removed. Haplotype phasing and imputation in the HRC panel version1.1 was performed using the University of Michigan server.[7] Variants with an imputation quality score of $r^2 < 0.5$ or out of Hardy-Weinberg-Equilibrium $P < 10^{-12}$ were excluded.

Statistical analysis

A score test implemented in RVTESTS[8] was used to test the association between SNP allelic dosage and eczema status. Sex and the first three principle components were included as covariates.

Acknowledgements and funding

We thank the late Professor Paula Rantakallio (launch of NFBC1966), and Ms Outi Tornwall and Ms Minttu Jussila (DNA biobanking). The authors would like to acknowledge the contribution of the late Academian of Science Leena Peltonen.

NFBC1966 received financial support from the Academy of Finland (project grants 104781, 120315, 129269, 1114194, 24300796, Center of Excellence in Complex Disease Genetics and SALVE), Oulu University Hospital, Finland, Biocenter, University of Oulu, Finland 75617, 24002054, University of Oulu, Finland (Grant no. 24000692 and 24500283:Well-being and health: Research in the Northern Finland Birth Cohorts 1966 and 1986, Phenotypic and Genomic analyses). NIH/NHLBI NHLBI grant 5R01HL087679-02 through the STAMPEED program (1RL1MH083268-01), NHLBI Consortium for Neuropsychiatric Phenomics Co-ordinating Center (1-R01-HL087679-01) and. NIH/NIMH (5R01MH63706:02), USA. ENGAGE project and grant agreement HEALTH-F4-2007-201413. Medical Research Council (grant no. G1002319).The DNA extractions, sample quality controls, biobank up-keeping and aliquotting was supported financially by the Academy of Finland and Biocentrum Helsinki.

Prevention and Incidence of Asthma and Mite Allergy birth cohort (PIAMA)

Recruitment and phenotype definition

PIAMA is a birth cohort study consisting of two parts: a placebo controlled intervention study in which the effect of mite impermeable mattress covers on the development of asthma and allergy was studied and a natural history study in which no intervention took place. Details of the study design have been published previously.[41] Recruitment took place in 1996-1997 through prenatal clinics. A screening questionnaire was distributed to pregnant women visiting one of 52 prenatal clinics at three regions in the Netherlands. A total of 10,232 pregnant

women completed a validated screening questionnaire. Mothers reporting a history of asthma, current hay fever or allergy to pets or house dust mite were defined as allergic. Based on this screening, 7,862 women were invited to participate, of whom 4,146 women (1,327 allergic and 2,819 non-allergic) gave written informed consent. Follow-up of the children took place at 3 months of age and yearly from 1 to 8 years of age. The Medical Ethical Committees of the participating institutes approved the study, and all participants gave written informed consent. Questionnaire information on eczema was obtained at ages 3m,1y,2y,3y,4y,5y,6y,7y,8y. Cases were defined as a positive response to one or more of these three questions:
1. Has your child ever had atopic dermatitis? 2. Did a doctor ever diagnose atopic dermatitis in your child? And did your child have atopic dermatitis during the past 12 months?
Controls were defined a negative response to these questions at ages 2 – 8 years.

Genotyping and imputation

DNA was collected from 2,162 children. Genome-wide genotyping was performed in three phases. The first phase was performed within the framework of the GABRIEL Consortium using an Illumina Human 610K quad array.[36] Genotypes were available from 172 children with asthma and from 187 controls after quality control. A second group of 268 children who were more extensively examined during follow up was genotyped with an Illumina HumanOmniExpress array. A final group of 1,377 children was genotyped with the Illumina Human Omni Express Exome Array. SNPs were harmonized by base pair position annotated to genome build 37, name and annotation of strand for each platform. Discordant or duplicate SNPs or SNPs that showed large differences in allele frequencies (> 15 %) were removed. After quality control, a total of 1,968 individuals remained. Haplotype phasing and imputation in the HRC panel version1.1 was performed using the University of Michigan server.[7] Variants with an imputation quality score of $r^2 < 0.5$ or out of Hardy-Weinberg-Equilibrium $P < 10^{-12}$ were excluded.

Statistical analysis

A score test implemented in RVTESTS[8] was used to test the association between SNP allelic dosage and eczema status. Sex and the first two principle components were included as covariates.

Acknowledgements and funding

The PIAMA birth cohort study is a collaboration of the Institute for Risk Assessment Sciences, University Utrecht (B. Brunekreef), Julius Center for Health Sciences and Primary Care, University Medical Center Utrecht (H.A. Smit), Centre for Prevention and Health Services Research, National Institute for Public Health and the Environment, Bilthoven (A.H. Wijga), Department of Pediatrics, Division of Respiratory Medicine, Erasmus MC -Sophia, Rotterdam (J.C. de Jongste), Pulmonology (D.S. Postma) and Pediatric Pulmonology and Pediatric Allergology (G.H. Koppelman) of the University Medical Center Groningen and the Department of Immunopathology, Sanquin Research, Amsterdam (R.C. Aalberse), The Netherlands. The study team gratefully acknowledges the participants in the PIAMA birth cohort study, and all coworkers who helped conducting the medical examinations, field work and data management. We specifically thank O. Savenije, F.N. Dijk, I. Nolte and P. van der Vlies for their support. The PIAMA study was funded by grants from the Dutch Asthma Foundation (grant 3.4.01.26, 3.2.06.022, 3.4.09.081 and 3.2.10.085CO), the ZON-MW Netherlands Organization for Health Research and Development (grant 912-03-031), the Stichting Astmabestrijding and the Ministry of the Environment. Genome-wide genotyping was funded by the European Commission as part

of GABRIEL (A multidisciplinary study to identify the genetic and environmental causes of asthma in the European Community) contract number 018996 under the integrated Program LSH-2004-1.2.5-1 Post genomic approaches to understand the molecular basis of asthma aiming at a preventive or therapeutic control and a Grant from BBMRI-NL (CP 29)

The Raine Study

<u>Recruitment and phenotype definition</u>

The Raine Study[42] is a prospective pregnancy cohort where 2900 were recruited from King Edward Memorial Hospital between 1989 and 1991. Data were collected throughout pregnancy and the children have been followed-up at ages 1, 2, 3, 5, 8, 10, 14, 17, 18, 20, and 22. Ethics approval for this study was obtained from King Edward Memorial Hospital and Princess Margaret Hospital. Participants were consented to being involved in this study prior to each follow-up.

The children have been followed up with regular questionnaires and clinic visits. For the current study data collected from the questionnaires was used to classify children as eczema cases or controls. When the children were approximately 5, 13 and 16 years, parents were asked the following questions:

1. Has anyone ever told you that your child has eczema? If yes, who told you your child has eczema?

We defined cases as the children of parents who answered ‘Yes and was diagnosed by a doctor or pediatrician’ at any one of the follow-ups. Controls were defined as children of parents who answered no at all 3 follow-ups.

<u>Genotyping and imputation</u>

The GWAS data was genotyped in two separate lots (dependent on when the subjects DNA was processed and ready to genotype) using the Illumina Human660W Quad Array at the Centre for Applied Genomics (Toronto, Ontario, Canada). The first round of genotyping was completed on 1,259 Raine Study children (including 63 replicates and a plate control on each plate) and the second on 334 children (including 18 replicates and a plate control on each plate). The 660W Quad Array includes 657,366 genetic variants including ~560,000 single nucleotide polymorphisms (SNPs) and ~95,000 copy number variants (CNV’s).

All individuals of non-European ancestry, ambiguous sex, extreme heterozygosity (< 0.3), cryptic relatedness (π>0.1875) and high missingness (>3%) were removed. SNPs with low genotyping rate (< 95%), with low minor allele frequency (< 1%), out of Hardy Weinberg equilibrium ($p < 5x10^{-7}$) were excluded. 1494 individuals typed on 535,632 probes remained. Haplotype phasing and imputation in the HRC panel version1.1 was performed using the University of Michigan server.[7] Variants with an imputation quality score of $r^2 < 0.5$ or out of Hardy-Weinberg-Equilibrium $P < 10^{-12}$ were excluded.

<u>Statistical analysis</u>

A score test implemented in RVTESTS[8] was used to test the association between SNP allelic dosage and eczema status. Sex and the first two principle components were included as covariates.

<u>Acknowledgements and funding</u>

This study was supported by the National Health and Medical Research Council of Australia [grant numbers 403981 and 003209] and the Canadian Institutes of Health Research [grant number MOP-82893]. The authors are grateful to the Raine Study participants and their families, and to the Raine Study research staff for cohort coordination and data collection. The authors gratefully acknowledge the NH&MRC for their long term contribution to funding the study over the last 30 years and also the following institutions for providing funding for Core Management of the Raine Study: The University of Western Australia (UWA), Raine Medical Research Foundation, Telethon Kids Institute, Women and infants Research Foundation (King Edward Memorial Hospital), Murdoch University, The University of Notre Dame (Australia) and Edith Cowan University. The authors gratefully acknowledge the assistance of the Western Australian DNA Bank (National Health and Medical Research Council of Australia National Enabling Facility).

This work was supported by resources provided by the Pawsey Supercomputing Centre with funding from the Australian Government and the Government of Western Australia

SALTY

Recruitment and phenotype definition

The study participants had participated in a telephone interview called Screening Across the Lifespan Twin Study (SALT), conducted between 1998 and 2002. The target population for SALTY was the younger part of the SALT cohort born between 1943 and 1958. The data collection consisted of three parts: (1) an extensive self-report paper-questionnaire; (2) saliva collection for DNA extraction; and (3) a request to participate in an internet-based investigation. Saliva samples were collected from the study participants either by mail in connection with invitation to the study. Some of the participants in SALTY were also prior participants of TwinGene – if they had already provided a blood sample they were not also asked to provide saliva. Case-control definition for eczema followed the same approach described for the CATSS study described above.

Genotyping and imputation

As described above for CATSS.

Statistical analysis

As described above for CATSS.

Acknowledgment and funding

As described above for CATSS.

TWINS UK

Recruitment and phenotype definition

The St Thomas's UK Adult Twin Registry includes 14,000 mainly female twins from throughout the United Kingdom and who are unselected for any diseases or traits. Pairs are invited to attend regular clinical visits at the Twin Research Unit, St Thomas's Hospital, London, for a full day of clinical tests, including a self-completed questionnaire relating to allergic diseases.

Volunteers twins were asked "Have you ever had eczema?" or "Has a doctor ever told you that you have eczema” on multiple occasions at different time points. Cases were determined from those people who had consistently answered positively in one or more occasions. Only female twins participated to this study.

The study was approved by the Local Research Ethics Committee of St Thomas's Hospital, and subjects gave full informed consent.

Genotyping and imputation

Genotyping of the TwinsUK dataset was done with a combination of Illumina arrays (HumanHap300, HumanHap610Q, 1M-Duo and 1.2MDuo 1M). Intensity data for each of the three arrays were pooled separately (with 1M-Duo and 1.2MDuo 1M pooled together) and genotypes were assigned using the Illuminus calling algorithm. We applied similar quality control criteria to each dataset and merged them. Haplotype phasing and imputation in the HRC panel version1.1 was performed using the University of Michigan server.[7] Variants with an imputation quality score of r2 < 0.5 or out of Hardy-Weinberg-Equilibrium $P < 10^{-12}$ were excluded.

Statistical analysis

A score test implemented in RVTESTS[8] was used to test the association between SNP allelic dosage and eczema status. The first four principle components were included as covariates.

Acknowledgements and funding

TwinsUK was funded by the Wellcome Trust and MRC. The study also receives support from the National Institute for Health Research (NIHR)- funded BioResource, Clinical Research Facility and Biomedical Research Centre based at Guy’s and St Thomas’ NHS Foundation Trust in partnership with King’s College London. We gratefully acknowledge support provided by the JPI HDHL funded DINAMIC consortium (administered by the MRC UK, MR/N030125/1).

FINNGEN

Recruitment and phenotype definition

FinnGen is a nation-wide study launched in Finnland in 2017 which combines genome information with digital health care data (https://finngen.gitbook.io/documentation/). Phenotypes were derived from ICD codes in Finnish national hospital registries. We downloaded the summary statistics for the phenotype L20 Atopic dermatitis, totaling 2663 cases and 88760 controls, from the publicly available FinnGen data release 2.

Genotyping and imputation

FinnGen samples were genotyped with Illumina (Illumina Inc., San Diego, CA, USA) and Affymetrix arrays (Thermo Fisher Scientific, Santa Clara, CA, USA) and imputed as previously described.[43] Genotype calls were made with GenCall and zCall algorithms for Illumina and AxiomGT1 algorithm for Affymetrix chip genotyping data. Genotyping data produced with previous chip platforms were lifted over to build version 38 (GRCh38/hg38) following the protocol described here: dx.doi.org/10.17504/protocols.io.nqtddwn. Samples with sex discrepancies, high genotype missingness (>5%), excess heterozygosity (±4SD) and non-Finnish ancestry were removed. Variants with high missingness (>2%), deviation from HWE ($P < 1e-6$)

and low minor allele count (MAC<3) were removed. Pre-phasing of genotyped data was performed with Eagle 2.3.5[44] (https://data.broadinstitute.org/alkesgroup/Eagle/) with the default parameters, except the number of conditioning haplotypes was set to 20,000. Imputation was carried out by using the populationspecific SISu v3 imputation reference panel with Beagle 4.1[45] (https://faculty.washington.edu/browning/beagle/b4_1.html) as described in the following protocol: dx.doi.org/10.17504/protocols.io.nmndc5e. SISu v3 imputation reference panel was developed using the high-coverage (25-30x) whole genome sequencing data generated at the Broad Institute of MIT and Harvard and at the McDonnell Genome Institute at Washington University; and jointly processed at the Broad Institute. Variant callset was produced with GATK HaplotypeCaller algorithm by following GATK best-practices for variant calling. Genotype-, sample- and variant-wise QC was applied in an iterative manner by using the Hail framework v0.1 (https://github.com/hail-is/hail). The resulting high-quality WGS data for 3,775 individuals were phased with Eagle 2.3.5 as described above. Post-imputation quality control involved excluding variants with INFO score < 0.7.

<u>Statistical analysis</u>

GWAS was performed with SAIGE, a mixed model logistic regression R/C++ package. Sex, age, 10 PCs, and genotyping batch were included as covariates.

<u>Acknowledgements</u>

We want to acknowledge the participants and investigators of FinnGen study.

UK Biobank (UKBB)

<u>Recruitment and phenotype definition</u>

The UK Biobank is a large prospective study established by the Medical Research Council, Department of Health, Wellcome Trust, Scottish government and North-West Regional Development Agency. The study was conducted with the approval of the North-West Research Ethics Committee (Reference: 06/MRE08/65). Genotyped and imputation data for the near 500,000 individuals from UKBB were made available to the researchers together with primary genotype quality controls and analyses such as ancestry grouping and detection of close kinship.

Participants from white British ancestry were selected as defined in the UKBB pipeline.[46] For the definition of the eczema status in UKBB, we combined information from three sources as described previosly:[47] (1) touchscreen questionnaire (data-field 6152: has your doctor ever diagnosed you with eczema, allergic rhinitis or hayfever?); (2) verbal interview (data-field 20002: non-cancer illness code, self-reported eczema/dermatitis); (3) main (data-field 41202) and secondary (data-field 41204) ICD10 diagnoses. Inclusion criteria for cases were: (i) a report of eczema/allergic rhinitis/hay fever in field 6152 AND a code for eczema/dermatitis (1452) in field 20002; or (ii) an ICD10 code for eczema in fields 41202 or 41204, including L20.8 or L20.9. Inclusion criteria for controls were no report of eczema in fields 6152, 20002, 41202 or 41204. Variant quality control involved removing SNPs with INFO score <0.8 and MAF<0.001.

<u>Genotyping and imputation</u>

As mentioned previously, genotyping and imputation data were provided by UKBB. More information can be found in Bycroft et al. (2018).[46]

Statistical analysis

GWAS was performed for unrelated white British using PLINK2.048 (www.cog-genomics.org/plink/2.0/) and a logistic regression analysis. Sex, age and 20 PCs were added as covariates.

Acknowledgements

This research has been conducted using the UK Biobank Resource under project 788. The work was funded by Roslin Institute Strategic Programme Grants from the BBSRC (BBS/E/D/10002070 and BBS/E/D/30002275) and Health Data Research UK (references HDR-9004 and HDR-9003).

Supplementary References

1. Purcell, S., Cherny, S.S. & Sham, P.C. Genetic Power Calculator: design of linkage and association genetic mapping studies of complex traits. *Bioinformatics* **19**, 149-50 (2003).
2. de Leeuw, C.A., Mooij, J.M., Heskes, T. & Posthuma, D. MAGMA: generalized gene-set analysis of GWAS data. *PLoS Comput Biol* **11**, e1004219 (2015).
3. Watanabe, K., Taskesen, E., van Bochoven, A. & Posthuma, D. Functional mapping and annotation of genetic associations with FUMA. *Nat Commun* **8**, 1826 (2017).
4. Cole, C. *et al.* Filaggrin-stratified transcriptomic analysis of pediatric skin identifies mechanistic pathways in patients with atopic dermatitis. *J Allergy Clin Immunol* **134**, 82-91 (2014).
5. GTEx Consortium. Human genomics. The Genotype-Tissue Expression (GTEx) pilot analysis: multitissue gene regulation in humans. *Science* **348**, 648-60 (2015).
6. Ferreira, M.A.R. *et al.* Identification of IL6R and chromosome 11q13.5 as risk loci for asthma. *Lancet* **378**, 1006-1014 (2011).
7. McCarthy, S. *et al.* A reference panel of 64,976 haplotypes for genotype imputation. *Nature Genetics* **48**, 1279-83 (2016).
8. Zhan, X., Hu, Y., Li, B., Abecasis, G.R. & Liu, D.J. RVTESTS: an efficient and comprehensive tool for rare variant association analysis using sequence data. *Bioinformatics* **32**, 1423-6 (2016).
9. Boyd, A. *et al.* Cohort Profile: the 'children of the 90s'--the index offspring of the Avon Longitudinal Study of Parents and Children. *International Journal of Epidemiology* **42**, 111-27 (2013).
10. Fraser, A. *et al.* Cohort Profile: the Avon Longitudinal Study of Parents and Children: ALSPAC mothers cohort. *Int J Epidemiol* **42**, 97-110 (2013).
11. Anckarsater, H. *et al.* The Child and Adolescent Twin Study in Sweden (CATSS). *Twin Res Hum Genet* **14**, 495-508 (2011).
12. Lichtenstein, P. *et al.* The Swedish Twin Registry: a unique resource for clinical, epidemiological and genetic studies. *J Intern Med* **252**, 184-205 (2002).
13. Lichtenstein, P. *et al.* The Swedish Twin Registry in the third millennium: an update. *Twin Res Hum Genet* **9**, 875-82 (2006).

14. Magnusson, P.K. *et al.* The Swedish Twin Registry: establishment of a biobank and other recent developments. *Twin Res Hum Genet* **16**, 317-29 (2013).
15. Bisgaard, H. The Copenhagen Prospective Study on Asthma in Childhood (COPSAC): design, rationale, and baseline data from a longitudinal birth cohort study. *Ann Allergy Asthma Immunol* **93**, 381-9 (2004).
16. Hanifin, J.M. & Rajka, G. Diagnostic Features of Atopic-Dermatitis. *Acta Dermato-Venereologica* **60**, 44-47 (1980).
17. Krawczak, M. *et al.* PopGen: population-based recruitment of patients and controls for the analysis of complex genotype-phenotype relationships. *Community Genet* **9**, 55-61 (2006).
18. Wichmann, H.E., Gieger, C., Illig, T. & Group, M.K.S. KORA-gen--resource for population genetics, controls and a broad spectrum of disease phenotypes. *Gesundheitswesen* **67 Suppl 1**, S26-30 (2005).
19. Holle, R., Happich, M., Lowel, H., Wichmann, H.E. & Group, M.K.S. KORA--a research platform for population based health research. *Gesundheitswesen* **67 Suppl 1**, S19-25 (2005).
20. Williams, H.C., Burney, P.G., Strachan, D. & Hay, R.J. The U.K. Working Party's Diagnostic Criteria for Atopic Dermatitis. II. Observer variation of clinical diagnosis and signs of atopic dermatitis. *British Journal of Dermatology* **131**, 397-405 (1994).
21. Esparza-Gordillo, J. *et al.* A common variant on chromosome 11q13 is associated with atopic dermatitis. *Nature Genetics* **41**, 596-601 (2009).
22. Marenholz, I. *et al.* The eczema risk variant on chromosome 11q13 (rs7927894) in the population-based ALSPAC cohort: a novel susceptibility factor for asthma and hay fever. *Human Molecular Genetics* **20**, 2443-9 (2011).
23. Volzke, H. *et al.* Cohort profile: the study of health in Pomerania. *International Journal of Epidemiology* **40**, 294-307 (2011).
24. Purcell, S. *et al.* PLINK: a tool set for whole-genome association and population-based linkage analyses. *American Journal of Human Genetics* **81**, 559-75 (2007).
25. Patterson, N., Price, A.L. & Reich, D. Population structure and eigenanalysis. *PLoS Genet* **2**, e190 (2006).
26. Heinrich, J. *et al.* Allergens and endotoxin on mothers' mattresses and total immunoglobulin E in cord blood of neonates. *Eur Respir J* **20**, 617-23 (2002).
27. von Berg, A. *et al.* Impact of early feeding on childhood eczema: development after nutritional intervention compared with the natural course - the GINIplus study up to the age of 6 years. *Clin Exp Allergy* **40**, 627-36 (2010).
28. Guxens, M. *et al.* Cohort Profile: the INMA--INfancia y Medio Ambiente--(Environment and Childhood) Project. *Int J Epidemiol* **41**, 930-40 (2012).
29. Custovic, A. *et al.* The National Asthma Campaign Manchester Asthma and Allergy Study. *Pediatr Allergy Immunol* **13**, 32-7 (2002).
30. Lowe, L.A. *et al.* Wheeze phenotypes and lung function in preschool children. *Am J Respir Crit Care Med* **171**, 231-7 (2005).
31. Murray, C.S. *et al.* Lung function at one month of age as a risk factor for infant respiratory symptoms in a high risk population. *Thorax* **57**, 388-92 (2002).

32. Nicolaou, N.C. *et al.* Exhaled breath condensate pH and childhood asthma: unselected birth cohort study. *Am J Respir Crit Care Med* **174**, 254-9 (2006).
33. Nicolaou, N.C. *et al.* Day-care attendance, position in sibship, and early childhood wheezing: a population-based birth cohort study. *J Allergy Clin Immunol* **122**, 500-6 e5 (2008).
34. Lau, S. *et al.* Early exposure to house-dust mite and cat allergens and development of childhood asthma: a cohort study. Multicentre Allergy Study Group. *Lancet* **356**, 1392-7 (2000).
35. Nickel, R. *et al.* Messages from the German Multicentre Allergy Study. *Pediatric Allergy and Immunology* **13 Suppl 15**, 7-10 (2002).
36. Moffatt, M.F. *et al.* A large-scale, consortium-based genomewide association study of asthma. *New England Journal of Medicine* **363**, 1211-1221 (2010).
37. Schmermund, A. *et al.* Assessment of clinically silent atherosclerotic disease and established and novel risk factors for predicting myocardial infarction and cardiac death in healthy middle-aged subjects: rationale and design of the Heinz Nixdorf RECALL Study. Risk Factors, Evaluation of Coronary Calcium and Lifestyle. *American Heart Journal* **144**, 212-8 (2002).
38. Rantakallio, P. The longitudinal study of the northern Finland birth cohort of 1966. *Paediatr Perinat Epidemiol* **2**, 59-88 (1988).
39. Sovio, U. *et al.* Genetic determinants of height growth assessed longitudinally from infancy to adulthood in the northern Finland birth cohort 1966. *PLoS Genet* **5**, e1000409 (2009).
40. Frayling, T.M. *et al.* A common variant in the FTO gene is associated with body mass index and predisposes to childhood and adult obesity. *Science* **316**, 889-94 (2007).
41. Wijga, A.H. *et al.* Cohort profile: the prevention and incidence of asthma and mite allergy (PIAMA) birth cohort. *Int J Epidemiol* **43**, 527-35 (2014).
42. Newnham, J.P., Evans, S.F., Michael, C.A., Stanley, F.J. & Landau, L.I. Effects of frequent ultrasound during pregnancy: a randomised controlled trial. *Lancet* **342**, 887-91 (1993).
43. Tabassum, R. *et al.* Genetic architecture of human plasma lipidome and its link to cardiovascular disease. *Nat Commun* **10**, 4329 (2019).
44. Loh, P.R. *et al.* Reference-based phasing using the Haplotype Reference Consortium panel. *Nat Genet* **48**, 1443-1448 (2016).
45. Browning, B.L. & Browning, S.R. Genotype Imputation with Millions of Reference Samples. *Am J Hum Genet* **98**, 116-26 (2016).
46. Bycroft, C. *et al.* The UK Biobank resource with deep phenotyping and genomic data. *Nature* **562**, 203-209 (2018).
47. Ferreira, M.A. *et al.* Shared genetic origin of asthma, hay fever and eczema elucidates allergic disease biology. *Nature Genetics* **49**, 1752-1757 (2017).
48. Chang, C.C. *et al.* Second-generation PLINK: rising to the challenge of larger and richer datasets. *Gigascience* **4**, 7 (2015).
