# Supplementary material for: Rare variant analysis in eczema identifies exonic variants in DUSP1, NOTCH4 and SLC9A4
Source: Nat Commun. 2021 Nov 16;12:6618. doi: 10.1038/s41467-021-26783-x (PMC8595373; doi:10.1038/s41467-021-26783-x)
Supplement: Supplementary file 2 — Description of Additional Supplementary Files [file 41467_2021_26783_MOESM2_ESM.pdf]

Description of Additional Supplementary Files

File Name: Supplementary Data 1
Description: Characteristics of the study populations included in the meta-analysis on rare variants in eczema.

File Name: Supplementary Data 2.
Description: 48 Independent SNPs from 38 loci associated with eczema at genome-wide significance (all variants included).
SNP, single nucleotide polymorphism; Chr, chromosome; Pos, genomic positions (GRCh37.p13); Ref, reference allele; Alt, alternative allele; AF, allele frequency; OR, odds ratio.
[a] Two-sided score test as implemented in RVTESTS and RAREMETALS (Zhan et al.2016, PMID: 27153000).
[b] Mixed model logistic regression as implemented in SAIGE (Zhou et al. 2018, PMID: 30104761).
[c] Asymptotic *P* value for t-statistic from logistic regression model as implemented in PLINK (Purcell et al. 2007, PMID: 17701901).
[d] Two-sided Z-test as implemented in METAL (Willer et al. 2010, PMID: 20616382); all *P* values are uncorrected for the number of tests; genome-wide significance threshold was set at $P < 1\times10^{-8}$.
[e] Genome-wide significant *P* value of independently associated SNPs after conditioning on the lead variant. If three independent SNPs were identified, the 3rd one was conditioned on the first 2 variants.
[f] References for loci associated with eczema in previous GWAS ordered by publication date (PMID);
[1]Esparza-Gordillo et al. 2009 (19349984), [2]Sun et al. 2011 (21666691), [3]Paternoster et al. 2011 (22197932), [4]Hirota et al. 2012 (23042114), [5]Ellinghaus et al. 2013 (23727859), [6]Esparza-Gordillo et al. 2013 (23582566), [7]Weidinger et al. 2013 (23886662), [8]Baurecht et al. 2015 (25574825), [9]Paternoster et al. 2015 (26482879), [10]Kichaev et al. 2018 (30595370), [11]Johansson et al. 2019 (31361310)
[g] rs12123821 is in LD (D' = 0.78; r^2 = 0.19) with the exonic loss-of-function variant rs558269137 (FLG:2284del4, stopgain).

File Name: Supplementary Data 3.
Description: Rare and low-frequency variants associated with eczema at $P < 1x10^{-6}$ in the meta-analysis.
SNP, single nucleotide polymorphism; Chr, chromosome; Pos, genomic positions (GRCh37.p13); Ref, reference allele; Alt, alternative allele; AF, allele frequency; OR, odds ratio.
[a] Two-sided score test as implemented in RVTESTS and RAREMETALS (Zhan et al.2016, PMID: 27153000).

[b] Mixed model logistic regression as implemented in SAIGE (Zhou et al. 2018, PMID: 30104761).
[c] Asymptotic *P* value for t-statistic from logistic regression model as implemented in PLINK (Purcell et al. 2007, PMID: 17701901).
[d] Two-sided Z-test as implemented in METAL (Willer et al. 2010, PMID: 20616382); all *P* values are uncorrected for the number of tests; genome-wide significance threshold was set at $P < 1\times10^{-8}$.

File Name: Supplementary Data 4.
Description: Correlation between common and rare/low-frequency variants located at the same locus.
LD, linkage disequilibrium (D' and r^2); SNP, single nucleotide polymorphism; Chr, chromosome; Pos, genomic positions (GRCh37.p13); Ref, reference allele; Alt, alternative allele; AF, allele frequency; OR, odds ratio.
[a] Rare/low-frequency variants in bold.
[b] Two-sided score test as implemented in RVTESTS and RAREMETALS (Zhan et al.2016, PMID: 27153000).
[c] Mixed model logistic regression as implemented in SAIGE (Zhou et al. 2018, PMID: 30104761).
[d] Asymptotic *P* value for t-statistic from logistic regression model as implemented in PLINK (Purcell et al. 2007, PMID: 17701901).
[e] Two-sided Z-test as implemented in METAL (Willer et al. 2010, PMID: 20616382); all *P* values are uncorrected for the number of tests; genome-wide significance threshold was set a $P < 1\times10^{-8}$.
[f] Association *P* value of a common variant was conditioned on the rare variant identified at the same locus and vice versa.
[g] Association *P* value of the respective rare SNP was conditioned on the 2 common variants at the same locus. Likewise, *P* value of the respective common SNP was conditioned on the 2 rare variants at the same locus.

File Name: Supplementary Data 5.
Description: Replication results for 32 loci identified in previous GWAS on eczema.
SNP, single nucleotide polymorphism; Chr, chromosome; RA, risk allele; AA, alternative allele; AF, allele frequency; OR, odds ratio; LD, linkage disequilibrium; RefA, reference allele; NA, data not available; N.s., not significant.
[a] If available the lead SNP of the largest study (Paternoster et al. 2015, PMID: 26482879) is shown. If a SNP was not present in our study, a proxy is indicated (marked by an asterisk). For eczema-associated SNPs identified in Asian populations which did not replicate, the best-associated SNP from our study within a ±1-Mb window is shown (marked by a §).
[b] Genomic positions are based on human genome reference assembly GRCh37.p13.
[c] If in the original study the complementary sequence was reported for the respective SNP, the reference sequence is shown in parentheses.

[d] References (PMID) are Sun et al. 2011 (21666691), Paternoster et al. 2011 (22197932), Hirota et al. 2012 (23042114),Ellinghaus et al. 2013 (23727859), Schaarschmidt et al. 2015 (25865352), Paternoster et al. 2015 (26482879)
[e] Two-sided Z-test as implemented in METAL (Willer et al. 2010, PMID: 20616382); *P* values are uncorrected for the number of tests; replication threshold was set at $P < 0.0015$ (0.05/32) according to the number of SNPs tested.
[f] Pairwise LD (D' and r^2) between a reported lead SNP und a proxy SNP from our study was calculated by using LDlink (https://ldlink.nci.nih.gov/?tab=ldpair, selected population: EUR).

File Name: Supplementary Data 6.
Description: Different SNP selection strategies and association tests for the gene-based analyses in the RV set.
SNP, single nucleotide polymorphism; OR, odds ratio; CI, confidence interval; n, number of genes tested for each aggregation strategy; CADD, PHRED-scaled CADD score.
[a] Significant *P* values are labeled in bold. *P* values are two-sided and uncorrected for the number of tests performed. Significance thresholds for the different aggregation strategies were $P < 1.5 \times 10^{-4}$ (n = 322), $P < 4.0 \times 10^{-6}$ (n = 12,474), $P < 3.0 \times 10^{-6}$ (n = 16,538).
The results for three different aggregation strategies and two different gene-level association tests (GRANVIL and SKAT) are shown. Using the RV set, different marker selection strategies combining different sets of variants according to their annotations and functional prediction scores were tested; (i) loss-of-function (LOF) variants, ii) LOF plus missense variants, iii) variants with a high deleteriousness score (PHRED-scaled CADD score > 15). In addition, since the disease-causing allelic architecture is expected to be diverse across the genome, two different gene-level association tests were applied, GRANVIL and SKAT, which are implemented in the RAREMETALS software package. GRANVIL is a burden test that has its strengths when multiple deleterious variants within a gene are combined. SKAT, a variance-component test, has more power to identify association of a gene if both, risk and protective alleles are present. Both the variant selection strategy and the applied association test had an impact on the results. While the number of genes under study with at least two LOF variants was very low (n=322), the inclusion of missense variants diluted the results in genes like *FLG*, in which the number of missense variants with small and heterogeneous effects exceeded the number of a few variants with high impact. Selecting variants based on the CADD score counteracted both undesirable effects. In combination with the variance-component test it performed best for the different scenarios.

File Name: Supplementary Data 7.
Description: Gene-level association results.
CHR, chromosome; NSNP and NPARAM, number of single nucleotide polymorphisms and parameters respectively included in the analysis; N, effective sample size.
Best associated gene at each locus is indicated in bold. Genes represented by a single SNP are not shown.

[a] Two-sided Z-test as implemented in FUMA (Watanabe et al. 2017, PMID: 29184056); *P* values are uncorrected for the number of tests; significance threshold was set at $P < 3.8\text{x}10^{-6}$ (0.05/13,000) according to the number of genes tested.

File Name: Supplementary Data 8.
Description: Functional annotations of the identified rare/low-frequency variants.
SNP, single nucleotide polymorphism; LD, linkage disequilibrium; Chr, chromosome; Ref, reference allele; Alt, alternative allele; AF (EUR) allele frequency in Europeans; eQTL, expression quantitative trait locus; r^2 and *D'*, measures for LD of the indicated SNP (SNP in LD) with the lead SNP (associated SNP).
[a] Rare and low frequency variants from the single variant analysis and from the gene-level analysis (Table 1) are included. In addition, all SNPs in LD ($r^2 > 0.8$) with the associated SNPs are listed.
[b] Genomic positions are based on human genome reference assembly GRCh37.p13.

File Name: Supplementary Data 9.
Description: Gene-set analysis using all variants.
N Genes, number of genes; STD, standard deviation; SE, standard error
[a] Competetive gene-set analysis tests whether the genes in a gene-set are more strongly associated with the phenotype than other genes using a one-sided two-sample t-test as implemented in MAGMA (de Leeuw et al. 2015, PMID: 25885710). Uncorrected and Boferroni corrected *P* values are indicated.

File Name: Supplementary Data 10.
Description: Gene-set analysis for common and rare/low-frequency variants respectively.
N Genes, number of genes; STD, standard deviation; SE, standard error
[a] Competetive gene-set analysis tests whether the genes in a gene-set are more strongly associated with the phenotype than other genes using a one-sided two-sample t-test as implemented in MAGMA (de Leeuw et al. 2015, PMID: 25885710). Uncorrected and Boferroni corrected *P* values are indicated.

File Name: Supplementary Data 11.
Description: Heritability (observed scale) explained by different LD scores and minor allele frequency bins.
SNP, single nucleotide polymorphism; MAF, minor allele frequency; NSNPS, number of SNPs; SE, standard error; Q, quartiles based on LD scores of the SNPs; bins contributing significantly to the model in bold.
[a] Significant regions excluded were defined as ±500 Kb from significant variants ($P < 1\text{x}10^{-8}$) identified in the meta-analysis.
[b] Likelihood Ratio Test (LRT) comparing the full model (i.e. all bins included) to the reduced model (i.e. all bins except the bin tested); linear mixed models using genome-based restricted maximum likelihood (GREML) as implemented in GCTA (Yang et al. 2011 and 2015, PMID: 21167468 and 26323059).

File Name: Supplementary Data 12.

Description: Heritability (observed scale) explained by variants <5%.

SNP, single nucleotide polymorphism; MAF, minor allele frequency; NSNPS, number of SNPs; ALL, for each frequency bin, all LD score quartiles from Supplementary Data 11a were combined.

[a] Significant regions excluded were defined as ±500 Kb from significant variants ($P < 1x10^{-8}$) identified in the meta-analysis.

File Name: Supplementary Data 13.

Description: Imputation quality (r^2) of the identified rare/low-frequency variants in the 21 study populations.

[a] Best associated rare/low-frequency variants from the single variant analysis and significant variants from the gene-level test are shown.

[b] For CATSS, SALTY, FINNGEN, and UKBB the INFO score is reported.

File Name: Supplementary Data 14.

Description: Imputation quality of the identified exonic variants.

SNP, single nucleotide polymorphism; r^2, imputation quality in HRC-imputation; NRS, non-reference sensitivity; NRD, non-reference discordancy.

[a] Comparison of HRC-imputed genotypes vs. exome sequencing results in an in house data set (n = 892).
