# Supplementary material for: Rare variant analysis in eczema identifies exonic variants in DUSP1, NOTCH4 and SLC9A4
Source: Nat Commun. 2021 Nov 16;12:6618. doi: 10.1038/s41467-021-26783-x (PMC8595373; doi:10.1038/s41467-021-26783-x)
Supplement: Supplementary file 4 — Reporting Summary [file 41467_2021_26783_MOESM4_ESM.pdf]

nature portfolio

Corresponding author(s): Young-Ae Lee, NCOMMS-20-50003B

Last updated by author(s): Oct 15, 2021

Reporting Summary

Nature Portfolio wishes to improve the reproducibility of the work that we publish. This form provides structure for consistency and transparency in reporting. For further information on Nature Portfolio policies, see our Editorial Policies and the Editorial Policy Checklist.

Statistics

For all statistical analyses, confirm that the following items are present in the figure legend, table legend, main text, or Methods section.

n/a	Confirmed	
☐	☒	The exact sample size (n) for each experimental group/condition, given as a discrete number and unit of measurement
☐	☒	A statement on whether measurements were taken from distinct samples or whether the same sample was measured repeatedly
☐	☒	The statistical test(s) used AND whether they are one- or two-sided<br>*Only common tests should be described solely by name; describe more complex techniques in the Methods section.*
☐	☒	A description of all covariates tested
☐	☒	A description of any assumptions or corrections, such as tests of normality and adjustment for multiple comparisons
☐	☒	A full description of the statistical parameters including central tendency (e.g. means) or other basic estimates (e.g. regression coefficient) AND variation (e.g. standard deviation) or associated estimates of uncertainty (e.g. confidence intervals)
☐	☒	For null hypothesis testing, the test statistic (e.g. F, t, r) with confidence intervals, effect sizes, degrees of freedom and P value noted<br>*Give P values as exact values whenever suitable.*
☒	☐	For Bayesian analysis, information on the choice of priors and Markov chain Monte Carlo settings
☒	☐	For hierarchical and complex designs, identification of the appropriate level for tests and full reporting of outcomes
☐	☒	Estimates of effect sizes (e.g. Cohen's d, Pearson's r), indicating how they were calculated

Our web collection on statistics for biologists contains articles on many of the points above.

Software and code

Policy information about availability of computer code

Data collection	*Provide a description of all commercial, open source and custom code used to collect the data in this study, specifying the version used OR state that no software was used.*
Data analysis	*Provide a description of all commercial, open source and custom code used to analyse the data in this study, specifying the version used OR state that no software was used.*

For manuscripts utilizing custom algorithms or software that are central to the research but not yet described in published literature, software must be made available to editors and reviewers. We strongly encourage code deposition in a community repository (e.g. GitHub). See the Nature Portfolio guidelines for submitting code & software for further information.

Data

Policy information about availability of data

All manuscripts must include a data availability statement. This statement should provide the following information, where applicable:

- Accession codes, unique identifiers, or web links for publicly available datasets
- A description of any restrictions on data availability
- For clinical datasets or third party data, please ensure that the statement adheres to our policy

Summary statistics of the meta-analysis which were generated in this study have been deposited in the GWAS Catalog (https://www.ebi.ac.uk/gwas/home) under accession code “GCST90044763”.

Field-specific reporting

Please select the one below that is the best fit for your research. If you are not sure, read the appropriate sections before making your selection.

- [x] Life sciences
- [ ] Behavioural & social sciences
- [ ] Ecological, evolutionary & environmental sciences

For a reference copy of the document with all sections, see nature.com/documents/nr-reporting-summary-flat.pdf

Life sciences study design

All studies must disclose on these points even when the disclosure is negative.

Sample size	Genomewide association study including 20,016 eczema cases and 380,433 controls. A power calculation is provided in Supplementary Figure 1.
Data exclusions	Samples/genotypes were excluded based on standard quality criteria as described in detail for each data set in Supplementary Note 1.
Replication	We performed a more powerful single-stage design without a replication phase. All available study populations of European ancestry (from Europe, the U.S., and Australia) with proper phenotype definitions were included in this study. Additional study populations of adequate size and with comparable phenotype for replication are not available.
Randomization	not applicable
Blinding	not applicable

Reporting for specific materials, systems and methods

We require information from authors about some types of materials, experimental systems and methods used in many studies. Here, indicate whether each material, system or method listed is relevant to your study. If you are not sure if a list item applies to your research, read the appropriate section before selecting a response.

Materials & experimental systems

n/a	Involved in the study	
x		Antibodies
x		Eukaryotic cell lines
x		Palaeontology and archaeology
x		Animals and other organisms
	x	Human research participants
x		Clinical data
x		Dual use research of concern

Methods

n/a	Involved in the study	
x		ChIP-seq
x		Flow cytometry
x		MRI-based neuroimaging

Human research participants

Policy information about studies involving human research participants

Population characteristics	21 data sets comprising 20,016 eczema cases and 380,433 controls aged >0,5 years of age, unselected sex, with data on eczema diagnosis and genome-wide genotype data obtained from commercial genotyping chips. There was no treatment information available.
Recruitment	The two largest data sets were the UK Biobank and FINNGEN cohorts with population-based recruitment schemes. In addition, we investigated population-based birth cohorts (e.g. ALSPAC, MAAS). The other data sets included established eczema cohorts and ethnically matched controls.
Ethics oversight	Each participating study obtained the approval of the local Ethics Board.

Note that full information on the approval of the study protocol must also be provided in the manuscript.
